# Sex Differences in Long COVID

**DOI:** 10.1001/jamanetworkopen.2024.55430

**Published:** 2025-01-22

**Authors:** Dimpy P. Shah, Tanayott Thaweethai, Elizabeth W. Karlson, Hector Bonilla, Benjamin D. Horne, Janet M. Mullington, Juan P. Wisnivesky, Mady Hornig, Daniel J. Shinnick, Jonathan D. Klein, Nathaniel B. Erdmann, Shari B. Brosnahan, Joyce K. Lee-Iannotti, Torri D. Metz, Christine Maughan, Ighovwerha Ofotokun, Harrison T. Reeder, Lauren E. Stiles, Aasma Shaukat, Rachel Hess, Hassan Ashktorab, Logan Bartram, Ingrid V. Bassett, Jacqueline H. Becker, Hassan Brim, Alexander W. Charney, Tananshi Chopra, Rebecca G. Clifton, Steven G. Deeks, Kristine M. Erlandson, Daniel S. Fierer, Valerie J. Flaherman, Vivian Fonseca, Jennifer C. Gander, Sally L. Hodder, Vanessa L. Jacoby, Pavitra Kotini-Shah, Jerry A. Krishnan, Andre Kumar, Bruce D. Levy, David Lieberman, Jenny J. Lin, Jeffrey N. Martin, Grace A. McComsey, Talal Moukabary, Megumi J. Okumura, Michael J. Peluso, Clifford J. Rosen, George Saade, Pankil K. Shah, Zaki A. Sherif, Barbara S. Taylor, Katherine R. Tuttle, Alfredo E. Urdaneta, Julie A. Wallick, Zanthia Wiley, David Zhang, Leora I. Horwitz, Andrea S. Foulkes, Nora G. Singer

**Affiliations:** 1 Long School of Medicine, University of Texas Health Science Center, San Antonio; 2 Massachusetts General Hospital Biostatistics, Somerville; 3 Department of Medicine, Harvard Medical School, Boston, Massachusetts; 4 Brigham and Women’s Hospital, Boston, Massachusetts; 5 Division of Infectious Diseases, Department of Medicine, Stanford University, Palo Alto, California; 6 Intermountain Heart Institute, Intermountain Health, Salt Lake City, Utah; 7 Division of Cardiovascular Medicine, Department of Medicine, Stanford University, Stanford, California; 8 Department of Neurology, Beth Israel Deaconess Medical Center, Boston, Massachusetts; 9 Division of General Internal Medicine, Icahn School of Medicine at Mount Sinai, New York, New York; 10 Columbia University Mailman School of Public Health, New York, New York; 11 RECOVER Patient, Caregiver, or Community Representative, New York, New York; 12 Department of Pediatrics, Stanford University, Palo Alto, California; 13 Illinois Research Network, University of Illinois Chicago; 14 Division of Infectious Diseases, Department of Medicine, University of Alabama at Birmingham; 15 Department of Medicine, NYU Grossman School of Medicine, New York, New York; 16 Department of Neurology, University of Arizona College of Medicine, Phoenix; 17 Department of Internal Medicine, University of Arizona College of Medicine, Phoenix; 18 Department of Obstetrics and Gynecology, University of Utah Health, Salt Lake City; 19 Division of Infectious Diseases, Department of Medicine, Emory University School of Medicine, Atlanta, Georgia; 20 Stony Brook University Renaissance School of Medicine, Stony Brook, New York; 21 Department of Population Health Sciences, Spencer Fox Eccles School of Medicine at the University of Utah, Salt Lake City; 22 Department of Internal Medicine, Spencer Fox Eccles School of Medicine at the University of Utah, Salt Lake City; 23 Howard University College of Medicine, Washington, DC; 24 Department of Medicine, Massachusetts General Hospital, Boston; 25 Cedars-Sinai Medical Center, Los Angeles, California; 26 Department of Epidemiology, The George Washington University, Washington, DC; 27 Department of Medicine, University of California San Francisco; 28 Division of Infectious Diseases, Department of Medicine, University of Colorado–Anschutz Medical Campus, Aurora; 29 Department of Pediatrics, University of California San Francisco; 30 Department of Epidemiology and Biostatistics, University of California San Francisco; 31 Department of Medicine, Tulane University Health Sciences Center, New Orleans, Louisiana; 32 Center for Research and Evaluation, Kaiser Permanente of Georgia, Atlanta; 33 Centre College, Danville, Kentucky; 34 Department of Medicine, West Virginia University, Morgantown; 35 Banner Health, Tucson, Arizona; 36 University Hospitals Cleveland Medical Center, Case Western Reserve University School of Medicine, Cleveland, Ohio; 37 University of Arizona, Tucson; 38 MaineHealth Institute for Research, Scarborough; 39 Department of Obstetrics and Gynecology, The University of Texas Medical Branch, Galveston; 40 Providence Inland Northwest Health, Spokane, Washington; 41 Department of Emergency Medicine, Division of Emergency Critical Care, Stanford University, Palo Alto, California; 42 Swedish Health Services, Seattle, Washington; 43 Biological Sciences Division, University of Chicago, Chicago, Illinois; 44 MetroHealth Medical Center, Cleveland

## Abstract

**Importance:**

A substantial number of individuals worldwide experience long COVID, or post-COVID condition. Other postviral and autoimmune conditions have a female predominance, but whether the same is true for long COVID, especially within different subgroups, is uncertain.

**Objective:**

To evaluate sex differences in the risk of developing long COVID among adults with SARS-CoV-2 infection.

**Design, Setting, and Participants:**

This cohort study used data from the National Institutes of Health (NIH) Researching COVID to Enhance Recovery (RECOVER)–Adult cohort, which consists of individuals enrolled in and prospectively followed up at 83 sites in 33 US states plus Washington, DC, and Puerto Rico. Data were examined from all participants enrolled between October 29, 2021, and July 5, 2024, who had a qualifying study visit 6 months or more after their initial SARS-CoV-2 infection.

**Exposure:**

Self-reported sex (male, female) assigned at birth.

**Main Outcomes and Measures:**

Development of long COVID, measured using a self-reported symptom-based questionnaire and scoring guideline at the first study visit that occurred at least 6 months after infection. Propensity score matching was used to estimate risk ratios (RRs) and risk differences (95% CIs). The full model included demographic and clinical characteristics and social determinants of health, and the reduced model included only age, race, and ethnicity.

**Results:**

Among 12 276 participants who had experienced SARS-CoV-2 infection (8969 [73%] female; mean [SD] age at infection, 46 [15] years), female sex was associated with higher risk of long COVID in the primary full (RR, 1.31; 95% CI, 1.06-1.62) and reduced (RR, 1.44; 95% CI, 1.17-1.77) models. This finding was observed across all age groups except 18 to 39 years (RR, 1.04; 95% CI, 0.72-1.49). Female sex was associated with significantly higher overall long COVID risk when the analysis was restricted to nonpregnant participants (RR, 1.50; 95%: CI, 1.27-1.77). Among participants aged 40 to 54 years, the risk ratio was 1.42 (95% CI, 0.99-2.03) in menopausal female participants and 1.45 (95% CI, 1.15-1.83) in nonmenopausal female participants compared with male participants.

**Conclusions and Relevance:**

In this prospective cohort study of the NIH RECOVER-Adult cohort, female sex was associated with an increased risk of long COVID compared with male sex, and this association was age, pregnancy, and menopausal status dependent. These findings highlight the need to identify biological mechanisms contributing to sex specificity to facilitate risk stratification, targeted drug development, and improved management of long COVID.

## Key Points

**Question:**

Does the risk of long COVID, or post-COVID condition, differ by sex?

**Findings:**

In this cohort study of 12 276 individuals, females had a significantly higher risk of long COVID compared with males after adjusting for sociodemographic and clinical risk factors. The sex-based difference in long COVID risk was age, pregnancy, and menopause dependent, with the highest risk among females aged 40 to 55 years.

**Meaning:**

These findings highlight the importance of evaluating differences in risk of long COVID after SARS-CoV-2 infection in males and females and of comparing biological mechanisms that may underlie sexually dimorphic long COVID trajectories.

## Teaser

This cohort study examines whether there are differences in the risk of developing long COVID, or post-COVID condition, by sex.

## Introduction

Worldwide, SARS-CoV-2 has infected more than 700 million individuals, with an estimated 7 million deaths.^1^ Although many individuals recover from acute COVID-19, a substantial proportion experience long-term effects,^2,3,4^ termed long COVID, or post-COVID condition or postacute sequelae of SARS-CoV-2 infection. However, like the variation in acute COVID-19 severity, the risk of long COVID may differ among individuals.

Biological sex appears to be a source of variability in the development, presentation, and longitudinal trajectories of long COVID. Numerous studies have shown that males have more severe acute COVID-19 cases and higher mortality than females.^5,6^ However, emerging literature suggests that females may be at greater risk for new and persisting symptoms following SARS-CoV-2 infection. Two systematic reviews and meta-analyses found odds ratios of 1.52 (95% CI, 1.27-1.82) to 1.56 (95% CI, 1.41-1.73) for the development of long COVID among females compared with males.^7,8^

Studies have yet to fully account for factors that may distort the true estimate of biological sex–related risk of long COVID (eg, age, menstrual status, comorbidities, vaccination status, variants of concern, severity of acute illness, and differential engagement in health care). Some studies also relied on relatively small sample sizes or samples lacking ethnic or racial diversity. Thus, significant knowledge gaps still exist in the literature concerning the sex-related risk of long COVID. We analyzed data from the National Institutes of Health (NIH)–funded Researching COVID to Enhance Recovery (RECOVER)–Adult cohort, the largest cohort to date followed up in a natural history study of long COVID; it includes representation across the US and records systematically collected robust symptom and clinical data from all participants. Understanding differences in the development of long COVID across female subgroups is an important first step in identifying biological mechanisms and sexual dimorphism. Such an understanding can help advance the development of effective interventions, clinical practice guidelines, and public health policies to alleviate the burden of long COVID. We aimed to evaluate differences in the risk of long COVID between male and female RECOVER-Adult participants with a history of SARS-CoV-2 infection, adjusting for other baseline sociodemographic and clinicopathologic risk factors, including the severity of the initial SARS-CoV-2 infection and variant era.

## Methods

### Study Design

The RECOVER-Adult cohort has been previously described.^9^ Participants were enrolled at 83 sites in 33 states plus Washington, DC, and Puerto Rico and then prospectively followed up. Participants at least 18 years of age were eligible to enroll regardless of previous infection with SARS-CoV-2. Participants completed symptom survey questionnaires every 3 months and had an in-person physical examination and laboratory studies at least once annually. The current study was approved by the NYU Langone Health institutional review board (IRB), which served as a single IRB for most sites, while others required local IRB approval; all participants provided written informed consent prior to enrollment. The study followed the Strengthening the Reporting of Observational Studies in Epidemiology (STROBE) reporting guideline for cohort studies.

### Study Population

Adult participants with a history of SARS-CoV-2 infection who enrolled in RECOVER-Adult were eligible for analysis (Figure). Participants enrolled in RECOVER-Adult between October 29, 2021, and July 5, 2024. Enrollment has concluded, but follow-up is ongoing; data were locked as of September 6, 2024. The index infection was defined as the first reported SARS-CoV-2 infection.^10^ The included cohorts were categorized based on acute (participants enrolled within 30 days after the index infection) or postacute (participants enrolled more than 30 days after the index infection) period. Participants who were uninfected at enrollment and had a positive antibody result (nucleocapsid for any participant or spike protein for those who were unvaccinated) at enrollment were reclassified as infected and assigned an index infection date 90 days prior to the positive antibody test result. Participants enrolled as uninfected who had an infection while enrolled were classified as crossover participants and were included in the acute enrollment subcohort for analyses.^10^

**Figure.**
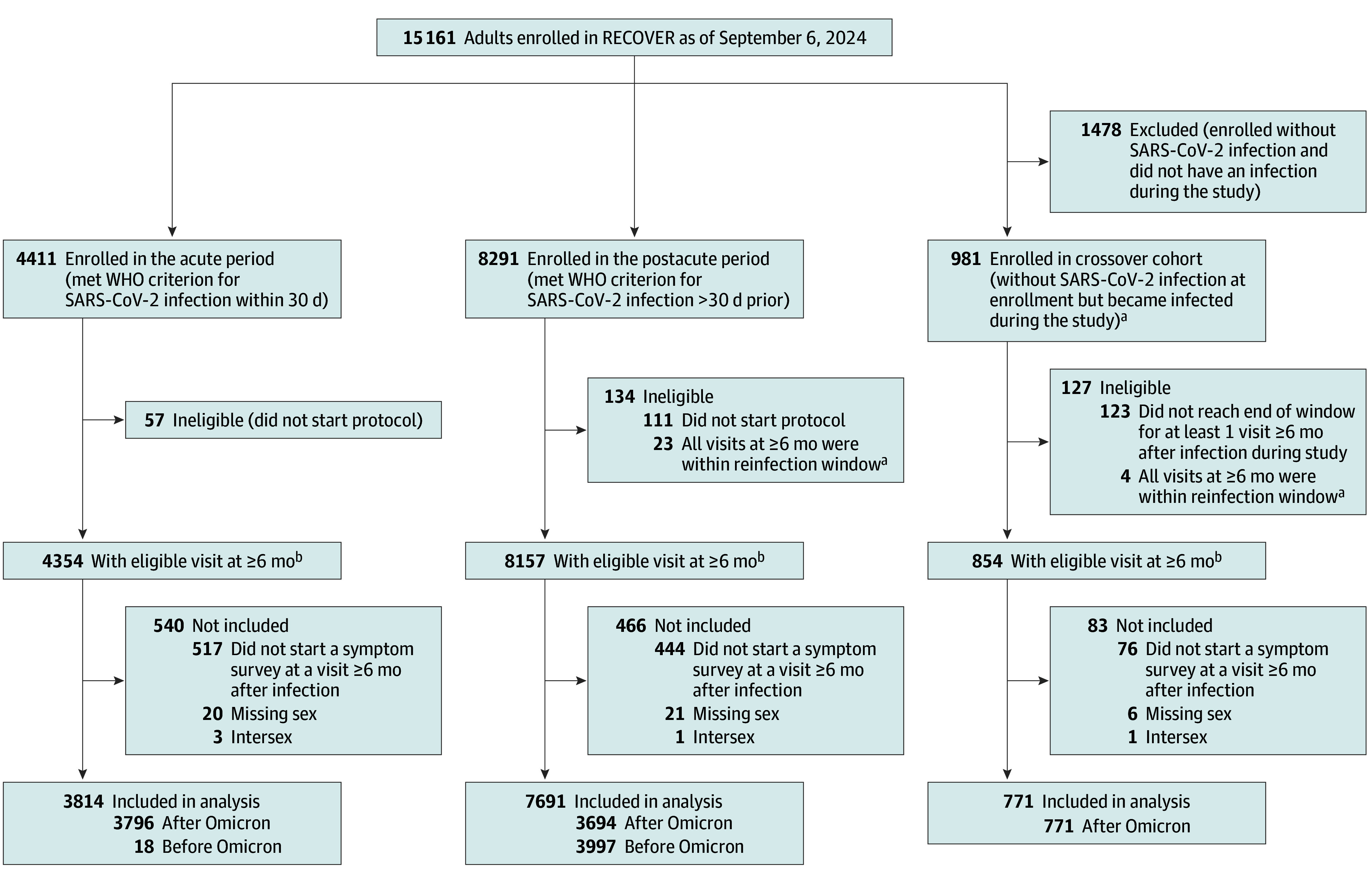
Application of Inclusion and Exclusion Criteria to Define the Study Cohort RECOVER indicates Researching COVID to Enhance Recovery; WHO, World Health Organization. ^a^The reinfection window for exclusion was 30 days prior to and 7 days after the visit. ^b^Participants who completed the visit without reaching the end of the visit window are included in this count.

Participants were excluded from the analysis if they lacked a history of SARS-CoV-2 infection or did not respond to surveys 4.5 months or more beyond their index infection. Visits were not included in the analysis if reinfection occurred up to 30 days before or 7 days after the visit. Participants were also excluded if they reported being assigned as intersex at birth or were missing data regarding sex assigned at birth.

Participant race and ethnicity were ascertained by self-report via a standardized instrument and were included in the study because race and ethnicity are important factors in development of long COVID. Categories included Hispanic, non-Hispanic Asian (hereafter, Asian), non-Hispanic Black (hereafter, Black), non-Hispanic White (hereafter, White), multiracial and other (American Indian or Alaska Native, Native Hawaiian or Other Pacific Islander). Full details are given in the eMethods in Supplement 1.

### Exposure and Outcomes

The exposure was self-reported sex assigned at birth. All participants completed comprehensive symptom surveys with associated severity questions at each study visit. The outcome was the presence of long COVID at the first visit 6 months or more after the index infection ascertained using a previously reported symptom-based scoring algorithm (ie, research index)^10^ that was updated in 2024.^11^ The symptoms contributing to the long COVID definition included postexertional malaise, fatigue, brain fog, dizziness, palpitations, loss of or change in smell or taste, thirst, chronic cough, chest pain, shortness of breath, and snoring or sleep apnea. Participants with a research index of 11 or greater were considered long COVID positive; the rest were classified as long COVID indeterminate (not meeting classification criteria for long COVID but not necessarily asymptomatic).^10,11^ Participants positive for long COVID were assigned to long COVID subphenotypes based on Euclidean distance to the cluster centroid of each subphenotype.^10,11^

### Statistical Analysis

In sex-stratified analyses, we summarized (1) demographic and clinical characteristics, (2) individual symptom frequencies, and (3) the proportion of participants meeting long COVID criteria. We calculated mean and median long COVID research indices and the distribution of long COVID subphenotypes among participants meeting long COVID criteria.

We used propensity score matching to perform a controlled comparison of the risk of long COVID between participants assigned female or male sex at birth. All factors contributing to a propensity score do not need to be confounders. Propensity score matching balances the 2 populations being compared on all adjustment factors even when the analysis is noncausal.^12^ Variables contributing to the propensity score model included demographic and enrollment factors, social determinants of health, hospitalization status during the first infection, and vaccination status at the first infection. Demographic and enrollment factors included age (modeled using cubic splines), race and ethnicity, era of infection, method of referral to RECOVER, and time between enrollment and infection (eMethods in Supplement 1). We also fit a primary reduced propensity score model that included only age at infection and race and ethnicity, thus excluding all variables that could be considered potentially downstream of sex assigned at birth. We handled missing covariate data for estimating and calculating propensity scores using multiple imputation (M = 10). Data were assumed to be missing at random. We used full matching within each imputed dataset, which used all participants and assigned weights to each subclass created by matching.^13,14^ Using these weights, we estimated risk ratios (RRs) using Poisson regression with robust SEs and absolute risk differences (RDs) using binomial regression with an identity link.^15^ We combined model estimates and averaged results across multiply imputed datasets. For data visualization, we generated Love plots to evaluate balance in covariates before and after propensity score matching.^16^

Two secondary analyses are presented. First, the propensity score matching procedure was performed within age strata (18-39, 40-54, and ≥55 years). Second, the propensity score matching procedure was repeated within strata defined by age and menopausal status. Menopause was defined based on participant reports (eMethods in Supplement 1). Participants who were pregnant at the time of the follow-up visit from which data were used in the analysis or who reported having had a hysterectomy were excluded from menopause-stratified analyses. Three strata among female participants were defined: nonmenopausal, aged 18 to 39 years; nonmenopausal, aged 40 to 54 years; and menopausal, aged 40 to 54 years. Females meeting each specified age and menopausal criterion were compared with males meeting the same age criterion.

Five sensitivity analyses were performed. First, the propensity score analysis was repeated in the subcohort enrolled in the acute period; these participants were followed up prospectively from the time of infection and were the least subject to selection bias based on having enrolled prior to any presence of long COVID. Second, participants who were pregnant at any time between their index date and their follow-up study visit were excluded. Third, comorbidities were added to the propensity score model to impose additional balance on these factors (Table 1). The rationale for not including comorbidities in the primary model is that they may be in the causal pathway (eg, they are mechanistically related to biological sex and may be risk factors for long COVID^17,18^). Fourth, the analysis was repeated after stratifying by pandemic wave (before Omicron, defined as index infection before December 1, 2021, and Omicron, defined as index infection on December 1, 2021, or later) because the Omicron variant had been reported to be less severe clinically.^19,20^ Finally, the analysis was repeated after stratifying for hospitalization during the index infection because the severity of acute infection may be associated both with the risk of long COVID and with sex. For stratified analyses, stratification factors were removed from their respective propensity score models.

**Table 1.** Study Participant Characteristics

Characteristic	Participants^a^		
	Female (n = 8969)	Male (n = 3307)	Overall (N = 12 276)
Age at infection, y			
Mean (SD)	45 (15)	51 (16)	46 (15)
Median (IQR)	42 (32-56)	52 (38-63)	45 (33-59)
Missing data, No.	3	1	4
Race and ethnicity^b^			
Hispanic	1614 (18)	559 (17)	2173 (18)
Non-Hispanic Asian	481 (5)	225 (7)	706 (6)
Non-Hispanic Black	1296 (14)	421 (13)	1717 (14)
Non-Hispanic White	5115 (57)	1933 (58)	7048 (57)
Multiracial, other, or missing	463 (5)	169 (5)	632 (5)
Infection cohort			
Enrolled in the acute period	2621 (29)	1193 (36)	3814 (31)
Crossover	583 (7)	188 (6)	771 (6)
Enrolled in the postacute period	5765 (64)	1926 (58)	7691 (63)
Enrollment subcohort and era			
Before Omicron	2991 (33)	1024 (31)	4015 (33)
Acute Omicron^c^	3192 (36)	1375 (42)	4567 (37)
Postacute Omicron	2786 (31)	908 (27)	3694 (30)
Referral type			
Community outreach	2310 (26)	752 (23)	3062 (25)
Public health department list	297 (3)	120 (4)	417 (3)
Community health center	160 (2)	64 (2)	224 (2)
Participant tested and treated in the health system	3194 (36)	1123 (34)	4317 (35)
Existing, prospectively followed-up COVID-19 cohort	545 (6)	246 (7)	791 (6)
Existing non–COVID-19 research or clinical cohort	122 (1)	81 (2)	203 (2)
Long COVID clinic	195 (2)	72 (2)	267 (2)
Self-referral from RECOVER website or other unsolicited self-referral	2141 (24)	844 (26)	2985 (24)
Missing data, No.	5	5	10
Hospitalization			
Hospitalized during acute phase of first infection	556 (7)	306 (10)	862 (8)
Missing data, No.	556	233	789
Vaccination status at first infection			
Unvaccinated	2777 (31)	929 (29)	3706 (31)
Partially vaccinated or date of last dose unknown	359 (4)	136 (4)	495 (4)
Fully vaccinated	5702 (65)	2180 (67)	7882 (65)
Missing data, No.	131	62	193
Visit month			
6-9	5551 (62)	2244 (68)	7795 (63)
12-21	2108 (24)	716 (22)	2824 (23)
≥24	1310 (15)	347 (10)	1657 (13)
Comorbidities^d^			
Immunocompromised condition	392 (4)	374 (11)	766 (6)
Rheumatologic, autoimmune, or connective tissue disease	1079 (12)	225 (7)	1304 (11)
Current cancer or ongoing cancer treatment	187 (2)	136 (4)	323 (3)
Chronic liver disease	116 (1)	85 (3)	201 (2)
Obesity	2662 (30)	697 (21)	3359 (28)
Diabetes	728 (8)	414 (13)	1142 (9)
Kidney disease	196 (2)	160 (5)	356 (3)
Cardiovascular disease	1582 (18)	1027 (32)	2609 (22)
Stroke	140 (2)	91 (3)	231 (2)
Asthma	1838 (21)	447 (14)	2285 (19)
Lung disease	329 (4)	175 (5)	504 (4)
Dementia	156 (2)	53 (2)	209 (2)
Mental health disorder	3544 (40)	852 (26)	4396 (36)
Chronic pain syndrome or fibromyalgia	632 (7)	124 (4)	756 (6)
ME/CFS	150 (2)	32 (1)	182 (2)
POTS	138 (2)	13 (0)	151 (1)
Neurological condition	513 (6)	275 (8)	788 (6)

Abbreviations: ME/CFS, myalgic encephalomyelitis/chronic fatigue syndrome; POTS, postural orthostatic tachycardia syndrome; RECOVER, Researching COVID to Enhance Recovery.

^a^ Data are presented as number (percentage) excluding those with missing data unless otherwise indicated.

^b^ Race and ethnicity categories were based on self-report via a standardized instrument. Other includes those who identified as American Indian or Alaska Native or as Native Hawaiian or Other Pacific Islander. See the eMethods in Supplement 1 for full details.

^c^ Includes crossover participants, as all were infected during the Omicron era.

^d^ Proportions exclude participants with missing data, which ranged from 1.2% to 1.4% among male participants and 0.9% to 1.3% among female participants.

We constructed 95% CIs for all comparisons; statistical significance was determined by whether the interval included the null value (1 for RR, 0 for RD). All analyses were performed in R, version 4.4.0 (R Project for Statistical Computing).^21^ Multiple imputation was performed using the mice package in R, version 3.16.0.^22^ Propensity score matching was performed using the matchthem package in R, version 1.2.1.^23^ The RRs and RDs were estimated using the survey package in R, version 4.4-2.^24^

## Results

### Patient Characteristics

The analysis cohort included 12 276 participants (8969 [73%] female and 3307 [27%] male; mean [SD] age at infection, 46 [15] years). Of these, 3814 (31%) were enrolled in the acute period, 7691 (63%) in the postacute period, and 771 (6%) were crossover participants (Figure). A total of 706 (6%) were Asian; 1717 (14%), Black; 2173 (18%), Hispanic; 7048 (57%), White; and 632 (5%), multiracial, other, or missing data. Male participants were older than female participants (median age at index infection, 52 years [IQR, 38-63 years] vs 42 years [IQR, (32-56 years]); had lower rates of obesity (697 [21%] vs 2662 [30%]), asthma (447 [14%] vs 1838 [21%]), and mental health conditions (852 [26%] vs 3544 [40%]); and had higher rates of hospitalization during acute infection (306 of 3074 [10%] vs 556 of 8413 [7%]) (Table 1). Rates of missing data were not different by sex (Table 1). eTable 2 in Supplement 1 reports the social determinants of health of participants included in the primary analysis.

### Symptoms and Long COVID by Sex

Symptom frequencies stratified by sex are provided in Table 2. A significantly higher proportion of females (1845 [21%]) compared with males (532 [16%]) had long COVID at the analysis visit (Table 3). The mean and median long COVID indices among long COVID–positive participants were slightly higher among females (mean [SD], 16.5 [4.7]; median, 15 [IQR, 13-20]) than among males (mean [SD], 15.9 [4.3]; median, 15 [IQR, 12-18]). The distribution of long COVID subphenotypes by sex is shown in Table 3. Symptom frequency by sex and long COVID subphenotype is shown in eFigure 4 in Supplement 1.

**Table 2.** Symptom Frequencies Stratified by Sex and Long COVID Status

Symptom	Frequency, %^a^				Difference in frequency between long COVID–positive females and males, percentage points
	Females (n = 8969)		Males (n = 3307)		
	Long COVID indeterminate (n = 7124)	Long COVID postive (n = 1845)	Long COVID indeterminate (n = 2775)	Long COVID positive (n = 532)	
**Symptoms contributing to the long COVID research index**					
Loss of smell or taste	3.8	41.4	2.8	36.3	5.0
Postexertional malaise	10.3	88.6	9.9	88.3	0.3
Chronic cough	4.7	32.6	6.0	38.2	−5.6
Brain fog	7.4	64.2	5.6	59.5	4.7
Shortness of breath	3.0	39.3	2.4	33.5	5.8
Palpitation	10.9	62.8	6.9	46.7	16.2
Dizziness	11.4	66.2	9.1	66.5	−0.2
Chest pain	2.5	27.0	2.7	26.1	0.9
Fatigue	25.8	86.7	19.3	83.2	3.5
Thirst	7.4	38.1	5.0	36.2	1.9
Sleep apnea	9.8	36.8	15.8	45.5	−8.7
Abnormal movements	1.3	13.8	1.5	15.7	−1.9
Sleep disturbance	5.4	32.4	4.8	31.6	0.8
**Symptoms not contributing to the long COVID research index**					
Sick from triggers	6.9	45.5	2.8	23.2	22.3
GI symptoms	18.8	64.0	12.3	45.9	18.0
Hair loss	15.6	38.6	9.6	22.4	16.2
Dry eyes	11.0	38.3	6.0	24.6	13.7
Itching	8.9	35.4	9.0	21.7	13.6
Fever, sweats, or chills	6.4	38.8	3.2	25.8	13.0
Postexertional soreness	13.3	77.6	12.0	64.7	12.9
Feeling hot or cold	13.1	55.6	6.2	46.4	9.2
Abdominal pain	2.6	21.2	1.5	12.4	8.8
Headaches	7.6	41.8	3.1	33.3	8.5
Swelling of legs	7.2	30.0	6.8	21.8	8.2
Bladder symptoms	9.1	32.2	6.9	24.0	8.2
Skin color changes	2.9	22.9	2.7	16.9	6.0
Back pain	9.5	40.4	7.5	35.4	5.0
Dry mouth	7.8	37.7	6.6	32.8	4.9
Joint pain	9.7	44.6	8.1	40.0	4.6
Muscle pain	7.2	41.0	5.3	36.6	4.5
Mouth pain	0.6	8.6	0.4	4.2	4.4
Fertility	2.5	5.6	0.8	1.9	3.7
Throat pain	0.7	11.9	0.4	8.8	3.1
Problems with teeth	7.6	27.5	8.6	24.7	2.8
Foot pain	3.8	24.3	4.6	21.7	2.5
Vision problems	3.9	32.1	3.6	29.7	2.4
Skin pain	1.0	11.4	0.6	9.5	1.9
Paralysis	0.5	5.3	0.8	3.6	1.7
Pelvic or genital pain	1.3	8.0	0.8	6.3	1.7
Numbness or tingling	0.3	5.9	0.8	4.4	1.6
Skin rash	5.5	20.2	5.1	18.8	1.4
Anxiety	7.2	27.5	4.5	26.5	1.0
Nerve problems, unspecified	0.2	1.0	0.3	0.4	0.6
Pain, unspecified	0.4	0.6	0.3	0.6	0.0
Cold limbs	11.3	39.1	7.8	39.1	−0.1
Tremor	2.5	19.6	3.3	19.9	−0.3
Seizures	0.1	1.1	0.1	1.9	−0.8
Weakness	4.2	42.6	5.2	43.5	−0.9
Anxiety and/or depression	7.5	34.2	4.7	35.4	−1.2
Hearing problems	12.5	44.9	18.7	46.8	−1.9
Anaphylaxis	1.1	4.3	0.7	7.2	−2.9
Depression	5.5	28.2	5.0	32.7	−4.5
Change in sexual desire or capacity	13.4	38.8	10.4	45.2	−6.4
Menstrual cycle changes	17.7	41.5	NA	NA	NA
Menopause	12.9	34.9	NA	NA	NA

Abbreviations: GI, gastrointestinal; NA, not applicable.

^a^ The frequency is the proportion of participants who reported each symptom at the study visit, stratified by long COVID status. Symptoms are ordered by contribution to the long COVID research index and then the difference in female long COVID frequency and male long COVID frequency.

**Table 3.** Long COVID Positivity and Distribution of Long COVID Research Index and Long COVID Subphenotypes Among Long COVID–Positive Participants Stratified by Sex Assigned at Birth

	Participants	
	Female (n = 8969)	Male (n = 3307)
Long COVID positive, No. (%)	1845 (21)	532 (16)
Long COVID research index among long COVID–positive participants^a^		
Mean (SD)	16.5 (4.7)	15.9 (4.3)
Median (IQR)	15 (13-20)	15 (12-18)
Subphenotype cluster among long COVID–positive participants, No./total No. (%)^b^		
1	397/1845 (22)	112/532 (21)
2	202/1845 (11)	80/532 (15)
3	325/1845 (18)	148/532 (28)
4	527/1845 (29)	105/532 (20)
5	394/1845 (21)	87/532 (16)

^a^ Long COVID research indices range from 0 to 30; the higher the score, the more likely that long COVID is present.

^b^ The 5 long COVID subphenotypes have been defined and characterized by Thaweethai et al^10^ and refined by Geng et al.^11^

### Propensity Score Matching Results

The propensity score distributions had high overlap (eFigure 1 in Supplement 1). The cohort was well balanced on the covariates used to fit the propensity scores after full matching (eFigure 2 in Supplement 1). Estimated RRs and RDs following propensity score matching are shown in Table 4. Overall, female compared with male sex was associated with higher risk of long COVID in the primary full model (RR, 1.31; 95% CI, 1.06-1.62) and in the primary reduced model (RR, 1.44; 95% CI, 1.17-1.77). Female sex was associated with an increase in absolute risk of long COVID in the primary full (RD, 0.05; 95% CI, 0.01-0.08) and reduced (RD, 0.06; 95% CI, 0.03-0.09) models.

**Table 4.** Estimated RRs and RDs Comparing Participants Assigned Female Compared With Male at Birth Using Propensity Score Matching^a^

Analysis	Sample size, No.		RR (95% CI)	RD (95% CI)	Estimated proportion with long COVID after matching, (95% CI)	
	Females	Males			Females	Males
**Primary**						
Full model	8969	3307	1.31 (1.06-1.62)	0.05 (0.01 to 0.08)	0.20 (0.19-0.21)	0.16 (0.12-0.19)
Reduced model	8969	3307	1.44 (1.17-1.77)	0.06 (0.03 to 0.09)	0.20 (0.19-0.21)	0.14 (0.11-0.17)
**Secondary**						
Age, y						
18-39	4097	959	1.04 (0.72-1.49)	0.00 (−0.05 to 0.06)	0.16 (0.14-0.17)	0.15 (0.10-0.21)
40-54	2439	900	1.48 (1.19-1.84)	0.09 (0.05 to 0.13)	0.28 (0.26-0.30)	0.19 (0.15-0.23)
≥55	2430	1447	1.34 (1.11-1.61)	0.05 (0.02 to 0.09)	0.21 (0.19-0.23)	0.16 (0.13-0.19)
Menopause status by age^b^						
Nonmenopausal, 18-39 y	3640	959	1.10 (0.83-1.46)	0.01 (−0.03 to 0.06)	0.16 (0.15-0.17)	0.14 (0.11-0.18)
40-54 y						
Nonmenopausal	1483	900	1.45 (1.15-1.83)	0.08 (0.03 to 0.13)	0.26 (0.23-0.29)	0.18 (0.15-0.22)
Menopausal	472	900	1.42 (0.99-2.03)	0.08 (−0.01 to 0.17)	0.26 (0.18-0.34)	0.18 (0.15-0.22)

Abbreviations: RD, risk difference; RR, risk ratio.

^a^ Male was the reference category.

^b^ Excluded individuals who were pregnant at the follow-up study visit.

### Secondary Analyses

The unadjusted proportion of female participants with long COVID was higher across all age groups: 633 of 4097 females (15%) compared with 133 of 959 males (14%) aged 18 to 39 years, 688 of 2439 females (28%) compared with 161 of 900 males (18%) aged 40 to 54 years, and 524 of 2430 females (22%) compared with 238 of 1447 males (16%) aged 55 years or older. After propensity score matching in analyses that were stratified by age, female sex was associated with a higher risk of long COVID in the subcohorts aged 40 to 54 years (RR, 1.48; 95% CI, 1.19-1.84) and 55 years or older (RR, 1.34; 95% CI, 1.11-1.61) but not in the group aged 18 to 39 years (RR, 1.04; 95% CI, 0.72-1.49) (Table 4).

Age-stratified, propensity score–matched results that accounted for menopause were similar to results in the original age-stratified analyses for the groups aged 18 to 39 years and 40 to 54 years (Table 4). The number of participants in these analyses is summarized in eFigure 3 in Supplement 1. Among participants aged 40 to 54 years, female sex was associated with higher risk of long COVID when comparing nonmenopausal females with males, with an estimated RR of 1.45 (95% CI, 1.15-1.83). When comparing menopausal females aged 40 to 54 years with males in the same age group, the risk ratio was 1.42 (95% CI, 0.99-2.03). Among participants aged 18 to 39 years, female sex was not associated with risk of long COVID among nonmenopausal female participants compared with male participants (RR, 1.10; 95% CI, 0.83-1.46).

### Sensitivity Analyses

Sensitivity analysis results are presented in eTable 1 in Supplement 1. Female sex was associated with significantly higher risk of long COVID compared with male sex when the analysis was restricted to only participants enrolled in the acute period and crossover participants (RR, 1.58; 95%, CI, 1.14-2.18). When 1755 participants (20%) who reported being pregnant between the index date and the study visit were excluded, female sex was associated with significantly higher risk of long COVID (RR, 1.50; 95%, CI, 1.27-1.77). When comorbidities were added to the propensity score model, propensity score matching still remained balanced between males and females on all covariates (eFigure 2 in Supplement 1). However, there was no longer an association between sex and long COVID (RR, 1.07; 95% CI, 0.89-1.30). After stratifying by variant era, female sex was associated with a significantly higher risk of long COVID for both Omicron and pre-Omicron cohorts (eTable 1 in Supplement 1). Female sex was still associated with higher risk of long COVID after stratifying by COVID-19 hospitalization status (eTable 1 in Supplement 1).

## Discussion

In this cohort study of the NIH RECOVER-Adult cohort, female sex was associated with a significantly higher risk of developing long COVID. Although males have more severe acute COVID-19 and higher mortality than females,^25^ in this study, females were more likely to develop long-term sequelae. The differential risk of long COVID was age, pregnancy, and menopausal status dependent.

Overall, female sex was associated with a 1.31-times higher risk of long COVID in our primary analysis full model with matching on demographic and enrollment factors, social determinants of health, and hospitalization and vaccination status during first infection. In the primary analysis reduced model (including only age, race, and ethnicity), female sex was associated with an even higher risk ratio of long COVID (1.44). This suggests that controlling for the factors that may be downstream of sex attenuated the estimated risk ratio of long COVID associated with sex. Most, but not all, published data evaluating the role of sex in long COVID have also found an elevated risk for 1 or more long COVID endotypes in female patients.^13,22,23,26,27,28,29,30,31,32,33,34,35,36,37^ When restricting the analysis to patients who were either (1) enrolled within the first 30 days of infection (subcohort with acute enrollment) or (2) enrolled initially as uninfected and were newly infected during RECOVER (crossover subcohort), thus mitigating selection bias, female sex was associated with higher risk of long COVID (RR, 1.58; 95% CI, 1.14-2.18). In age-stratified analyses, female sex was associated with the highest risk of long COVID among adults aged 40 to 54 years followed by those aged 55 years or older. A reduced RR among both females and males aged 55 years or older compared with 40 to 54 years was also found in a UK cohort, in which a sharp decline in long COVID risk was seen after the age of 70 years.^30^

In our analyses stratified by menopause status, menopausal females aged 40 to 54 years did not have significantly elevated risk of long COVID compared with males in the same age group. Several explanations are possible: there is immune activation with menopausal transition, as is seen in individuals with HIV infection^38^; female sex hormone levels decrease with age; and both higher levels of estrogen and relatively lower levels of testosterone have been associated with high risk of long COVID in nonpregnant females.^39,40,41^ Menopause and long COVID may also share some overlapping symptoms. For example, vasomotor symptoms are present in approximately 60% of newly menopausal patients.^42^ However, some symptoms of long COVID, such as palpitations, are not routinely surveyed in relation to menopause and, when described, may not be precise.^43^ Hair loss, dysomnia, sicca, and arthralgia may be present in early menopause but often are attenuated once the menopausal state is beyond the first few years. A review of the literature^17,21,44,45,46,47^ combined with our data suggests that differences in hormonal levels may partially explain the higher prevalence of long COVID in females younger than 55 years. An attenuation in the risk ratio of long COVID for females aged 18 to 39 years may be explained by most pregnant individuals belonging to this age category. In the sensitivity analysis excluding participants who were pregnant at any time between the index and study visit, female sex was still associated with an increased risk of long COVID. Sex hormones have been known to modulate immune responses through specific receptors expressed on innate immune cells and bind to promoters containing specific hormonal response elements.^21,44,45^ For example, low estradiol concentrations favor Th1-type responses (eg, interferon γ and tumor necrosis factor α) and cell-mediated immunity, whereas high estradiol concentrations, as in pregnancy, induce Th2-type responses (eg, interleukin 4 [IL-4], IL-10, and IL-13) and appear to exert effects primarily on humoral immunity.^47^ The association of immune-sensing receptors with sex-based differential susceptibility has been reported for acute COVID-19 but not for long COVID.^3,17^ Thus, sex hormones may have the potential to modulate susceptibility to and recovery from COVID-19.

Additionally, in our sensitivity analyses balanced on comorbidities, the RR for long COVID was attenuated. Many of these comorbidities (including myalgic encephalomyelitis/chronic fatigue syndrome [ME/CFS] and postural orthostatic tachycardia syndrome) are known to be associated both with female sex and with long COVID.^17,18^ Therefore, some of these comorbidities likely are mediators in the causal pathway and may partially explain the observed sex differences in the risk of long COVID.^18^ Our data align with published data on postviral ME/CFS and fibromyalgia, which are disorders that are female predominant and have known alterations in the hypothalamus-pituitary-adrenal axis.^2,28,29,45^ Multiple studies have demonstrated that chronic conditions, such as autoimmune diseases, osteoporosis, ME/CFS, and Alzheimer disease, are more prevalent in females compared with males.^48,49^ A meta-analysis focused on the long-term health problems of individuals with infection in prior coronavirus outbreaks (severe acute respiratory syndrome and Middle East respiratory syndrome) showed that females were more likely to experience a reduction in lung function, new or worsened mental health problems (eg, stress, anxiety, and depression), and low-quality life compared with males.^50^

The clinical and public health implications in terms of sex-based differences in risk of long COVID, especially based on age, pregnancy, and menopausal status, are substantial. It is important to disentangle the role of aging, hormones, inflammatory response, and comorbidities underlying these differential long COVID risk profiles and to identify which groups may benefit from specific treatments. Sex steroid–based therapies might be suggested to mitigate long COVID symptoms in females, as has already been suggested for acute COVID-19 in men.^51^ Based on this study’s findings, we believe that the sex-based disparity in long-term illness burden due to long COVID may increase in addition to existing postviral sequelae (eg, Epstein-Barr virus, ME/CFS, chronic Lyme disease, post-Ebola syndrome) that show female preponderance.

### Strengths and Limitations

Our study has several strengths. It included participants at various time points before and after SARS-CoV-2 infection, suggesting that sex differences are detectable at various time points after infection. The RECOVER cohort is larger and more socioeconomically diverse than other cohorts described in prior publications.^3,7,8,31,37^ Our estimate of RDs was balanced for demographics, variant era, vaccination status, hospitalization during acute infection, and social determinants of health. Compared with regression adjustment, propensity score–based methods permit balance on more variables without compromising statistical power^49^ and are more likely to achieve balance comparing exposed and unexposed groups.^50^ Other advantages of the RECOVER cohort include prospective data collection through a standardized questionnaire as opposed to data generated in routine clinical care, the latter of which may be subject to reporting bias and differential access. Both the RECOVER protocol and the current analysis were developed in collaboration with patient representatives.

The data used were also subject to some limitations. Bias may exist if females were more likely to report symptoms (reporting bias) or were more likely than males to enroll due to persistent long COVID symptoms (selection bias). We mitigated the latter by restricting to participants enrolled during the acute period and crossover participants only, albeit with a smaller sample size than the original cohort. We lacked direct data about sex hormone levels, timing of infection in relation to menstrual cycle, hormone-related medication use, number of pregnancies, and pregnancy-related complications. Differential dropout by sex due to symptoms may have occurred. The study lacked control data, such as long COVID–similar symptoms in patients prior to the development of COVID-19 (except for the crossover cohort), an uninfected control group, or a control group with patients infected with another virus. There is also ongoing debate regarding the utility of propensity scores; we acknowledge that the findings are sensitive to the modeling choices made when building the propensity score models.^51^ In addition, we had insufficient enrollment of participants assigned intersex at birth or who had undergone gender-affirming medical care to assess risk in those populations.

## Conclusions

This prospective NIH RECOVER-Adult cohort study found that female found that female sex was associated with an increased risk of long COVID compared with male sex and that the association was age, pregnancy, and menopausal status dependent. Understanding the mechanisms of sex differences can provide preventive and management strategies for not only long COVID but also other postviral illnesses.

## Notes

Supplement 1. **eTable 1.** Sensitivity Analyses **eTable 2.** Definition and Frequencies of Social Determinants of Health Used in Propensity Score Estimation **eFigure 1.** Distribution of Propensity Scores by Sex Assigned at Birth **eFigure 2.** Love Plot for Evaluating Covariate Balance Before and After Propensity Score Matching and Among Comorbidities for Sensitivity Analysis, Including Comorbidity Adjustment **eFigure 3.** Determination of Menopausal Status and Counts of Female Participants in Secondary Analyses, Stratified by Age Group **eFigure 4.** Symptom Frequencies, Stratified by Sex and Long COVID Subphenotype
eMethods.

Supplement 2.
RECOVER Consortium

Supplement 3.
Data Sharing Statement

## References

[1] World Health Organization. WHO COVID-19 dashboard. Accessed September 14, 2024. https://covid19.who.int/region/amro/country/us

[2] Sivan M, Taylor S. NICE guideline on long COVID. BMJ. 2020;371:m4938. doi:10.1136/bmj.m4938 33361141

[3] Demko ZO, Yu T, Mullapudi SK, . Two-year longitudinal study reveals that long COVID symptoms peak and quality of life nadirs at 6-12 months postinfection. Open Forum Infect Dis. 2024;11(3):ofae027. doi:10.1093/ofid/ofae027 38449921 PMC10917418

[4] Haslam A, Olivier T, Prasad V. The definition of long COVID used in interventional studies. Eur J Clin Invest. 2023;53(8):e13989. doi:10.1111/eci.13989 36964995

[5] Dessie ZG, Zewotir T. Mortality-related risk factors of COVID-19: a systematic review and meta-analysis of 42 studies and 423 117 patients. BMC Infect Dis. 2021;21(1):855. doi:10.1186/s12879-021-06536-3 34418980 PMC8380115

[6] Petrilli CM, Jones SA, Yang J, . Factors associated with hospital admission and critical illness among 5279 people with coronavirus disease 2019 in New York City: prospective cohort study. BMJ. 2020;369:m1966. doi:10.1136/bmj.m1966 32444366 PMC7243801

[7] Tsampasian V, Elghazaly H, Chattopadhyay R, . Risk factors associated with post-COVID-19 condition: a systematic review and meta-analysis. JAMA Intern Med. 2023;183(6):566-580. doi:10.1001/jamainternmed.2023.0750 36951832 PMC10037203

[8] Maglietta G, Diodati F, Puntoni M, . Prognostic factors for post-COVID-19 syndrome: a systematic review and meta-analysis. J Clin Med. 2022;11(6):1541. doi:10.3390/jcm11061541 35329867 PMC8948827

[9] Horwitz LI, Thaweethai T, Brosnahan SB, . Researching COVID to Enhance Recovery (RECOVER) adult study protocol: rationale, objectives, and design. PLoS One. 2023;18(6):e0286297. doi:10.1371/journal.pone.0286297 37352211 PMC10289397

[10] Thaweethai T, Jolley SE, Karlson EW, ; RECOVER Consortium. Development of a definition of postacute sequelae of SARS-CoV-2 infection. JAMA. 2023;329(22):1934-1946. doi:10.1001/jama.2023.8823 37278994 PMC10214179

[11] Geng LN, Erlandson KMFA, Hornig M, et al. 2024 Update of the RECOVER-Adult Long COVID Research Index. JAMA. Published online December 18, 2024. doi:10.1001/jama.2024.24184 PMC11862971 39693079

[12] Li F. Using propensity scores for racial disparities analysis. Obs Stud. 2023;9(1):59-68. doi:10.1353/obs.2023.0005

[13] Rosenbaum PR. A characterization of optimal designs for observational studies. J R Stat Soc B. 1991;53(3):597-610. doi:10.1111/j.2517-6161.1991.tb01848.x

[14] Stuart EA, Green KM. Using full matching to estimate causal effects in nonexperimental studies: examining the relationship between adolescent marijuana use and adult outcomes. Dev Psychol. 2008;44(2):395-406. doi:10.1037/0012-1649.44.2.395 18331131 PMC5784842

[15] Naimi AI, Whitcomb BW. Estimating risk ratios and risk differences using regression. Am J Epidemiol. 2020;189(6):508-510. doi:10.1093/aje/kwaa044 32219364

[16] Imai K, Ratkovic M. Covariate balancing propensity score. J R Stat Soc B. 2014;76:243-263. doi:10.1111/rssb.12027

[17] Stasi VD, Rastrelli G. The role of sex hormones in the disparity of COVID-19 outcomes based on gender. J Sex Med. 2021;18(12):1950-1954. doi:10.1016/j.jsxm.2021.09.003 34645593 PMC8429355

[18] Pollack B, von Saltza E, McCorkell L, . Female reproductive health impacts of long COVID and associated illnesses including ME/CFS, POTS, and connective tissue disorders: a literature review. Front Rehabil Sci. 2023;4:1122673. doi:10.3389/fresc.2023.1122673 37234076 PMC10208411

[19] Petrone D, Mateo-Urdiales A, Sacco C, ; Italian Integrated Surveillance of COVID-19. Reduction of the risk of severe COVID-19 due to Omicron compared to Delta variant in Italy (November 2021-February 2022). Int J Infect Dis. 2023;129:135-141. doi:10.1016/j.ijid.2023.01.027 36708869 PMC9877142

[20] Hyams C, Challen R, Marlow R, ; AvonCAP Research Group. Severity of Omicron (B.1.1.529) and Delta (B.1.617.2) SARS-CoV-2 infection among hospitalised adults: a prospective cohort study in Bristol, United Kingdom. Lancet Reg Health Eur. 2023;25:100556. doi:10.1016/j.lanepe.2022.100556 36530491 PMC9742675

[21] R: A Language and Environment for Statistical Computing. R Foundation for Statistical Computing; 2021.

[22] van Buuren S, Groothuis-Oudshoorn K. mice: multivariate imputation by chained equations in R. J Stat Softw. 2011;45(3):1-67.

[23] Pishgar F, Greifer N, Leyrat C, Stuart E. matchthem: matching and weighting after multiple imputation. The R Journal. 2021;13(2):292-305.

[24] Lumley T. *Survey: Analysis of Complex Survey Samples*. R package, version 4.1-2. R Project for Statistical Computing; 2023.

[25] Stall NM, Wu W, Lapointe-Shaw L, . Sex- and age-specific differences in COVID-19 testing, cases, and outcomes: a population-wide study in Ontario, Canada. J Am Geriatr Soc. 2020;68(10):2188-2191. doi:10.1111/jgs.16761 32743827

[26] Lim SH, Ju HJ, Han JH, . Autoimmune and autoinflammatory connective tissue disorders following COVID-19. JAMA Netw Open. 2023;6(10):e2336120. doi:10.1001/jamanetworkopen.2023.36120 37801317 PMC10559181

[27] Fernández-de-Las-Peñas C, Cancela-Cilleruelo I, Moro-López-Menchero P, . Exploring the trajectory curve of long-term musculoskeletal post-COVID pain symptoms in hospitalized COVID-19 survivors: a multicenter study. Pain. 2023;164(2):413-420. doi:10.1097/j.pain.0000000000002718 35930390

[28] Munblit D, Bobkova P, Spiridonova E, ; Sechenov StopCOVID Research Team. Incidence and risk factors for persistent symptoms in adults previously hospitalized for COVID-19. Clin Exp Allergy. 2021;51(9):1107-1120. doi:10.1111/cea.13997 34351016 PMC8444748

[29] Dias MB, Medeiros APV, de Melo SS, ; CO-FRAIL Study Group. The long and winding road of COVID-19 in survivors of hospitalisation: symptoms trajectory and predictors of long COVID. J Intern Med. 2023;293(2):264-268. doi:10.1111/joim.13583 36316955 PMC9878261

[30] Thompson EJ, Williams DM, Walker AJ, ; OpenSAFELY Collaborative. Long COVID burden and risk factors in 10 UK longitudinal studies and electronic health records. Nat Commun. 2022;13(1):3528. doi:10.1038/s41467-022-30836-0 35764621 PMC9240035

[31] Blomberg B, Mohn KG, Brokstad KA, ; Bergen COVID-19 Research Group. Long COVID in a prospective cohort of home-isolated patients. Nat Med. 2021;27(9):1607-1613. doi:10.1038/s41591-021-01433-3 34163090 PMC8440190

[32] Chudzik M, Babicki M, Kapusta J, . Long-COVID clinical features and risk factors: a retrospective analysis of patients from the STOP-COVID Registry of the PoLoCOV Study. Viruses. 2022;14(8):1755. doi:10.3390/v14081755 36016376 PMC9415629

[33] Baruch J, Zahra C, Cardona T, Melillo T. National long COVID impact and risk factors. Public Health. 2022;213:177-180. doi:10.1016/j.puhe.2022.09.021 36434908 PMC9683693

[34] Fernández-de-Las-Peñas C, Torres-Macho J, Elvira-Martínez CM, Molina-Trigueros LJ, Sebastián-Viana T, Hernández-Barrera V. Obesity is associated with a greater number of long-term post-COVID symptoms and poor sleep quality: a multicentre case-control study. Int J Clin Pract. 2021;75(12):e14917. doi:10.1111/ijcp.14917 34569684 PMC8646300

[35] Emecen AN, Keskin S, Turunc O, . The presence of symptoms within 6 months after COVID-19: a single-center longitudinal study. Ir J Med Sci. 2023;192(2):741-750. doi:10.1007/s11845-022-03072-0 35715663 PMC9205653

[36] Estrada-Codecido J, Chan AK, Andany N, . Prevalence and predictors of persistent post-COVID-19 symptoms. J Assoc Med Microbiol Infect Dis Can. 2022;7(3):208-219. doi:10.3138/jammi-2022-0013 36337595 PMC9629726

[37] Tleyjeh IM, Saddik B, AlSwaidan N, . Prevalence and predictors of post-acute COVID-19 syndrome (PACS) after hospital discharge: a cohort study with 4 months median follow-up. PLoS One. 2021;16(12):e0260568. doi:10.1371/journal.pone.0260568 34874962 PMC8651136

[38] Peters BA, Xue X, Sheira LA, . Menopause is associated with immune activation in women with HIV. J Infect Dis. 2022;225(2):295-305. doi:10.1093/infdis/jiab341 34174074 PMC8763955

[39] Cinislioglu AE, Cinislioglu N, Demirdogen SO, . The relationship of serum testosterone levels with the clinical course and prognosis of COVID-19 disease in male patients: a prospective study. Andrology. 2022;10(1):24-33. doi:10.1111/andr.13081 34288536 PMC8444851

[40] Moreno-Perez O, Merino E, Alfayate R, ; COVID19-ALC Research group. Male pituitary-gonadal axis dysfunction in post-acute COVID-19 syndrome—prevalence and associated factors: a Mediterranean case series. Clin Endocrinol (Oxf). 2022;96(3):353-362. doi:10.1111/cen.14537 34160836 PMC8444731

[41] Salonia A, Pontillo M, Capogrosso P, . Severely low testosterone in males with COVID-19: a case-control study. Andrology. 2021;9(4):1043-1052. doi:10.1111/andr.12993 33635589 PMC8013327

[42] Monteleone P, Mascagni G, Giannini A, Genazzani AR, Simoncini T. Symptoms of menopause—global prevalence, physiology and implications. Nat Rev Endocrinol. 2018;14(4):199-215. doi:10.1038/nrendo.2017.180 29393299

[43] Sheng Y, Carpenter JS, Elomba CD, . Review of menopausal palpitations measures. Womens Midlife Health. 2021;7(1):5. doi:10.1186/s40695-021-00063-6 34059122 PMC8167994

[44] Kadel S, Kovats S. Sex hormones regulate innate immune cells and promote sex differences in respiratory virus infection. Front Immunol. 2018;9:1653. doi:10.3389/fimmu.2018.01653 30079065 PMC6062604

[45] Vom Steeg LG, Klein SL. Sex steroids mediate bidirectional interactions between hosts and microbes. Horm Behav. 2017;88:45-51. doi:10.1016/j.yhbeh.2016.10.016 27816626 PMC6530912

[46] Hellmuth J, Barnett TA, Asken BM, . Persistent COVID-19-associated neurocognitive symptoms in non-hospitalized patients. J Neurovirol. 2021;27(1):191-195. doi:10.1007/s13365-021-00954-4 33528824 PMC7852463

[47] Grimaldi CM, Cleary J, Dagtas AS, Moussai D, Diamond B. Estrogen alters thresholds for B cell apoptosis and activation. J Clin Invest. 2002;109(12):1625-1633. doi:10.1172/JCI0214873 12070310 PMC151010

[48] Villaseca P, Cisternas P, Inestrosa NC. Menopause and development of Alzheimer’s disease: roles of neural glucose metabolism and Wnt signaling. Front Endocrinol (Lausanne). 2022;13:1021796. doi:10.3389/fendo.2022.1021796 36339406 PMC9627150

[49] Thomas N, Gurvich C, Huang K, Gooley PR, Armstrong CW. The underlying sex differences in neuroendocrine adaptations relevant to myalgic encephalomyelitis chronic fatigue syndrome. Front Neuroendocrinol. 2022;66:100995. doi:10.1016/j.yfrne.2022.100995 35421511

[50] Boden M, Cohen N, Froelich JM, Hoggatt KJ, Abdel Magid HS, Mushiana SS. Mental disorder prevalence among populations impacted by coronavirus pandemics: a multilevel meta-analytic study of COVID-19, MERS & SARS. Gen Hosp Psychiatry. 2021;70:124-133. doi:10.1016/j.genhosppsych.2021.03.006 33894561 PMC8058549

[51] Guo S, Fraser M, Chen Q. Propensity score analysis: recent debate and discussion. J Soc Social Work Res. 2020;11(3):463-482. doi:10.1086/711393

