# Errors in Supplement 2

**DOI:** 10.1001/jamanetworkopen.2025.4888

**Published:** 2025-03-13

The Original Investigation titled “Sex Differences in Long COVID,”^1^ published January 22, 2025, was corrected to fix errors in the nonauthor collaborator supplement (Supplement 2). This article was corrected online.^1^
