# Supplementary material for: Sex Differences in Long COVID
Source: JAMA Netw Open. 2025 Jan 22;8(1):e2455430. doi: 10.1001/jamanetworkopen.2024.55430 (PMC11755195; doi:10.1001/jamanetworkopen.2024.55430)
Supplement: Supplement 1. — eTable 1. Sensitivity Analyses eTable 2. Definition and Frequencies of Social Determinants of Health Used in Propensity Score Estimation eFigure 1. Distribution of Propensity Scores by Sex Assigned at Birth eFigure 2. Love Plot for Evaluating Covariate Balance Before and After Propensity Score Matching and Among Comorbidities for Sensitivity Analysis, Including Comorbidity Adjustment eFigure 3. Determination of Menopausal Status and Counts of Female Participants in Secondary Analyses, Stratified by Age Group eFigure 4. Symptom Frequencies, Stratified by Sex and Long COVID Subphenotype eMethods. [file jamanetwopen-e2455430-s001.pdf]

Supplementary Online Content

Shah DP, Thaweethai T, Karlson EW, et al; RECOVER Consortium. Sex differences in Long COVID. *JAMA Netw Open*. 2025;8(1):e2455430. doi:10.1001/jamanetworkopen.2024.55430

eTable 1. Sensitivity Analyses

eTable 2. Definition and Frequencies of Social Determinants of Health Used in Propensity Score Estimation

eFigure 1. Distribution of Propensity Scores by Sex Assigned at Birth

eFigure 2. Love Plot for Evaluating Covariate Balance Before and After Propensity Score Matching and Among Comorbidities for Sensitivity Analysis, Including Comorbidity Adjustment

eFigure 3. Determination of Menopausal Status and Counts of Female Participants in Secondary Analyses, Stratified by Age Group

eFigure 4. Symptom Frequencies, Stratified by Sex and Long COVID Subphenotype

eMethods

This supplementary material has been provided by the authors to give readers additional information about their work.

© 2025 Shah DP et al. *JAMA Network Open*.

eTable 1. Sensitivity Analyses

Analysis	Cohort	Sample Size (Female, Male)	Risk Ratio (95% CI)	Risk Difference (95% CI)	Estimated proportion with Long COVID after propensity score matching	
					Female	Male
Acute and crossover only		(3204, 1311)	1.58 (1.14, 2.18)	0.041 (0.016, 0.067)	0.114 (0.102, 0.126)	0.072 (0.050, 0.094)
Pregnant between index and study visit excluded		(7214, 3307)	1.50 (1.27, 1.77)	0.078 (0.051, 0.105)	0.233 (0.222, 0.244)	0.155 (0.131, 0.180)
Comorbidities included in propensity score model		(8969, 3307)	1.07 (0.89, 1.30)	0.013 (-0.021, 0.048)	0.195 (0.185, 0.206)	0.182 (0.149, 0.215)
Stratified by infection era	Pre-Omicron	(2991, 1024)	1.31 (1.11, 1.54)	0.084 (0.037, 0.131)	0.359 (0.339, 0.380)	0.275 (0.232, 0.319)
	Omicron	(5978, 2283)	1.37 (1.02, 1.83)	0.035 (0.006, 0.064)	0.131 (0.121, 0.140)	0.096 (0.069, 0.123)
Stratified by hospitalization status	Hospitalized	(556, 306)	1.42 (1.07, 1.87)	0.116 (0.032, 0.199)	0.393 (0.343, 0.444)	0.278 (0.205, 0.350)
	Not hospitalized	(7857, 2768)	1.23 (1.01, 1.51)	0.037 (0.004, 0.069)	0.193 (0.183, 0.203)	0.157 (0.126, 0.188)

The propensity score model with full adjustment includes all variables described in the Statistical Methods. The propensity score model with partial adjustment includes the same variables, excluding comorbidities.

© 2025 Shah DP et al. *JAMA Network Open*.

eTable 2. Definition and frequencies of social determinants of health used in propensity score estimation.

Variable	Survey Question	Response Options	Answer used to define variable	Proportion, Female [n, (%)]	Proportion, Male [n, (%)]
Married or living with partner	What is your current marital status?	• Married<br>• Divorced<br>• Widowed<br>• Separated<br>• Never Married<br>• Living with partner<br>• I prefer not to answer	• Married<br>• Living with partner	5375 (62)	2048 (64)
Homelessness	Are you currently living in transitional housing, staying in a shelter, or experiencing homelessness?	• Yes<br>• No<br>• I prefer not to answer	Yes	168 (2)	100 (3)
Disability	We would like to know about what you were doing around [infection date] -- were you working, looking for work, retired, keeping house, a student, or something else?	• Working<br>• Only temporarily laid off, sick leave or maternity leave<br>• Looking for work, unemployed<br>• Retired<br>• Disabled, permanently or temporarily.<br>• Keeping house<br>• Student<br>• Other (Specify)<br>• I prefer not to answer.<br>• I don't know	Disabled, permanently or temporarily	273 (3)	160 (5)
Unemployment			Looking for work, unemployed	218 (3)	119 (4)

© 2025 Shah DP et al. *JAMA Network Open*.

Medicaid	Are you currently covered by any of the following types of health insurance or health coverage plans? Select all that apply.	• Insurance purchased directly from an insurance company (by you or another family member)<br>• Insurance through a current or former employer or union (by you or another family member)<br>• Medicare, for people 65 or older, or people with certain disabilities<br>• Medicaid, Medical Assistance, or any kind of government assistance plan for those with low incomes or disability<br>• TRICARE, or other military health care<br>• Veteran Affairs (VA) (including those who have ever used or enrolled for	Medicaid, Medical Assistance, or any kind of government assistance plan for those with low incomes or disability	1376 (16)	407 (13)
Uninsured			I don't have health insurance, self-pay	254 (3)	117 (4)

© 2025 Shah DP et al. *JAMA Network Open*.

		• VA health care)<br>• Indian Health Service<br>• I don't have health insurance, self-pay<br>• I don't know what kind of health insurance I have<br>• I prefer not to answer			
Lost insurance due to pandemic	Did you lose health insurance coverage because of the COVID pandemic?	• Yes<br>• No<br>• Don't know.<br>• Prefer not to answer	Yes	261 (3)	102 (3)
2019 Household Income <$25,000	In 2019, what was your total household income before taxes?	• Less than $15,000<br>• $15,000 - $19,999<br>• $20,000 - $24,999<br>• $25,000 - $34,999<br>• $35,000 - $49,999<br>• $50,000 - $74,999<br>• $75,000 - $99,999<br>• $100,000 and above<br>• Prefer not to answer	• Less than $15,000<br>• $15,000 - $19,999<br>• $20,000 - $24,999	1336 (17)	453 (15)
2019 Household Income $25,000-$49,999			• $25,000 - $34,999<br>• $35,000 - $49,999	1330 (16)	411 (14)
2019 Household Income ≥$50,000			• $50,000 - $74,999<br>• $75,000 - $99,999<br>• $100,000 and above	5417 (67)	2087 (71)
Not at all difficult to	In the past month, how difficult has it	• Very difficult	Not at all difficult	5071 (61)	2007 (65)

© 2025 Shah DP et al. *JAMA Network Open*.

cover expenses	been for you to cover your expenses and pay all your bills?	• Somewhat difficult<br>• Not at all difficult<br>• Don't know<br>• Prefer not to answer			
Somewhat difficult to cover expenses			Somewhat difficult	2386 (28)	771 (25)
Very difficult to cover expenses			Very difficult	915 (11)	301 (10)
Last doctor's visit before index >5 years ago	About how long has it been since you last saw a doctor or other health professional for a wellness visit, physical, or general-purpose check-up?	• Within the previous year (less than 12 months ago)<br>• Within the previous two years (1 year but less than 2 years ago)<br>• Within the previous three years (2 years but less than 3 years ago)<br>• Within the previous five years (3 years but less than 5 years ago)<br>• Within the previous ten years (5 years but less than 10 years ago)<br>• Ten years ago or more<br>• I can't remember<br>• I prefer not to answer	• Within the previous ten years (5 years but less than 10 years ago)<br>• Ten years ago or more	41 (0)	57 (2)
Skipped medical care	During the 12 months before [infection date],	• Yes<br>• No<br>• I don't know.	Yes	505 (6)	153 (5)

© 2025 Shah DP et al. *JAMA Network Open*.

	was there any time when you needed medical care, but DID NOT GET IT because of the cost?	• I prefer not to answer			
Food insecure	Within the past 12 months before [infection date] we worried whether our food would run out before we got money to buy more.<br><br>Within the past 12 months before [infection date] the food we bought just didn't last and we didn't have money to get more.	• Often true<br>• Sometimes true<br>• Never true<br>• Prefer not to answer	If "Often true" or "Sometimes" true were selected for either question, the participant was considered food insecure	1283 (15)	457 (14)

Questions were asked at enrollment for all participants. All questions were "select one" unless otherwise specified. Proportions are calculated excluding participants with missing data, which ranged from 3.2-6.9% among male and 2.9-6.6% among female participants excluding household income data. For income data, missingness was 10.8% among males and 9.9% among females.

© 2025 Shah DP et al. *JAMA Network Open*.

eFigure 1. Distribution of propensity scores by sex assigned at birth

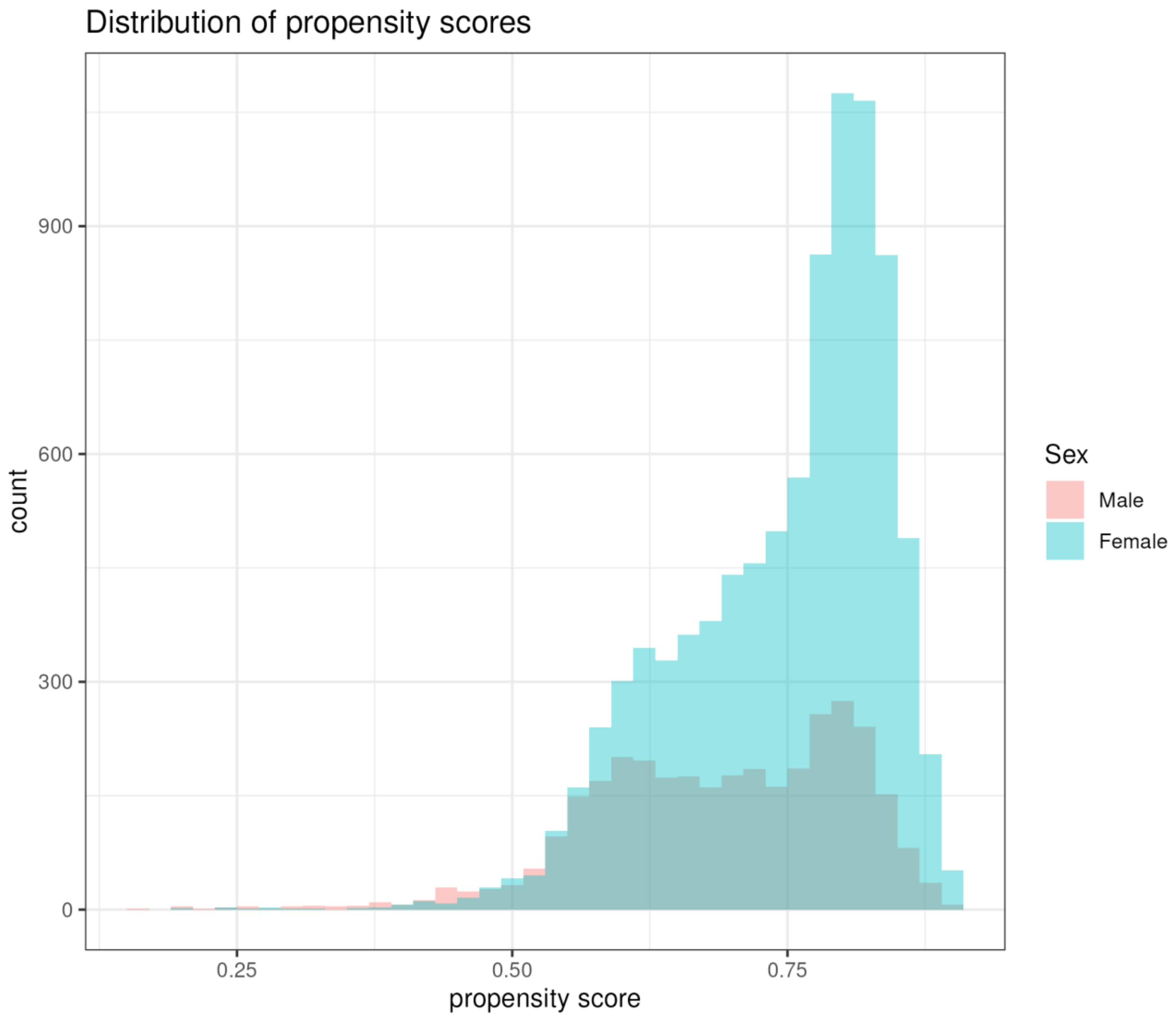

Results are shown for the fully adjusted propensity score models.

© 2025 Shah DP et al. *JAMA Network Open*.

eFigure 2A. Love plot for evaluating covariate balance before and after propensity score matching.

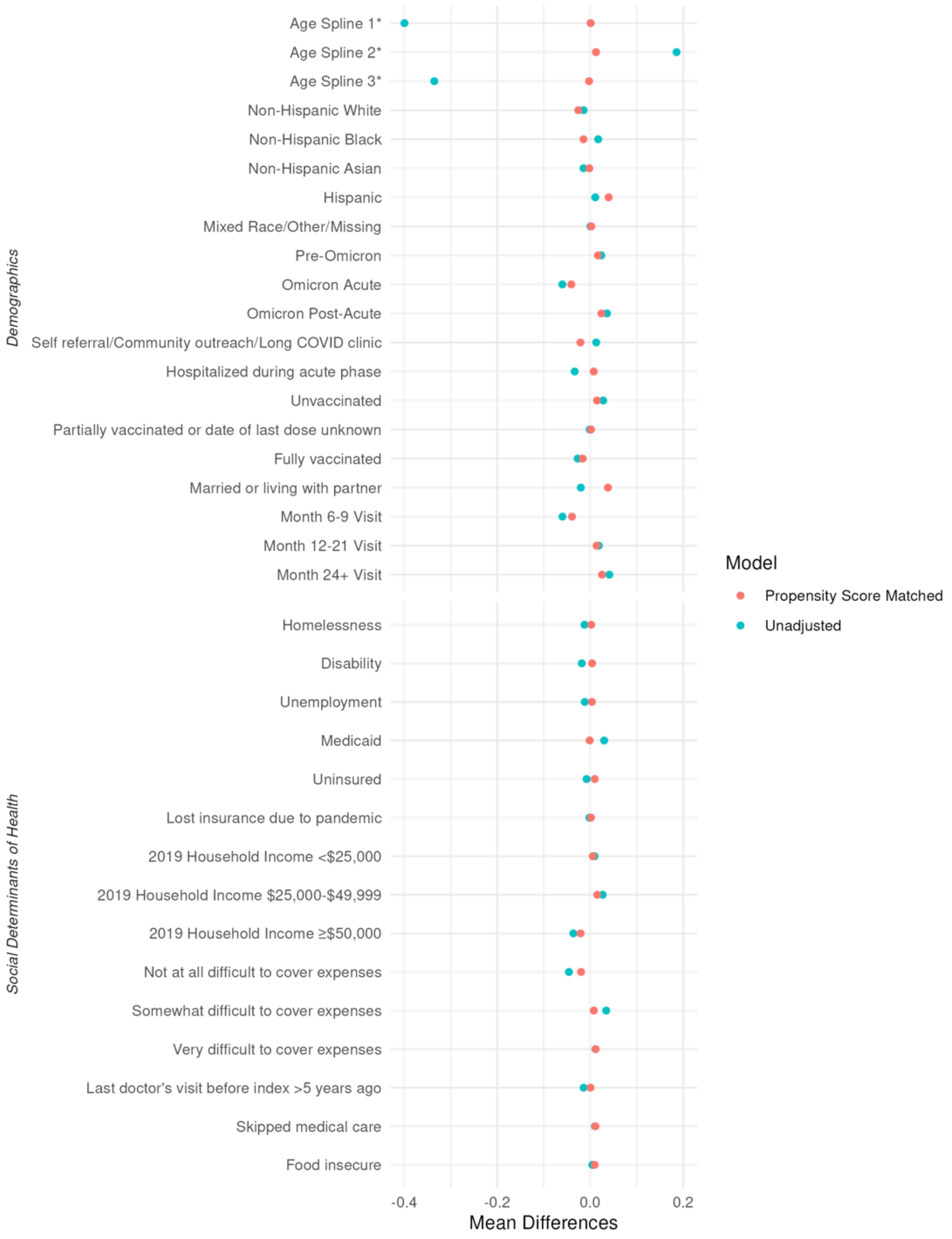

Positive mean differences indicate a higher prevalence in participants assigned female at birth, while negative values indicate a higher prevalence in those assigned male at birth. Results are averaged over all imputations.

© 2025 Shah DP et al. *JAMA Network Open*.

eFigure 2B. Love plot for evaluating covariate balance amongst comorbidities for sensitivity analysis, including comorbidity adjustment.

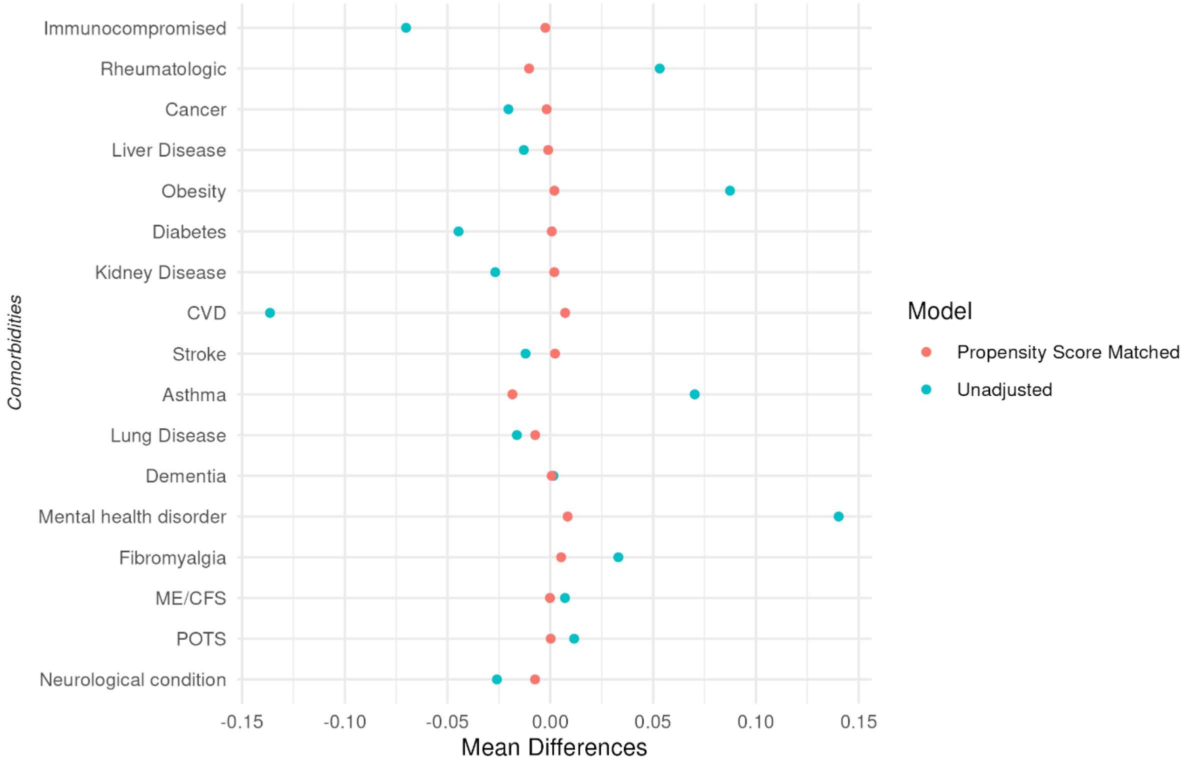

Demographic and Social Determinants of Health variables are not shown but are well balanced as in the primary model.

© 2025 Shah DP et al. *JAMA Network Open*.

eFigure 3. Determination of menopausal status and counts of female participants in secondary analyses, stratified by age group.

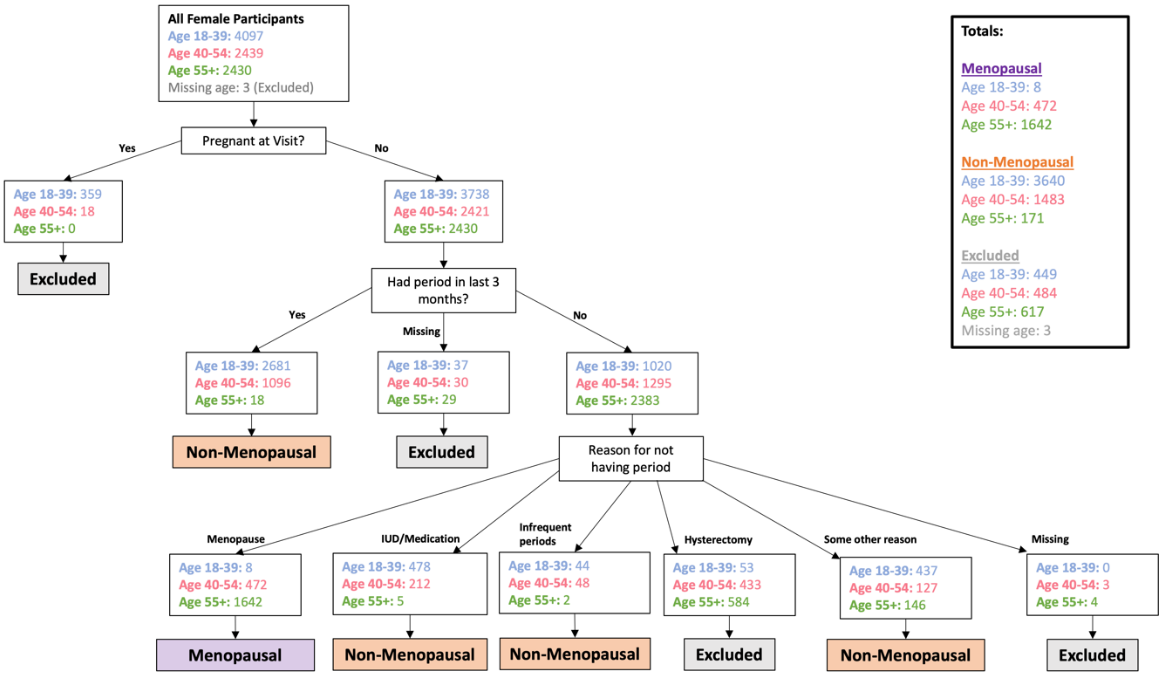

© 2025 Shah DP et al. *JAMA Network Open*.

eFigure 4. Symptom frequencies, stratified by sex and Long COVID sub-phenotype

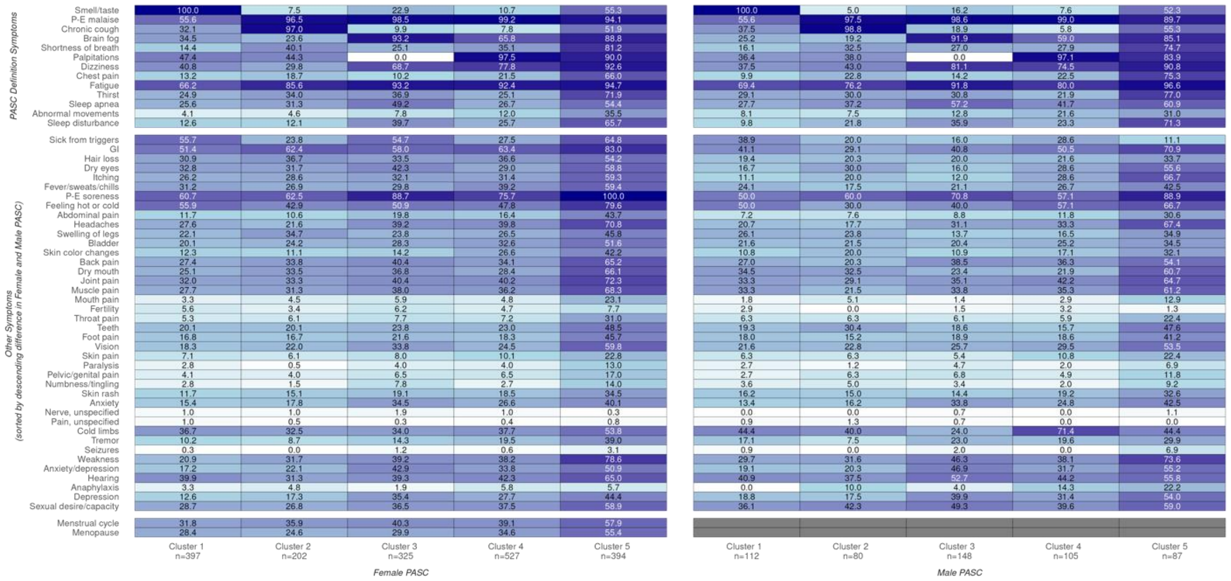

Symptoms are ordered by contribution to Long COVID Research Index and then the difference in female Long COVID frequency and male Long COVID frequency overall (not sub-phenotype-specific), to match the order in Table 2.

© 2025 Shah DP et al. *JAMA Network Open*.

eMethods

Details of variables used in propensity score matching:

Demographic and enrollment variables

Age was defined as the age in years of the participant at the time of first infection. Race and ethnicity was self-reported using the following survey instrument:

Participants were asked: "Which of these categories describe you (select all that apply)?"

1. American Indian or Alaska Native
   (For example: Aztec, Blackfeet Tribe, Mayan, Navajo Nation, Native Village of Barrow (Utqiagvik) Inupiat Traditional Government, Nome Eskimo Community, etc.)
2. Asian
   (For example: Asian Indian, Chinese, Filipino, Japanese, Korean, Vietnamese, etc.)
3. Black or African American
   (For example: African American, Ethiopian, Haitian, Jamaican, Nigerian, Somali, etc.)
4. Hispanic, Latino, or Spanish
   (For example: Colombian, Cuban, Dominican, Mexican or Mexican American, Puerto Rican, Salvadoran, etc.)
5. Middle Eastern or North African
   (For example: Algerian, Egyptian, Iranian, Lebanese, Moroccan, Syrian, etc.)
6. Native Hawaiian or other Pacific Islander
   (For example: Chamorro, Fijian, Marshallese, Native Hawaiian, Tongan, etc.)
7. White
   (For example: English, European, French, German, Irish, Italian, Polish, etc.)
8. None of these fully describe me
9. Prefer not to answer

Participants were then categorized using the following logic (listed in order of priority):

1. Participants who selected #4, whether or not other responses were selected, were categorized as "Hispanic, Latino, or Spanish"
2. Participants who selected #3 only were categorized as "Black or African-American, non-Hispanic"
3. Participants who selected #2 only were categorized as "Asian, non-Hispanic"
4. Participants who selected #5 and/or #7 only were categorized as "White, non-Hispanic"
5. Participants who did not answer this question or selected any other combination of responses were categorized as "Mixed Race/Other/Missing"

Acute participants were those who enrolled within 30 days of infection, while post-acute participants were enrolled any time after. Crossover participants were grouped together with acute participants because, like acute participants, our ability to study outcomes related to crossover infections are not subject to potential selection bias into the study because of long-term symptoms. Participants were divided into three groups as defined by their timing of enrollment: Pre-Omicron, Omicron Acute, and Omicron Post-Acute. Pre-Omicron is defined as an infection before 12/1/2021, while Post-Omicron is 12/1/2021 or later. The Pre-Omicron group was not separated into acute/post-acute because of minimal acute enrollment before 12/1/2021 (17 of 3248 (0.5%) Pre-Omicron were acute).

Participants were categorized into one of the following enrollment groups:

© 2025 Shah DP et al. *JAMA Network Open*.

1. Community outreach
2. Public health department list
3. Community health center
4. Participant tested/treated in the health system
5. Existing, prospectively-followed COVID cohort
6. Existing non-COVID research or clinical cohort
7. Long COVID clinic
8. Self-referral from the RECOVER website or other unsolicited self-referral

Our referral status variable categorized participants as “Self-referral/Community outreach/Long COVID clinic” if (1), (7), or (8) was selected and “Other referral” another answer was selected.

Comorbidities

Comorbidities included conditions reported before first SARS-CoV-2 infection, or, for crossover participants, conditions reported up to but not including the visit at which the on-study infection was reported. At enrollment, participants are asked, “Have you been diagnosed with any of the following conditions?” Participants who respond “Yes, already had this condition during the year before my infection” are categorized as having the comorbidity. Crossover participants are asked "Have you been diagnosed with any of the following conditions in the time since your last visit?” Crossover participants may also have been asked the enrollment and follow-up version (“Have you been diagnosed with any of the following conditions in the last three months?”) of the questions at visits before their first infection. Crossover participants who respond “Yes” or “Yes, already had this condition during the year before my infection” to any of these questions before infection are categorized as having the comorbidity. Some participants without the initial form also received an updated version of the comorbidities form. The updated version asked for comorbidities and diagnosis date, which were mapped back to the initial questions amongst those with missing data on the initial form.

The following comorbidity names were used as shorthand for how they appear in the survey:

1. Immunocompromised condition [Immunocompromised condition (such as a transplant, HIV, or an immune deficiency]
2. Cardiovascular disease [Cardiovascular disease (e.g., heart failure, heart attack, high blood pressure); High blood pressure, with or without treatment (hypertension, HTN)]
3. Stroke [Stroke, TIA (transient ischemic attack or mini-stroke), intracerebral hemorrhage or subarachnoid hemorrhage (bleeding in the brain), or cerebral venous thrombosis (type of blood clot in the brain)]
4. Lung disease [Chronic obstructive pulmonary disease (COPD) including emphysema, chronic bronchitis, obstructive pulmonary disease; other chronic lung disease]
5. Dementia [Dementia, memory impairment, cognitive disorder, or developmental delay],
6. Mental health disorder [Depression or anxiety disorder; Bipolar disorder or psychosis (hearing or seeing things others can't; odd or unusual beliefs; paranoia); other mental health disorder]
7. ME/CFS [Myalgic encephalomyelitis/chronic fatigue syndrome (ME/CFS)],

© 2025 Shah DP et al. *JAMA Network Open*.

8. POTS [POTS (Postural Orthostatic Tachycardia Syndrome) or other form of dysautonomia or autonomic dysfunction]
9. Neurological condition [Seizure disorder; Neuromuscular disease (neuropathy, myopathy, myasthenia gravis, etc.); movement disorder]

Vaccination Status

The initial definition of vaccination status is detailed in [doi:10.1001/jama.2023.8823 Supplement 3, eTable 4]. The same definition was applied except that the first infection date was used instead of the index date, and “Partially vaccinated” and “Date of last dose unknown” were combined into one category.

Menopause

Female participants were asked at each study visit “Have you had a period in the last 3 months?” and could select “Yes” or “No”. If a participant selects “No”, they are then asked “Why have you not had a period in the last 3 months?” with the answer choices:

1. I am in menopause
2. I had a hysterectomy
3. I am pregnant
4. I am taking a medication or using an IUD that stops my period
5. My periods come infrequently
6. Some other reason

Participants could only choose one answer.

A participant is defined as menopausal if they indicated that they had not had a period in the last 3 months and selected answer choice (1) from the follow-up question above.

A participant is defined as non-menopausal if they indicated that they had a period in the last 3 months or if they have not had a period in the last 3 months but select answer choices (4), (5), or (6).

Participants were excluded from secondary analyses related to menopause if:

- They were pregnant at the visit (selected choice (3) or indicated an active pregnancy on the pregnancy or pregnancy follow-up form),
- They indicated that they had not had a period in the last 3 months and selected answer choice (2) ,
- They did not respond to the question regarding menstruation, or
- They did respond to the question regarding menstruation saying “No” but did not answer the follow-up question.

See Supplemental Figure 3 for numbers of participants who were categorized as menopausal, non-menopausal, or excluded from these analyses. Because almost all female participants aged 55+ were menopausal, we did not stratify by menopause status in this age group.

eMethods References

doi:10.1001/jama.2023.8823

© 2025 Shah DP et al. *JAMA Network Open*.
