# Supplementary material for: Sex Differences in Long COVID
Source: JAMA Netw Open. 2025 Jan 22;8(1):e2455430. doi: 10.1001/jamanetworkopen.2024.55430 (PMC11755195; doi:10.1001/jamanetworkopen.2024.55430)
Supplement: Supplement 2. — RECOVER Consortium [file jamanetwopen-e2455430-s002.pdf]

Supplemental Online Content: Nonauthor Collaborators
*First name, last name, and suffix (if applicable) are required and will appear in PubMed.

*Group Name(s):							
***First Name and Middle Initial(s)**	***Last Name**	***Suffix (eg, Jr, III)**	Academic Degrees	Institution	Location (city, state/province, country)	Role or Contribution, eg, chair, principal investigator	Group (if more than 1 Group listed in the byline) and/or Subgroup (eg, Steering Committee)
Jenny E.	Han		MD, MS	Emory University School of Medicine	Atlanta, GA, USA	SubSite PI	RECOVER-Adult
Vincent C.	Marconi		MD	Emory University School of Medicine and Rollins School of Public Health	Atlanta, GA, USA	SubSite PI, PIPP core member	RECOVER-Adult
Ighovwerha	Ofotokun		MD, MSc	Emory University	Atlanta, GA, USA	PD/PI	RECOVER-Adult
Rachel E.	Patzer		PhD, MPH	Emory University School of Medicine	Atlanta, GA, USA	MPI	RECOVER-Adult
Tiffany A.	Walker		MD	Emory University School of Medicine	Atlanta, GA, USA	SubSite PI, Adjudication Co-Chair	RECOVER-Adult
Rachael	Abraham		PhD, MSc	Emory University	Atlanta, GA, USA	Regulatory Manager	RECOVER-Adult
Franchesca A.	Aguilar			Emory University	Atlanta, GA, USA	GRA/ Data Analyst	RECOVER-Adult
Ghazal	Ahmadi-Izad						RECOVER-Adult
Dilshad R.	Ahmed						RECOVER-Adult
Alicarmen	Alvarez						RECOVER-Adult
Blake	Anderson		MD	Emory University	Atlanta, GA, USA		RECOVER-Adult
Walter D.	Asencios		BS	Emory University School of Medicine	Atlanta, GA, USA	Clinical Research Coordinator	RECOVER-Adult
Casey L.	Beaty		MS	Emory University	Atlanta, GA, USA	Regulatory Specialist	RECOVER-Adult
Brahmchetna	Bedi		PhD	Emory University	Atlanta, GA, USA	Lab Manager	RECOVER-Adult
Jasmine A.	Berry		MPH	Emory University	Atlanta, GA, USA	Epidemiologist	RECOVER-Adult
Donchel	Boone						RECOVER-Adult
Mary	Bower		BSN				RECOVER-Adult
James D.	Bremner		MD	Emory University	Atlanta, GA, USA	Co-Investigator	RECOVER-Adult
Corbin	Brent						RECOVER-Adult

Supplemental Online Content: Nonauthor Collaborators
*First name, last name, and suffix (if applicable) are required and will appear in PubMed.

***First Name and Middle Initial(s)**	***Last Name**	***Suffix (eg, Jr, III)**	Academic Degrees	Institution	Location (city, state/province, country)	Role or Contribution, eg, chair, principal investigator	Group (if more than 1 Group listed in the byline) and/or Subgroup (eg, Steering Committee)
Ke'Ara	Brown-Smith		MS	Emory University School of Medicine	Atlanta, GA, USA	Clinical Research Coordinator	RECOVER-Adult
Rachel	Bull						RECOVER-Adult
Gustavo	Capo						RECOVER-Adult
Kelechi	Carl-Igwe						RECOVER-Adult
Calista	Chitadze						RECOVER-Adult
Nachi	Chukwumerije			Emory University	Atlanta, GA, USA	Assistant Program Cordinator	RECOVER-Adult
Erna	Clyburn						RECOVER-Adult
Shelby	Collins		NP	Emory University	Atlanta, GA, USA	Clinical Director	RECOVER-Adult
Julie	Costello						RECOVER-Adult
Grace	Couture						RECOVER-Adult
Angel	Craft		MBA	Emory University	Atlanta, GA, USA	Lab Technician	RECOVER-Adult
Xiangqin	Cui						RECOVER-Adult
Carlos	del Rio		MD	Emory University School of Medicine	Atlanta, GA, USA	Co-Investigator	RECOVER-Adult
Joshua F.	Detelich		MD, MSc	Emory University School of Medicine	Atlanta, GA, USA	Co-Investigator	RECOVER-Adult
Cartia	Dixon						RECOVER-Adult
Jeanne	Dow						RECOVER-Adult
D'Andrea	Doyle		MSPH	Emory University	Atlanta, GA, GA	Communications Specialist	RECOVER-Adult
Jannah	Elchommali		BS	Emory University School of Medicine	Atlanta, GA, USA	Clinical Research Coordinator	RECOVER-Adult
Imani	Elsey		BS	Emory University School of Medicine	Atlanta, GA, USA	Clinical Research Coordinator	RECOVER-Adult
Nicole	Franks		MD	Emory University School of Medicine	Atlanta, GA, USA	Co-Investigator	RECOVER-Adult
Julia	Gallini						RECOVER-Adult
Evan	Gutter		MPH				RECOVER-Adult
Jess	Harding						RECOVER-Adult

Supplemental Online Content: Nonauthor Collaborators
*First name, last name, and suffix (if applicable) are required and will appear in PubMed.

***First Name and Middle Initial(s)**	***Last Name**	***Suffix (eg, Jr, III)**	Academic Degrees	Institution	Location (city, state/province, country)	Role or Contribution, eg, chair, principal investigator	Group (if more than 1 Group listed in the byline) and/or Subgroup (eg, Steering Committee)
Liliana	Hernandez						RECOVER-Adult
Carla	Holloway						RECOVER-Adult
Cynthia	Ifejika		MPH	Emory University School of Medicine	Atlanta, GA, USA	Clinical Research Coordinator	RECOVER-Adult
Rijalda	Jasarevic		MPH				RECOVER-Adult
Vidhi N.	Javia		BS	Emory University School of Medicine	Atlanta, GA, USA	Clinical Research Coordinator	RECOVER-Adult
Mykayla	Jeter						RECOVER-Adult
Yasha	Joseph			Emory University	Atlanta, GA, USA	Undergraguate Research Assistanta	RECOVER-Adult
Monica	Juarez						RECOVER-Adult
Caitlin M.	Kirkpatrick			Emory University	Atlanta, GA, USA	Lab Technician	RECOVER-Adult
Athena	Koumanelis						RECOVER-Adult
Shilpa	Krishnan		PT, PhD	Emory University	Atlanta, GA, USA	Physical Therapist; Collaborator	RECOVER-Adult
Jose D.	Leon			Emory University	Atlanta, GA, USA	Lab Technician	RECOVER-Adult
Valerie	Lew		NP	Emory University	Atlanta, GA, USA	Research Nurse Practitioner Lead	RECOVER-Adult
Cheryl L.	Maier		MD, PhD	Emory University School of Medicine	Atlanta, GA, USA	Co-Investigator	RECOVER-Adult
Nour	Makkaoui		MD				RECOVER-Adult
Mara	Maroney			Emory University	Atlanta, GA, USA	Regulatory Specialist	RECOVER-Adult
Christopher F.	Martin		MBA	Emory University	Atlanta, GA, USA	Project Director, Atlanta Hub	RECOVER-Adult
Loice	Mbogo						RECOVER-Adult
Atuarra	McCaslin						RECOVER-Adult
Jerrod	McIntyre						RECOVER-Adult
Abeer	Moanna			Emory University School of Medicine			RECOVER-Adult

Supplemental Online Content: Nonauthor Collaborators
*First name, last name, and suffix (if applicable) are required and will appear in PubMed.

***First Name and Middle Initial(s)**	***Last Name**	***Suffix (eg, Jr, III)**	Academic Degrees	Institution	Location (city, state/province, country)	Role or Contribution, eg, chair, principal investigator	Group (if more than 1 Group listed in the byline) and/or Subgroup (eg, Steering Committee)
Miranda	Montoya						RECOVER-Adult
Elena	Morales						RECOVER-Adult
Caitlin A.	Moran		MD, MSc	Emory University School of Medicine	Atlanta, GA, USA	Co-Investigator	RECOVER-Adult
Calista	Murray						RECOVER-Adult
Roslin	Nelson						RECOVER-Adult
Tran	Nguyen		MS	Emory University	Atlanta, GA, USA	Lab Technician	RECOVER-Adult
Bukkie	Ojoawo						RECOVER-Adult
Eileen	Osinski						RECOVER-Adult
Sofia	Oviedo						RECOVER-Adult
Yolanda	Paredes-Gaitan		PHD, MPH, MOH	Emory University School of Medicine	Atlanta, GA, USA	Clinical Reserach Coordinator	RECOVER-Adult
Michael	Prude						RECOVER-Adult
Grace	Ramakrishnan						RECOVER-Adult
Paulina A.	Rebolledo		MD	Emory University			RECOVER-Adult
Marjorie	Roberts				Atlanta, GA, USA	Patient Representative	RECOVER-Adult
Keysha	Robinson						RECOVER-Adult
Chantrice	Rogers						RECOVER-Adult
Nadine G.	Rouphael		MD	Emory University	Decatur, GA, USA	Co-Investigator	RECOVER-Adult
Charles	Searles		MD, MS				RECOVER-Adult
Marni	Segall						RECOVER-Adult
Anand	Shah		MD				RECOVER-Adult
Ruvina	Silva						RECOVER-Adult
Cheryl	Simpson						RECOVER-Adult
Krystal	Simpson-Derrell						RECOVER-Adult
Talib	Sirajud-Deen						RECOVER-Adult
Jacob	Stroud						RECOVER-Adult

Supplemental Online Content: Nonauthor Collaborators
*First name, last name, and suffix (if applicable) are required and will appear in PubMed.

***First Name and Middle Initial(s)**	***Last Name**	***Suffix (eg, Jr, III)**	Academic Degrees	Institution	Location (city, state/province, country)	Role or Contribution, eg, chair, principal investigator	Group (if more than 1 Group listed in the byline) and/or Subgroup (eg, Steering Committee)
Mehul S.	Suthar		PhD	Emory Vaccine Center; Emory University School of Medicine	Atlanta, GA, USA	Vice chair of Microbiology Committee	RECOVER-Adult
Cory	Sylber			Emory University	Atlanta, GA, USA	Laboratory Technician	RECOVER-Adult
Ashley	Sylvera						RECOVER-Adult
Larissa J.	Teunis		MPA	Emory University	Atlanta, GA, USA	Co-Project Director, Atlanta Hub	RECOVER-Adult
Kodasha M.	Thomas						RECOVER-Adult
Kehmia	Titanji		PhD	Emory University	Atlanta, GA, USA	Laboratory	RECOVER-Adult
Christopher	Toy						RECOVER-Adult
Alex	Truong						RECOVER-Adult
Viola	Vaccarino		MD, PhD	Emory University School of Public Health	Atlanta, GA, USA	Co-Investigator	RECOVER-Adult
Kris	Varney			Emory University	Atlanta, GA, USA	Research Administrative Coordinator	RECOVER-Adult
Kartavya	Vyas						RECOVER-Adult
Kurt	Vyas						RECOVER-Adult
Max	Walkow						RECOVER-Adult
Tamara	Wesley						RECOVER-Adult
Juton R.	Winston		BS	Emory University School of Medicine	Atlanta, GA, USA	Clinical Reserach Coordinator	RECOVER-Adult
Terra J.	Winter						RECOVER-Adult
Cherry	Wongtrakool		MD	Emory University School of Medicine	Atlanta, GA, USA	Collaborator	RECOVER-Adult
Sushma K.	Cribbs		MD, MSc	Emory University School of Medicine	Atlanta, GA, USA	SubSite PI	RECOVER-Adult
Anyssa G.	Francis		BA, MA	Atlanta VA Medical Center	Atlanta, GA, USA	Clinical Research Coordinator	RECOVER-Adult

Supplemental Online Content: Nonauthor Collaborators
*First name, last name, and suffix (if applicable) are required and will appear in PubMed.

***First Name and Middle Initial(s)**	***Last Name**	***Suffix (eg, Jr, III)**	Academic Degrees	Institution	Location (city, state/province, country)	Role or Contribution, eg, chair, principal investigator	Group (if more than 1 Group listed in the byline) and/or Subgroup (eg, Steering Committee)
Tina P.	Hang		MD	Atlanta VA Medical Center	Decatur, GA,	Atlanta Veterans Affairs Medical Center	RECOVER-Adult
Ketteria D.	Ingram		BS	Atlanta VA Medical Center			RECOVER-Adult
Jordi	Lainez		BS	Atlanta VA Medical Center	Atlanta, GA, USA	Clinical Research Coordinator	RECOVER-Adult
Zanthia	Wiley		MD	Emory University School of Medicine	Atlanta, GA, USA	MPI	RECOVER-Adult
Arijan	Ager		MPH	Emory Hope Clinic			RECOVER-Adult
Mary	Atha		ACNP-BC	Emory Hope Clinic	Decatur, GA, USA	Sub-Investigator	RECOVER-Adult
Natalie	Gray			Emory Hope Clinic			RECOVER-Adult
Ash	Grimes			Emory Hope Clinic			RECOVER-Adult
Lauren N.	Hewitt		LPN	Emory Hope Clinic			RECOVER-Adult
Christopher	Huerta			Emory Hope Clinic			RECOVER-Adult
Brandi	Johnson		BS	Emory Hope Clinic	Decatur, GA, USA	Laboratory	RECOVER-Adult
Lana	Khalil		MD	Emory Hope Clinic	Decatur, GA, USA	Clinical Research Coordinator	RECOVER-Adult
Dean	Kleinhenz			Emory Hope Clinic	Decatur, GA, USA	Administrator	RECOVER-Adult
Alexandra	Koumanelis			Emory Hope Clinic	Decatur, GA, USA		RECOVER-Adult
Rebecca	Kozoman		BS	Emory Hope Clinic	Decatur, GA, USA	Clinical Research Coordinator	RECOVER-Adult
Matthew A.	Lee		MS	Emory Hope Clinic	Atlanta, GA, USA	Clinical Research Coordinator	RECOVER-Adult
Kennedy C.	Lewis		MPH	Emory Hope Clinic	Decatur, GA, USA	Clinical Research Coordinator	RECOVER-Adult
Matthew	Litvack			Emory Hope Clinic	Decatur, GA, USA	Clinical Research Coordinator	RECOVER-Adult
Tsungirirai	Maramba		MPH	Emory Hope Clinic	Decatur, GA, USA	Biostatistician	RECOVER-Adult
Christina	Mehta		PhD	Emory Hope Clinic	Decatur, GA, USA	Biostatistician, NBR PI	RECOVER-Adult
Bernadine	Panganiban			Emory Hope Clinic			RECOVER-Adult

*First name, last name, and suffix (if applicable) are required and will appear in PubMed.

***First Name and Middle Initial(s)**	***Last Name**	***Suffix (eg, Jr, III)**	Academic Degrees	Institution	Location (city, state/province, country)	Role or Contribution, eg, chair, principal investigator	Group (if more than 1 Group listed in the byline) and/or Subgroup (eg, Steering Committee)
Kazi	Rahman			Emory Hope Clinic			RECOVER-Adult
Veronica E.	Smith		MSN	Emory Hope Clinic	Decatur, GA, USA	Research Nurse Practitioner	RECOVER-Adult
Andre	Stringer			Emory Hope Clinic			RECOVER-Adult
Maliya	Tolbert			Emory Hope Clinic			RECOVER-Adult
Jessica	Traenkner		PA, MPAS	Emory Hope Clinic	Decatur, GA, USA	Co-Investigator	RECOVER-Adult
Kristen	Unterberger		MMSc, PA-C	Emory Hope Clinic	Atlanta, GA, USA	Co-Investigator	RECOVER-Adult
Heqiong	Wang		MPH	Emory Hope Clinic	Decatur, GA, USA	Biostatistician	RECOVER-Adult
Erika	Wimberly			Emory Hope Clinic			RECOVER-Adult
Qian	Yang		PhD	Emory Hope Clinic	Decatur, GA, USA	Biostatistician	RECOVER-Adult
Jennifer C.	Gander		PhD	Kaiser Permanente of Georgia	Atlanta, GA, USA	SubSite PI	RECOVER-Adult
Robert B.	Neuman		MD	Kaiser Permanente of Georgia	Atlanta, GA, USA	SubSite PI, Co-Investigator	RECOVER-Adult
Patricia A.	Bush		MS, EdD	Kaiser Foundation Health Plan of GA Inc	Atlanta, GA, USA	Project Manager, KPGA	RECOVER-Adult
Alex F.	Hudgins			Kaiser Permanente of Georgia			RECOVER-Adult
Imanii	Kolailat			Kaiser Permanente of Georgia	Atlanta, GA,		RECOVER-Adult
Monica	Martinez			Kaiser Permanente of Georgia			RECOVER-Adult
Sierra G.	Thompson			Kaiser Permanente of Georgia			RECOVER-Adult
Chiagoziem	Agu		MD	Morehouse School of Medicine	Atlanta, GA, USA	Sub-PI	RECOVER-Adult
Priscilla	Pemu		MD, MS	Morehouse School of Medicine	Atlanta, GA, USA	MPI	RECOVER-Adult
Amir	Afani		BS	Morehouse School of Medicine	Atlanta, GA, USA	Research Coordinator	RECOVER-Adult
Concilia	Ariri		BS	Morehouse School of Medicine	Atlanta, GA, USA	Research Coordinator	RECOVER-Adult
Annette	Dandy			Morehouse School of Medicine	Atlanta, GA, USA		RECOVER-Adult
Kaysha	Harper		BS	Morehouse School of Medicine	Atlanta, GA, USA	Research Coordinator	RECOVER-Adult

Supplemental Online Content: Nonauthor Collaborators
*First name, last name, and suffix (if applicable) are required and will appear in PubMed.

*First Name and Middle Initial(s)	*Last Name	*Suffix (eg, Jr, III)	Academic Degrees	Institution	Location (city, state/province, country)	Role or Contribution, eg, chair, principal investigator	Group (if more than 1 Group listed in the byline) and/or Subgroup (eg, Steering Committee)
Carmel	Ibeawuchi		MBA	Morehouse School of Medicine	Atlanta, GA, USA	Clinical Research Coordinator	RECOVER-Adult
Brianna	Lawrence			Morehouse School of Medicine			RECOVER-Adult
Jan	Morgan-Billingslea			Morehouse School of Medicine			RECOVER-Adult
Elizabeth I.	Ojemakinde		MD, MPH	Morehouse School of Medicine	Atlanta, GA, USA	Project Director, MSM	RECOVER-Adult
Karina	Smith		BS	Morehouse School of Medicine	Atlanta, GA, USA	Research Coordinator	RECOVER-Adult
Bruce D.	Levy		MD	Brigham and Women's Hospital, Harvard Medical School	Boston, MA, USA	Hub PI, SubSite PI	RECOVER-Adult
Masanori	Aikawa		MD, PhD	Brigham and Women's Hospital, Harvard Medical School	Boston, MA, USA	Co-Investigator	RECOVER-Adult
Lindsey	Baden			Brigham and Women's Hospital, Harvard Medical School	Boston, MA, USA	Co-Investigator	RECOVER-Adult
Gaston	Baslet		MD	Brigham and Women's Hospital, Harvard Medical School	Boston, MA, USA	Co-Investigator	RECOVER-Adult
Lindsey	Bennett		BA	Brigham and Women's Hospital	Boston, MA, USA	Clinical Research Coordinator	RECOVER-Adult
Shamik	Bhattacharyya		MD, MS	Brigham and Women's Hospital	Boston, MA, USA	Co-Investigator	RECOVER-Adult
Julie	Buring		SCD, MS, BA	Brigham and Women's Hospital, Harvard Medical School	Boston, MA, USA	Co-Investigator	RECOVER-Adult
Rebecca E.	Cagnina		MD, PhD	Brigham and Women's Hospital	Boston, MA, USA	Co-Investigator	RECOVER-Adult
Li Qing	Chen		BA	Brigham and Women's Hospital	Boston, MA, USA	Project Director	RECOVER-Adult
Cheryl R.	Clark		MD, ScD	Brigham and Women's Hospital	Boston, MA, USA	Co-Investigator	RECOVER-Adult
Pieter	Cohen						RECOVER-Adult
Charles	Czeisler		MD, Ph.D	Brigham and Women's Hospital, Harvard Medical School	Boston, MA, USA	Co-Investigator	RECOVER-Adult
Peter	Estill		BA	Brigham and Women's Hospital	Boston, MA, USA	Clinical Research Coordinator	RECOVER-Adult

*First name, last name, and suffix (if applicable) are required and will appear in PubMed.

***First Name and Middle Initial(s)**	***Last Name**	***Suffix (eg, Jr, III)**	Academic Degrees	Institution	Location (city, state/province, country)	Role or Contribution, eg, chair, principal investigator	Group (if more than 1 Group listed in the byline) and/or Subgroup (eg, Steering Committee)
Elizabeth	Gay			Brigham and Women's Hospital, Harvard Medical School	Boston, MA, USA	Co-Investigator	RECOVER-Adult
Jessica	Hong		BS	Brigham and Women's Hospital	Boston, MA, USA	Research Assistant	RECOVER-Adult
Daniela	Lamas		MD	Brigham and Women's Hospital	Boston, MA, USA	Co-Investigator	RECOVER-Adult
Sarina	Lay		BS	Brigham and Women's Hospital	Boston, MA, USA	Research Coordinator	RECOVER-Adult
Nomi	Levy-Carrick			Brigham and Women's Hospital	Boston, MA, USA	Co-Investigator	RECOVER-Adult
JoAnn	Manson			Brigham and Women's Hospital	Boston, MA, USA	Co-Investigator	RECOVER-Adult
Tobasom	Monafrared			Brigham and Women's Hospital	Boston, MA, USA	Research Coordinator	RECOVER-Adult
Susan	Redline		MD, MPH	Brigham and Women's Hospital, Harvard Medical School	Boston, MA, USA	Co-Investigator	RECOVER-Adult
Elijah J.	Remis		BA	Brigham and Women's Hospital	Boston, MA, USA	Clinical Research Coordinator	RECOVER-Adult
Daniel	Schilkrut			Brigham and Women's Hospital	Boston, MA, USA	Research Coordinator	RECOVER-Adult
Howard D.	Sesso		ScD, MPH	Brigham and Women's Hospital	Boston, MA, USA	Co-Investigator	RECOVER-Adult
Scott	Solomon			Brigham and Women's Hospital, Harvard Medical School	Boston, MA, USA	Co-Investigator	RECOVER-Adult
Jeffrey A.	Sparks		MD, MSc	Brigham and Women's Hospital	Boston, MA, USA	Co-Investigator	RECOVER-Adult
Lia L.	Spencer		BS	Brigham and Women's Hospital	Boston, MA, USA	Clinical Research Coordinator	RECOVER-Adult
David	Systrom		MD	Brigham and Women's Hospital, Harvard Medical School	Boston, MA, USA	Co-Investigator	RECOVER-Adult
Phyo Phyo Min	Thu		BS	Brigham and Women's Hospital	Boston, MA, USA	Clinical Research Coordinator	RECOVER-Adult
David	Walt		PHD	Brigham and Women's Hospital, Harvard Medical School	Boston, MA, USA	Co-Investigator	RECOVER-Adult
George	Washko		MD	Brigham and Women's Hospital, Harvard Medical School	Boston, MA, USA	Co-Investigator	RECOVER-Adult

***First Name and Middle Initial(s)**	***Last Name**	***Suffix (eg, Jr, III)**	Academic Degrees	Institution	Location (city, state/province, country)	Role or Contribution, eg, chair, principal investigator	Group (if more than 1 Group listed in the byline) and/or Subgroup (eg, Steering Committee)
Maureen	Whittelsey			Brigham and Women's Hospital	Boston, MA, USA	Clinical Research Coordinator	RECOVER-Adult
Rebecca	Wiener			Brigham and Women's Hospital	Boston, MA, USA	Clinical Research Coordinator	RECOVER-Adult
Ingrid V.	Bassett		MD, MPH	Massachusetts General Hospital	Boston, MA, USA	Hub PI, SubSite PI	RECOVER-Adult
George A.	Alba		MD	Massachusetts General Hospital	Boston, MA, USA	Co-Investigator	RECOVER-Adult
Taing N.	Aung			Massachusetts General Hospital	Boston, MA, USA	Project Manager	RECOVER-Adult
Leo C.	Ginns		MD	Massachusetts General Hospital	Boston, MA, USA	Co-Investigator	RECOVER-Adult
Jennifer	Haas		MD	Massachusetts General Hospital	Boston, MA, USA	Co-Investigator	RECOVER-Adult
Yanxiang	Hu		BS	Massachusetts General Hospital	Boston, MA, USA	Research Coordinator	RECOVER-Adult
Boris D.	Juelg		MD, PhD	Massachusetts General Hospital	Boston, MA, USA	Co-Investigator	RECOVER-Adult
Diane G.	Kanjilal		FNP	Massachusetts General Hospital	Boston, MA, USA	Co-Investigator	RECOVER-Adult
Arthur Y.	Kim		MD	Massachusetts General Hospital	Boston, MA, USA	Co-Investigator	RECOVER-Adult
Elizabeth B.	Klerman		MD, PhD	Massachusetts General Hospital	Boston, MA, USA	Co-Investigator	RECOVER-Adult
Gregory	Lewis		MD	Massachusetts General Hospital	Boston, MA, USA	Co-Investigator	RECOVER-Adult
Awo	Musa			Massachusetts General Hospital	Boston, MA, USA	Research Coordinator	RECOVER-Adult
Bisola	Ojikutu		MD, MPH	Massachusetts General Hospital	Boston, MA, USA	Co-Investigator	RECOVER-Adult
Roy	Perlis		MD, MSc	Massachusetts General Hospital	Boston, MA, USA	Co-Investigator	RECOVER-Adult
Jonathan	Rosand		MD, MSc	Massachusetts General Hospital	Boston, MA, USA	Co-Investigator	RECOVER-Adult
Zachary S.	Wallace		MD, MSc	Massachusetts General Hospital	Boston, MA, USA	Co-Investigator	RECOVER-Adult
Dean	Xerras		MD	Massachusetts General Hospital	Boston, MA, USA	Co-Investigator	RECOVER-Adult
Danielle	Zionts		MScPH	Massachusetts General Hospital	Boston, MA, USA	Program Manager	RECOVER-Adult
Janet M.	Mullington		PhD	Beth Israel Deaconess Medical Center and Harvard Medical School	Boston, MA, USA	SubSite PI, co-chair of the Integrative physiology TF	RECOVER-Adult
Ai-Ris	Collier			Beth Israel Deaconess Medical Center	Boston, MA, USA	Co-Investigator	RECOVER-Adult
Tamara	Fong			Beth Israel Deaconess Medical Center	Boston, MA, USA	Co-Investigator	RECOVER-Adult

Supplemental Online Content: Nonauthor Collaborators
*First name, last name, and suffix (if applicable) are required and will appear in PubMed.

***First Name and Middle Initial(s)**	***Last Name**	***Suffix (eg, Jr, III)**	Academic Degrees	Institution	Location (city, state/province, country)	Role or Contribution, eg, chair, principal investigator	Group (if more than 1 Group listed in the byline) and/or Subgroup (eg, Steering Committee)
Monika	Haack			Beth Israel Deaconess Medical Center	Boston, MA, USA	Co-Investigator	RECOVER-Adult
Kristine S.	Hauser		MS, MSN	Beth Israel Deaconess Medical Center	Boston, MA, USA	Co-Investigator, Research Nurse	RECOVER-Adult
Jason H.	Maley		MD, MS	Beth Israel Deaconess Medical Center	Boston, MA, USA	Co-Investigator	RECOVER-Adult
Yuri	Quintana		PhD	Beth Israel Deaconess Medical Center	Boston, MA, USA	Co-Investigator	RECOVER-Adult
Lynn	Shaughnessy			Beth Israel Deaconess Medical Center	Boston, MA, USA	Co-Investigator	RECOVER-Adult
Kathryn	Stephenson			Beth Israel Deaconess Medical Center	Boston, MA, USA	Co-Investigator	RECOVER-Adult
Robert J.	Thomas		MD	Beth Israel Deaconess Medical Center	Boston, MA, USA	Co-Investigator	RECOVER-Adult
Robert	Torres		MPA	Beth Israel Deaconess Medical Center	Boston, MA, USA	Long COVID/RECOVER community Representative, Community Engagement Advisor	RECOVER-Adult
Jai G.	Marathe		MD, MBBS	Boston University/Boston Medical Center	Boston, MA, USA	SubSite PI	RECOVER-Adult
Elizabeth	Duffy			Boston University/Boston Medical Center	Boston, MA, USA	Co-Investigator	RECOVER-Adult
Naomi	Hamburg		MD	Boston University/Boston Medical Center	Boston, MA, USA	Co-Investigator	RECOVER-Adult
Misaki .	Kobayashi		MPH	Boston University/Boston Medical Center	Boston, MA, USA	Program Manager	RECOVER-Adult
George T.	O'Connor		MD, MS	Boston University/Boston Medical Center	Boston, MA, USA	Co-Investigator	RECOVER-Adult

Supplemental Online Content: Nonauthor Collaborators
*First name, last name, and suffix (if applicable) are required and will appear in PubMed.

***First Name and Middle Initial(s)**	***Last Name**	***Suffix (eg, Jr, III)**	Academic Degrees	Institution	Location (city, state/province, country)	Role or Contribution, eg, chair, principal investigator	Group (if more than 1 Group listed in the byline) and/or Subgroup (eg, Steering Committee)
Fitzgerald M.	Shepherd		MD	Boston University/Boston Medical Center	Boston, MA, USA	Co-Investigator	RECOVER-Adult
Charles T.	Williams		MD	Boston University/Boston Medical Center	Boston, MA, USA	Co-Investigator	RECOVER-Adult
Haihua	Zhang		MD	Boston University/Boston Medical Center	Boston, MA, USA	Co-Investigator	RECOVER-Adult
Janice	John		PA-C, MHS, MHSDS	Cambridge Health Alliance	Somerville, MA, USA	SubSite PI	RECOVER-Adult
Amberly	Ticotsky		MPH, BSN	Cambridge Health Alliance	Somerville, MA, USA	Research Nurse	RECOVER-Adult
Honorine D.	Ward		MD	Tufts Medical Center	Boston, MA, USA	SubSite PI	RECOVER-Adult
Deborah	Blazey-Martin			Tufts Medical Center	Boston, MA, USA	Co-Investigator	RECOVER-Adult
Maher	Ghamloush		MD	Tufts Medical Center	Boston, MA, USA		RECOVER-Adult
Michael	Jordan		MD, MPH	Tufts Medical Center	Boston, MA, USA	Co-Investigator	RECOVER-Adult
Laura	Kogelman			Tufts Medical Center	Boston, MA, USA	Co-Investigator	RECOVER-Adult
Nathaniel	Erdmann		MD, PhD	University of Alabama at Birmingham	Birmingham, AL, USA	Hub PI	RECOVER-Adult
Emily B.	Levitan		ScD	University of Alabama at Birmingham	Birmingham, AL, USA	Hub PI, Co-Investigator	RECOVER-Adult
Alan T.	Tita		MD, PhD	University of Alabama at Birmingham	Birmingham, AL, USA	SubSite PI, Co-Investigator	RECOVER-Adult
Donna	Armstrong		BSN, RN	University of Alabama at Birmingham	Birmingham, AL, USA	Research Nurse	RECOVER-Adult
Susan E.	Binkley						RECOVER-Adult
Kenneth	Blackwell						RECOVER-Adult
Annalia	Causey						RECOVER-Adult
Felice	Cook						RECOVER-Adult
Julio	Domingo						RECOVER-Adult
Conner	Donahue						RECOVER-Adult
Maitlyn	Eady						RECOVER-Adult

*First Name and Middle Initial(s)	*Last Name	*Suffix (eg, Jr, III)	Academic Degrees	Institution	Location (city, state/province, country)	Role or Contribution, eg, chair, principal investigator	Group (if more than 1 Group listed in the byline) and/or Subgroup (eg, Steering Committee)
Jeffrey	Edberg						RECOVER-Adult
Kentevious	Forehand		MBA, BSN, RN	University of Alabama at Birmingham	Birmingham, AL, USA	Research Coordinator	RECOVER-Adult
Patrick	Frazier		MBA	University of Alabama at Birmingham	Birmingham, AL, USA	Director	RECOVER-Adult
Noah	Garcia-McClaney						RECOVER-Adult
Melissa	Garner						RECOVER-Adult
Brandon	Gray						RECOVER-Adult
Wanda	Hall						RECOVER-Adult
Cady	Hart						RECOVER-Adult
Camden L.	Hebson		MD	University of Alabama School of Medicine	Birmingham, AL, USA	Co-Investigator	RECOVER-Adult
Bertha	Hidalgo						RECOVER-Adult
Kaylen	Holtzapfel						RECOVER-Adult
Alexis	Jinright						RECOVER-Adult
Suzanne E.	Judd		PhD	University of Alabama at Birmingham	Birmingham, AL, USA	Chair	RECOVER-Adult
Teri	Kennedy						RECOVER-Adult
Leigh	Kirkwood						RECOVER-Adult
Megan	Maier						RECOVER-Adult
Patricia	McCormack						RECOVER-Adult
Kevin	Mitchell						RECOVER-Adult
Aoyjai	Montgomery						RECOVER-Adult
Myriam	Peralta-Carcelen		MD, MPH	University of Alabama at Birmingham	Birmingham, AL, USA	Co-Investigator	RECOVER-Adult
Juan P.	Pilco						RECOVER-Adult
Leigh	Powell						RECOVER-Adult
Jacob	Royster			University of Alabama at Birmingham	Birmingham, AL, USA	Research Coordinator	RECOVER-Adult
Rachael	Shevin						RECOVER-Adult

***First Name and Middle Initial(s)**	***Last Name**	***Suffix (eg, Jr, III)**	Academic Degrees	Institution	Location (city, state/province, country)	Role or Contribution, eg, chair, principal investigator	Group (if more than 1 Group listed in the byline) and/or Subgroup (eg, Steering Committee)
Sidney	Skipworth						RECOVER-Adult
Leah	Spurgeon						RECOVER-Adult
Courtney	Steele			University of Alabama at Birmingham	Birmingham, AL, USA	Research Assistant	RECOVER-Adult
Jane	Vines			University of Alabama at Birmingham		Research Coordinator	RECOVER-Adult
Gregory	Ware						RECOVER-Adult
Rosanne	Wilson						RECOVER-Adult
Dana	Woodruff						RECOVER-Adult
Brandon	Young		BS	University of Alabama at Birmingham	Birmingham, AL, USA	Research Coordinator	RECOVER-Adult
Mark	Gillespie					PI	RECOVER-Adult
Casey L.	Daniel		PhD, MPH	University of South Alabama Whiddon College of Medicine	Mobile, AL, USA	Co-Investigator	RECOVER-Adult
Jamie	Hansel						RECOVER-Adult
Jing	Wu						RECOVER-Adult
Thomas W.	Carton		PhD	Louisiana Public Health Institute	New Orleans, LA, USA	Hub PI	RECOVER-Adult
Lucio	Miele					PI	RECOVER-Adult
Todd	Brown						RECOVER-Adult
Erica	Sutherland						RECOVER-Adult
Jyotsna	Fuloria					PI	RECOVER-Adult
Paula	Datri						RECOVER-Adult
Michael	Hagensee						RECOVER-Adult
Cathryn	Leggio						RECOVER-Adult
Allen	Perkins						RECOVER-Adult
Amber	Trauth						RECOVER-Adult
Siobhan	Trotter						RECOVER-Adult
Alexander	Van Deerlin						RECOVER-Adult
Sharon	Weiser						RECOVER-Adult
Madeline	Young						RECOVER-Adult

Supplemental Online Content: Nonauthor Collaborators
*First name, last name, and suffix (if applicable) are required and will appear in PubMed.

***First Name and Middle Initial(s)**	***Last Name**	***Suffix (eg, Jr, III)**	Academic Degrees	Institution	Location (city, state/province, country)	Role or Contribution, eg, chair, principal investigator	Group (if more than 1 Group listed in the byline) and/or Subgroup (eg, Steering Committee)
Hassan	Ashktorab		PhD	Howard University	Washington, DC, USA	Hub PI	RECOVER-Adult
Hassan	Brim		PhD	Howard University	Washington, DC, USA	Hub PI	RECOVER-Adult
Adeyinka O.	Laiyemo		MD	Howard University	Washington, DC, USA	Hub PI	RECOVER-Adult
Zaki A.	Sherif		PhD	Howard University	Washington, DC, USA	Hub PI	RECOVER-Adult
Saima	Durrani			Howard University	Washington, DC, USA	Research Assistant	RECOVER-Adult
Ali	Nezamloo			Howard University	Washington, DC, USA	Research Assistant	RECOVER-Adult
Julius	Ngwa			Howard University	Washington, DC, USA	Biostat.	RECOVER-Adult
Noelle	Njoku			Howard University	Washington, DC, USA	Research Assistant	RECOVER-Adult
Monique P.	Gentil			Howard University	Washington, DC, USA	Research Coordinator	RECOVER-Adult
Alem	Mehari		MD	Howard University College of Medicine	Washington, DC, USA	Long COVID/RECOVER patient caregiver Representative, Co-Investigator	RECOVER-Adult
Akbar	Solemani			Howard University	Washington, DC, USA	Research Assistant	RECOVER-Adult
Linda	Chang		MD, MS	Mercy Medical Center	Baltimore, MD, USA	Hub PI	RECOVER-Adult
Paul	Thuluvath			Mercy Medical Center	Baltimore, MD, USA	Sub-Site PI	RECOVER-Adult
Mhret	Alemu			Mercy Medical Center	Baltimore, MD, USA		RECOVER-Adult
Jordan	Anderson			Mercy Medical Center	Baltimore, MD, USA	RC	RECOVER-Adult
Mahak	Chauhan			Mercy Medical Center	Baltimore, MD, USA		RECOVER-Adult
Sung	Cho			Mercy Medical Center	Baltimore, MD, USA		RECOVER-Adult
Karli	Goodman			Mercy Medical Center	Baltimore, MD, USA		RECOVER-Adult
Gandi	Lanke			Mercy Medical Center	Baltimore, MD, USA		RECOVER-Adult
Ralph	Lebron			Mercy Medical Center	Baltimore, MD, USA		RECOVER-Adult
Anurag	Maheshwari			Mercy Medical Center	Baltimore, MD, USA		RECOVER-Adult
Jina	Ok			Mercy Medical Center	Baltimore, MD, USA		RECOVER-Adult
Chau	To			Mercy Medical Center	Baltimore, MD, USA		RECOVER-Adult
Sally L.	Hodder		MD	West Virginia University	Morgantown, WV, USA	Hub PI, SubSite PI	RECOVER-Adult
James M.	Bardes		MD	West Virginia University	Morgantown, WV, USA	Co-Investigator	RECOVER-Adult

Supplemental Online Content: Nonauthor Collaborators
*First name, last name, and suffix (if applicable) are required and will appear in PubMed.

***First Name and Middle Initial(s)**	***Last Name**	***Suffix (eg, Jr, III)**	Academic Degrees	Institution	Location (city, state/province, country)	Role or Contribution, eg, chair, principal investigator	Group (if more than 1 Group listed in the byline) and/or Subgroup (eg, Steering Committee)
Daphne	Dominique-Villanueva		MD	West Virginia University	Morgantown, WV, USA	Co-Investigator	RECOVER-Adult
Joy	Juskowich		MD	West Virginia University	Morgantown, WV, USA	Co-Investigator	RECOVER-Adult
Rebecca	Reece		MD	West Virginia University	Morgantown, WV, USA	Co-Investigator	RECOVER-Adult
Arif	Sarwari		MD	West Virginia University	Morgantown, WV, USA	Co-Investigator	RECOVER-Adult
Judd	Shellito		MD	Louisiana State University	New Orleans, LA, USA	SubSite PI	RECOVER-Adult
Michael	Hagensee		MD, PhD	LSU New Orleans/University Medical Center-New Orleans	New Orleans, LA, USA		RECOVER-Adult
Lucio	Miele		MD, PhD	Louisiana State University Health Sciences Center, New Orleans	New Orleans, LA, USA	Co-Investigator	RECOVER-Adult
Frank L.	Greenway		MD	Pennington Biomedical Research Center	Baton Rouge, LA, USA	SubSite PI	RECOVER-Adult
John P.	Kirwan		PhD	Pennington Biomedical Research Center	Baton Rouge, LA, USA	PI LACATS Co-Investigator	RECOVER-Adult
Gabrielle	Rodemann			Pennington Biomedical Research Center	Baton Rouge, LA, USA	Clinic Staff	RECOVER-Adult
Clifford J.	Rosen		MD	MaineHealth	Scarborough, ME, USA	SubSite PI	RECOVER-Adult
Abigail	Arruda			MaineHealth	Scarborough, ME, USA	Clinical Research Coordinator	RECOVER-Adult
Tristan	Brunet		BS	MaineHealth	Scarborough, ME, USA	Student Researcher	RECOVER-Adult
Ivette F.	Emery		PhD	MaineHealth	Scarborough, ME, USA	Co-Investigator	RECOVER-Adult
Theresa	Roelke		NP	MaineHealth	Scarborough, ME, USA	Co-Investigator	RECOVER-Adult
Paul	Berger		MD	Sanford Health	Sioux Falls, SD, USA	Site PI	RECOVER-Adult
Susan E.	Hoover		MD, PhD	Sanford Health	Sioux Falls, SD, USA	SubSite PI	RECOVER-Adult
Lora	Black		MPH	Sanford Health	Sioux Falls, SD, USA	Co-Investigator	RECOVER-Adult
Brian	Tjarks		MD	Sanford Health	Sioux Falls, SD, USA	Co-Investigator	RECOVER-Adult
Vivian	Fonseca		MD	Tulane University Health Sciences Center	New Orleans, LA, USA	SubSite PI	RECOVER-Adult

Supplemental Online Content: Nonauthor Collaborators
*First name, last name, and suffix (if applicable) are required and will appear in PubMed.

***First Name and Middle Initial(s)**	***Last Name**	***Suffix (eg, Jr, III)**	Academic Degrees	Institution	Location (city, state/province, country)	Role or Contribution, eg, chair, principal investigator	Group (if more than 1 Group listed in the byline) and/or Subgroup (eg, Steering Committee)
Shaveeta	Gupta		MD	Tulane University Health Sciences Center	New Orleans, LA, USA	Co-Investigator	RECOVER-Adult
Michele	Longo		MD	Tulane University Health Sciences Center	New Orleans, LA, USA	Co-Investigator	RECOVER-Adult
Mei	Yang		MD	Tulane University Health Sciences Center	New Orleans, LA, USA	Co-Investigator	RECOVER-Adult
Cecilia M.	Shikuma		MD	University of Hawaii	Honolulu, HI, USA	Site PI	RECOVER-Adult
Dominic C.	Chow		MD	University of Hawaii	Honolulu, HI, USA	Co-Investigator	RECOVER-Adult
Louis	MarGangcuangco		MD	University of Hawaii	Honolulu, HI, USA	Co-Investigator	RECOVER-Adult
Mario	Castro		MD, MPH	University of Kansas Medical Center	Kansas City, KS, USA	SubSite PI	RECOVER-Adult
Charles	Bengtson		MD	University of Kansas Medical Center	Kansas City, KS, USA	Co-Investigator	RECOVER-Adult
Theresa	Howard		DNP	University of Kansas Medical Center	Kansas City, KS, USA	Co-Investigator	RECOVER-Adult
Brandon	Koontz			University of Kansas Medical Center	Kansas City, KS, USA	Co-Investigator	RECOVER-Adult
Leslie A.	Spikes		MD	University of Kansas Medical Center	Kansas City, KS, USA	Co-Investigator	RECOVER-Adult
Christopher	Simmons		MD	University of Kentucky	Lexington, KY, USA	SubSite Co-PI	RECOVER-Adult
Sidney W.	Whiteheart		PhD	University of Kentucky	Lexington, KY, USA	SubSite Co-PI	RECOVER-Adult
Beth	Garvy		PhD	University of Kentucky	Lexington, KY, USA	Co-Investigator	RECOVER-Adult
Jeremy P.	Wood		PhD	University of Kentucky	Lexington, KY, USA	Co-Investigator	RECOVER-Adult
Gailen D.	Marshall		MD, PhD, MS	University of Mississippi Medical Center	Jackson, MS, USA	SubSite PI	RECOVER-Adult
Vishnu	Garla		MD	University of Mississippi Medical Center	Jackson, MS, USA	Co-Investigator	RECOVER-Adult
Joy	Kuebler		PT	University of Mississippi Medical Center	Jackson, MS, USA	Co-Investigator	RECOVER-Adult

Supplemental Online Content: Nonauthor Collaborators
*First name, last name, and suffix (if applicable) are required and will appear in PubMed.

***First Name and Middle Initial(s)**	***Last Name**	***Suffix (eg, Jr, III)**	Academic Degrees	Institution	Location (city, state/province, country)	Role or Contribution, eg, chair, principal investigator	Group (if more than 1 Group listed in the byline) and/or Subgroup (eg, Steering Committee)
Utsav	Nandi		MD	University of Mississippi Medical Center	Jackson, MS, USA	Co-Investigator	RECOVER-Adult
Andrew	Vasey		MD	University of Nebraska Medical Center	Omaha, NE, USA	SubSite Co-PI	RECOVER-Adult
David E.	Warren		PhD	University of Nebraska Medical Center	Omaha, NE, USA	SubSite Co-PI	RECOVER-Adult
John D.	Dickinson		MD, PhD	University of Nebraska Medical Center	Omaha, NE, USA	Co-Investigator	RECOVER-Adult
Timothy M.	VanWagoner		PhD	University of Oklahoma Health Sciences Center	Oklahoma City, OK, USA	SubSite PI	RECOVER-Adult
Amanda	Bogie		MD	University of Oklahoma Health Sciences Center	Oklahoma City, OK, USA	Co-Investigator	RECOVER-Adult
Daniel J.	Heyanka		PhD	University of Oklahoma Health Sciences Center	Oklahoma City, OK, USA	Co-Investigator	RECOVER-Adult
Judith A.	James		MD	University of Oklahoma Health Sciences Center	Oklahoma City, OK, USA	Co-Investigator	RECOVER-Adult
James	Scott		PhD	University of Oklahoma Health Sciences Center	Oklahoma City, OK, USA	Co-Investigator	RECOVER-Adult
Fatima I.	Sukhera		MD	University of Oklahoma Health Sciences Center	Oklahoma City, OK, USA	Co-Investigator	RECOVER-Adult
Carlos A.	Luciano Roman		MD	University of Puerto Rico	San Juan, PR, USA	SubSite PI	RECOVER-Adult
Sigrid	Perez Frontera		MD	University of Puerto Rico	San Juan, PR, USA	Co-Investigator	RECOVER-Adult
Jorge	Santana Bagur		MD	University of Puerto Rico	San Juan, PR, USA	Co-Investigator	RECOVER-Adult
Jonathan D.	Klein		MD, MPH	Illinois Research Network (ILLInet)	Chicago, IL, USA	Hub Principal Investigator	RECOVER-Adult
Jerry A.	Krishnan		MD, PhD	Illinois Research Network (ILLInet)	Chicago, IL, USA	Hub Principal Investigator	RECOVER-Adult

*First name, last name, and suffix (if applicable) are required and will appear in PubMed.

***First Name and Middle Initial(s)**	***Last Name**	***Suffix (eg, Jr, III)**	Academic Degrees	Institution	Location (city, state/province, country)	Role or Contribution, eg, chair, principal investigator	Group (if more than 1 Group listed in the byline) and/or Subgroup (eg, Steering Committee)
Janet Y.	Lin		MD, MPH, MBA	Illinois Research Network (ILLInet)	Chicago, IL, USA	Site Principal Investigator (Mile Square Health Center)	RECOVER-Adult
Naoko	Muramatsu		PhD	Illinois Research Network (ILLInet)	Chicago, IL, USA	Hub Principal Investigator	RECOVER-Adult
Bellur S.	Prabhakar		PhD, MS	Illinois Research Network (ILLInet)	Chicago, IL, USA	Hub Principal Investigator	RECOVER-Adult
Heather M.	Prendergast		MD, MPH, MS, MHA	Illinois Research Network (ILLInet)	Chicago, IL, USA	Hub Principal Investigator	RECOVER-Adult
Terry L.	Vanden Hoek			Illinois Research Network (ILLInet)	Chicago, IL, USA	Hub Principal Investigator	RECOVER-Adult
Dara	Adams		MD	Illinois Research Network (ILLInet)	Chicago, IL, USA	Subject matter expert	RECOVER-Adult
Aileen	Baker			Illinois Research Network (ILLInet)	Chicago, IL, USA	staff	RECOVER-Adult
Sunni	Barbera			Illinois Research Network (ILLInet)	Chicago, IL, USA	staff	RECOVER-Adult
Sanjib	Basu		PhD	Illinois Research Network (ILLInet)	Chicago, IL, USA	Biostatistician	RECOVER-Adult
Susan	Bleasdale			Illinois Research Network (ILLInet)	Chicago, IL, USA	Subject matter expert	RECOVER-Adult
Andrew D.	Boyd		MD	Illinois Research Network (ILLInet)	Chicago, IL, USA	Co-investigator	RECOVER-Adult
Taylor	Breiter			Illinois Research Network (ILLInet)	Chicago, IL, USA	staff	RECOVER-Adult
Irina A.	Buhimschi		MD	Illinois Research Network (ILLInet)	Chicago, IL, USA	Co-Investigator	RECOVER-Adult
Michael D.	Carrithers		MD, PhD	Illinois Research Network (ILLInet)	Chicago, IL, USA	Co-Investigator	RECOVER-Adult

*First name, last name, and suffix (if applicable) are required and will appear in PubMed.

***First Name and Middle Initial(s)**	***Last Name**	***Suffix (eg, Jr, III)**	Academic Degrees	Institution	Location (city, state/province, country)	Role or Contribution, eg, chair, principal investigator	Group (if more than 1 Group listed in the byline) and/or Subgroup (eg, Steering Committee)
Rashmika	Chalamalla			Illinois Research Network (ILLInet)	Chicago, IL, USA	staff	RECOVER-Adult
David	Chestek		DO	Illinois Research Network (ILLInet)	Chicago, IL, USA	Co-Investigator	RECOVER-Adult
Judith A.	Cook		PhD	Illinois Research Network (ILLInet)	Chicago, IL, USA	Subject matter expert	RECOVER-Adult
Dawood	Darbar		MD	Illinois Research Network (ILLInet)	Chicago, IL, USA	Co-Investigator	RECOVER-Adult
Raktima	Dasgupta			Illinois Research Network (ILLInet)	Chicago, IL, USA	staff	RECOVER-Adult
Felicia	Davis Blakley			Illinois Research Network (ILLInet)	Chicago, IL, USA	Commmunity representative (ASI)	RECOVER-Adult
Julie A.	DeLisa			Illinois Research Network (ILLInet)	Chicago, IL, USA	staff	RECOVER-Adult
Kathleen R.	Diviak		PhD	Illinois Research Network (ILLInet)	Chicago, IL, USA	QA/QC Committee Co-Chair, Data management for local site	RECOVER-Adult
Meghan F.	Donlon			Illinois Research Network (ILLInet)	Chicago, IL, USA	staff	RECOVER-Adult
Mark S.	Dworkin		MD	Illinois Research Network (ILLInet)	Chicago, IL, USA	Subject matter expert	RECOVER-Adult
Angela	Ellison			Illinois Research Network (ILLInet)	Chicago, IL, USA	Community representative (OCEAN-HP)	RECOVER-Adult
Clarie	Flanigan			Illinois Research Network (ILLInet)	Chicago, IL, USA	staff	RECOVER-Adult
Michael B.	Freedman		MD, MPH	Illinois Research Network (ILLInet)	Chicago, IL, USA	Co-Investigator	RECOVER-Adult

Supplemental Online Content: Nonauthor Collaborators
*First name, last name, and suffix (if applicable) are required and will appear in PubMed.

***First Name and Middle Initial(s)**	***Last Name**	***Suffix (eg, Jr, III)**	Academic Degrees	Institution	Location (city, state/province, country)	Role or Contribution, eg, chair, principal investigator	Group (if more than 1 Group listed in the byline) and/or Subgroup (eg, Steering Committee)
Lynn B.	Gerald		PhD, MSPH	Illinois Research Network (ILLInet)	Chicago, IL, USA	Co-Investigator	RECOVER-Adult
Wayne H.	Giles		MD, MS	Illinois Research Network (ILLInet)	Chicago, IL, USA	Subject matter expert	RECOVER-Adult
Howard S.	Gordon		MD	Illinois Research Network (ILLInet)	Chicago, IL, USA	Subject matter expert	RECOVER-Adult
Bayan	Hammad			Illinois Research Network (ILLInet)	Chicago, IL, USA	staff	RECOVER-Adult
Sharon	Hasek			Illinois Research Network (ILLInet)	Chicago, IL, USA	staff	RECOVER-Adult
Wendy	Hasse			Illinois Research Network (ILLInet)	Chicago, IL, USA	staff	RECOVER-Adult
Martyna	Hryniewicka		MS, RN	Illinois Research Network (ILLInet)	Chicago, IL, USA	Nurse	RECOVER-Adult
Sai D.	Illendula			Illinois Research Network (ILLInet)	Chicago, IL, USA	staff	RECOVER-Adult
Nahed	Ismail		MD, PhD	Illinois Research Network (ILLInet)	Chicago, IL, USA	Co-Investigator	RECOVER-Adult
Akash	Jain			Illinois Research Network (ILLInet)	Chicago, IL, USA	staff	RECOVER-Adult
Kyle J.	Jennette		PhD	Illinois Research Network (ILLInet)	Chicago, IL, USA	Subject matter expert	RECOVER-Adult
Grace	Kadubek			Illinois Research Network (ILLInet)	Chicago, IL, USA	MPH student	RECOVER-Adult
Denise	Kent			Illinois Research Network (ILLInet)	Chicago, IL, USA	Co-Investigator	RECOVER-Adult
Denise A.	Kent		PhD	Illinois Research Network (ILLInet)	Chicago, IL, USA	Co-Investigator	RECOVER-Adult
Keri S.	Kim		PharmD, MS, CTS	Illinois Research Network (ILLInet)	Chicago, IL, USA	PIPP committee member	RECOVER-Adult

*First name, last name, and suffix (if applicable) are required and will appear in PubMed.

***First Name and Middle Initial(s)**	***Last Name**	***Suffix (eg, Jr, III)**	Academic Degrees	Institution	Location (city, state/province, country)	Role or Contribution, eg, chair, principal investigator	Group (if more than 1 Group listed in the byline) and/or Subgroup (eg, Steering Committee)
Pavitra	Kotini-Shah		MD	Illinois Research Network (ILLInet)	Chicago, IL, USA	Subject matter expert	RECOVER-Adult
Lucia	Large			Illinois Research Network (ILLInet)	Chicago, IL, USA	staff	RECOVER-Adult
James .	Lash			Illinois Research Network (ILLInet)	Chicago, IL, USA	Subject matter expert	RECOVER-Adult
Jun	Lu			Illinois Research Network (ILLInet)	Chicago, IL, USA	staff	RECOVER-Adult
Abeer M.	Mahamed		MD, PhD	Illinois Research Network (ILLInet)	Chicago, IL, USA	Subject matter expert	RECOVER-Adult
Sergey	Malchenko			Illinois Research Network (ILLInet)	Chicago, IL, USA	staff	RECOVER-Adult
Miriam	Martinez			Illinois Research Network (ILLInet)	Chicago, IL, USA	staff	RECOVER-Adult
Cammeo	Mauntel-Medici			Illinois Research Network (ILLInet)	Chicago, IL, USA	staff	RECOVER-Adult
Mark	McCauley			Illinois Research Network (ILLInet)	Chicago, IL, USA	Subject matter expert	RECOVER-Adult
Martha	Menchaca		MD, PhD	Illinois Research Network (ILLInet)	Chicago, IL, USA	Co-investigator	RECOVER-Adult
Robin	Mermelstein		PhD	Illinois Research Network (ILLInet)	Chicago, IL, USA	Co-Investigator	RECOVER-Adult
David J.	Moreno			Illinois Research Network (ILLInet)	Chicago, IL, USA	staff	RECOVER-Adult
Liam	Morrissy			Illinois Research Network (ILLInet)	Chicago, IL, USA	staff	RECOVER-Adult
Hugh	Musick			Illinois Research Network (ILLInet)	Chicago, IL, USA	Co-Investigator	RECOVER-Adult
Lourdes	Norwick			Illinois Research Network (ILLInet)	Chicago, IL, USA	staff	RECOVER-Adult

*First name, last name, and suffix (if applicable) are required and will appear in PubMed.

***First Name and Middle Initial(s)**	***Last Name**	***Suffix (eg, Jr, III)**	Academic Degrees	Institution	Location (city, state/province, country)	Role or Contribution, eg, chair, principal investigator	Group (if more than 1 Group listed in the byline) and/or Subgroup (eg, Steering Committee)
Richard M.	Novak		MD	Illinois Research Network (ILLInet)	Chicago, IL, USA	Co-Investigator	RECOVER-Adult
Marilyn	Ortiz			Illinois Research Network (ILLInet)	Chicago, IL, USA	staff	RECOVER-Adult
Khushboo	Patel			Illinois Research Network (ILLInet)	Chicago, IL, USA	staff	RECOVER-Adult
Nicolas L.	Perez			Illinois Research Network (ILLInet)	Chicago, IL, USA	staff	RECOVER-Adult
Neil H.	Pliskin		PhD	Illinois Research Network (ILLInet)	Chicago, IL, USA	Subject matter expert	RECOVER-Adult
Sam	Pope			Illinois Research Network (ILLInet)	Chicago, IL, USA	Subject matter expert	RECOVER-Adult
Bharati	Prasad		MD, MS	Illinois Research Network (ILLInet)	Chicago, IL, USA	Co-Investigator	RECOVER-Adult
Barbara	Predki			Illinois Research Network (ILLInet)	Chicago, IL, USA	staff	RECOVER-Adult
John G.	Quigley		MD	Illinois Research Network (ILLInet)	Chicago, IL, USA	Co-Investigator	RECOVER-Adult
Ramaswamy	Ramchandran		PhD	Illinois Research Network (ILLInet)	Chicago, IL, USA	Biospecimen	RECOVER-Adult
Ana	Ramirez			Illinois Research Network (ILLInet)	Chicago, IL, USA	staff	RECOVER-Adult
Sarah	Rappe			Illinois Research Network (ILLInet)	Chicago, IL, USA	staff	RECOVER-Adult
Jalees	Rehman		MD	Illinois Research Network (ILLInet)	Chicago, IL, USA	Co-Investigator	RECOVER-Adult
Matthew	Rowley			Illinois Research Network (ILLInet)	Chicago, IL, USA	staff	RECOVER-Adult
Gowrisree	Rudraraju			Illinois Research Network (ILLInet)	Chicago, IL, USA	staff	RECOVER-Adult

Supplemental Online Content: Nonauthor Collaborators
*First name, last name, and suffix (if applicable) are required and will appear in PubMed.

***First Name and Middle Initial(s)**	***Last Name**	***Suffix (eg, Jr, III)**	Academic Degrees	Institution	Location (city, state/province, country)	Role or Contribution, eg, chair, principal investigator	Group (if more than 1 Group listed in the byline) and/or Subgroup (eg, Steering Committee)
Melissa	Rutherfoord			Illinois Research Network (ILLInet)	Chicago, IL, USA	staff	RECOVER-Adult
Jennifer A.	Sculley			Illinois Research Network (ILLInet)	Chicago, IL, USA	Co-Investigator	RECOVER-Adult
Jerisha	Smith-Mack			Illinois Research Network (ILLInet)	Chicago, IL, USA	Community representative (OCEAN-HP)	RECOVER-Adult
Jun	Sun		PhD	Illinois Research Network (ILLInet)	Chicago, IL, USA	Subject matter expert	RECOVER-Adult
Nancy	Tartt			Illinois Research Network (ILLInet)	Chicago, IL, USA	Community representative (OCEAN-HP)	RECOVER-Adult
Laura	Villanueva			Illinois Research Network (ILLInet)	Chicago, IL, USA	staff	RECOVER-Adult
Sara	Warfield Kelly			Illinois Research Network (ILLInet)	Chicago, IL, USA	Co-investigator	RECOVER-Adult
Cemal	Yazici			Illinois Research Network (ILLInet)	Chicago, IL, USA	Subject matter expert	RECOVER-Adult
Marta	Certa			Illinois Research Network (ILLInet)	Chicago, IL, USA	Commmunity representative (ASI)	RECOVER-Adult
Erica	Chessier			Illinois Research Network (ILLInet)	Peoria, IL, USA	staff	RECOVER-Adult
Emily	Everett			Illinois Research Network (ILLInet)	Peoria, IL, USA	staff	RECOVER-Adult
Elijah	Kindred			Illinois Research Network (ILLInet)	Chicago, IL, USA	Site Principal Investigator (BrightStar)	RECOVER-Adult
Pastor C.	Harris			Illinois Research Network (ILLInet)	Chicago, IL, USA	Community representative (BrightStar)	RECOVER-Adult

Supplemental Online Content: Nonauthor Collaborators
*First name, last name, and suffix (if applicable) are required and will appear in PubMed.

***First Name and Middle Initial(s)**	***Last Name**	***Suffix (eg, Jr, III)**	Academic Degrees	Institution	Location (city, state/province, country)	Role or Contribution, eg, chair, principal investigator	Group (if more than 1 Group listed in the byline) and/or Subgroup (eg, Steering Committee)
Praveen	Sudhindra		MD, FACP	Illinois Research Network (ILLInet)	Peoria, IL, USA	staff	RECOVER-Adult
Lela	Olds			Illinois Research Network (ILLInet)	Chicago, IL, USA	Director of external affairs	RECOVER-Adult
Lisa	Aponte-Soto		PhD	Illinois Research Network (ILLInet)	Chicago, IL, USA	Site Principal Investigator (Illinois Unidos)	RECOVER-Adult
Marina	Del Rios			Illinois Research Network (ILLInet)	Iowa City, IA, USA	Community representative (Illinois Unidos)	RECOVER-Adult
Maya Z.	Diaz			Illinois Research Network (ILLInet)	Chicago, IL, USA	Community representative (Illinois Unidos)	RECOVER-Adult
Alejandra L.	Ibanez			Illinois Research Network (ILLInet)	Chicago, IL, USA	Community representative (Unidos)	RECOVER-Adult
Cesar	Rolon			Illinois Research Network (ILLInet)	Chicago, IL, USA	Community representative	RECOVER-Adult
Savannah	Cranford			Illinois Research Network (ILLInet)	Peoria, IL, USA	Community representative (Unity Point)	RECOVER-Adult
Daniel	Brown			Illinois Research Network (ILLInet)	Peoria, IL, USA	staff - OSF	RECOVER-Adult
Jennifer	Dixon			Illinois Research Network (ILLInet)	Peoria, IL, USA	staff	RECOVER-Adult
Lisa	Gale			Illinois Research Network (ILLInet)	Peoria, IL, USA	staff	RECOVER-Adult
Savannah	Hammerl			Illinois Research Network (ILLInet)	Peoria, IL, USA	staff	RECOVER-Adult
Kimberly	Hartwig			Illinois Research Network (ILLInet)	Peoria, IL, USA	staff	RECOVER-Adult

Supplemental Online Content: Nonauthor Collaborators
*First name, last name, and suffix (if applicable) are required and will appear in PubMed.

***First Name and Middle Initial(s)**	***Last Name**	***Suffix (eg, Jr, III)**	Academic Degrees	Institution	Location (city, state/province, country)	Role or Contribution, eg, chair, principal investigator	Group (if more than 1 Group listed in the byline) and/or Subgroup (eg, Steering Committee)
Abhigna	Madineni			Illinois Research Network (ILLInet)	Peoria, IL, USA	Peoria	RECOVER-Adult
Peyton	Swearingen			Illinois Research Network (ILLInet)	Peoria, IL, USA	staff	RECOVER-Adult
Monica	Hendrickson			Illinois Research Network (ILLInet)	Peoria, IL, USA	Site Principal Investigator (Peoria City / County Health Department)	RECOVER-Adult
Seth	Noland			Illinois Research Network (ILLInet)	Peoria, IL, USA	Recruiter	RECOVER-Adult
Tracy	Terlinde			Illinois Research Network (ILLInet)	Peoria, IL, USA	Epidemiologist	RECOVER-Adult
Brianna	Hobbs			Illinois Research Network (ILLInet)	Chicago, IL, USA	Health and research coordinator	RECOVER-Adult
Sarah A.	Stewart de Ramirez		MD, MPH, MS	Illinois Research Network (ILLInet)	Peoria, IL, USA	Hub Principal Investigator	RECOVER-Adult
Dawn	Bolliger			Illinois Research Network (ILLInet)	Peoria, IL, USA	staff - OSF	RECOVER-Adult
Jerusha	Boyineni		PhD	Illinois Research Network (ILLInet)	Peoria, IL, USA	Co-Investigator	RECOVER-Adult
Praneeth	Chebrolu			Illinois Research Network (ILLInet)	Peoria, IL, USA	staff	RECOVER-Adult
Hannah L.	Curry			Illinois Research Network (ILLInet)	Peoria, IL, USA	staff	RECOVER-Adult
Sarah E.	Donohue		PhD	Illinois Research Network (ILLInet)	Peoria, IL, USA	Co-Investigator	RECOVER-Adult
Sherrie	Edmonds			Illinois Research Network (ILLInet)	Peoria, IL, USA	staff	RECOVER-Adult

*First name, last name, and suffix (if applicable) are required and will appear in PubMed.

***First Name and Middle Initial(s)**	***Last Name**	***Suffix (eg, Jr, III)**	Academic Degrees	Institution	Location (city, state/province, country)	Role or Contribution, eg, chair, principal investigator	Group (if more than 1 Group listed in the byline) and/or Subgroup (eg, Steering Committee)
Sara W.	Kelly		PhD, MPH	Illinois Research Network (ILLInet)	Peoria, IL, USA	Co-Investigator	RECOVER-Adult
Phoebe	Maholovich			Illinois Research Network (ILLInet)	Peoria, IL, USA	staff	RECOVER-Adult
Samer B.	Sader		MD	Illinois Research Network (ILLInet)	Peoria, IL, USA	Co-Investigator	RECOVER-Adult
Tiffany	Thompson			Illinois Research Network (ILLInet)	Peoria, IL, USA	Subject matter expert	RECOVER-Adult
Hannah	Welter			Illinois Research Network (ILLInet)	Chicago, IL, USA	staff	RECOVER-Adult
Brittany	Woolley			Illinois Research Network (ILLInet)	Peoria, IL, USA	staff	RECOVER-Adult
John	Hafner		MD	Illinois Research Network (ILLInet)	Peoria, IL, USA	Co-investigator	RECOVER-Adult
Keith A.	Hanson		MD, PhD	Illinois Research Network (ILLInet)	Peoria, IL, USA	Co-Investigator	RECOVER-Adult
Robert	Hutton			Illinois Research Network (ILLInet)	Peoria, IL, USA	staff	RECOVER-Adult
Alexander W.	Charney		MD, PhD	Icahn School of Medicine at Mount Sinai	New York, NY, USA	Hub PI	RECOVER-Adult
Patricia	Kovatch		PhD	Icahn School of Medicine at Mount Sinai	New York, NY, USA	MPI, Scientific Computing	RECOVER-Adult
Miriam	Merad		MD, PhD	Icahn School of Medicine at Mount Sinai	New York, NY, USA	MPI, Immunology Lead	RECOVER-Adult
Girish N.	Nadkarni		MD, MPH	Icahn School of Medicine at Mount SInai	New York, NY, USA	Hub MPI	RECOVER-Adult
Juan P.	Wisnivesky		MD, DrPH	Icahn School of Medicine at Mount Sinai	New York, NY, USA	Hub PI	RECOVER-Adult
Judith A.	Aberg		MD	Icahn School of Medicine at Mount Sinai	New York, NY, USA	Committee member	RECOVER-Adult

*First name, last name, and suffix (if applicable) are required and will appear in PubMed.

***First Name and Middle Initial(s)**	***Last Name**	***Suffix (eg, Jr, III)**	Academic Degrees	Institution	Location (city, state/province, country)	Role or Contribution, eg, chair, principal investigator	Group (if more than 1 Group listed in the byline) and/or Subgroup (eg, Steering Committee)
Steven	Ascolillo		Bachelor	Icahn School of Medicine at Mount Sinai	New York, NY, USA	Project Manager	RECOVER-Adult
Emilia	Bagiella		PhD	Icahn School of Medicine at Mount Sinai	New York, NY, USA	Committee member	RECOVER-Adult
Logan	Bartram		MD	Icahn School of Medicine at Mount Sinai	New York, NY, USA	Professor	RECOVER-Adult
Jacqueline	Becker		PhD	Icahn School of Medicine at Mount Sinai	New York, NY, USA	Clinical Psychologist	RECOVER-Adult
Noam D.	Beckmann		PhD	Icahn School of Medicine at Mount Sinai	New York, NY, USA	Omics Committee member	RECOVER-Adult
Ashley	Bendl		Bachelor	Icahn School of Medicine at Mount Sinai	New York, NY, USA	Clinical Research Coordinator	RECOVER-Adult
Benjamin K.	Chen		MD, PhD	Icahn School of Medicine at Mount Sinai	New York, NY, USA	Co-chair mechanistic pathways task force committee	RECOVER-Adult
Alyssa	Civil		Bachelor	Icahn School of Medicine at Mount Sinai	New York, NY, USA	Project Manager	RECOVER-Adult
Ginger Y.	Crawford		BME/IT	Icahn School of Medicine at Mount Sinai	New York, NY, USA	Clinical Research Coordinator	RECOVER-Adult
Kaberi	Dhar		MS	Icahn School of Medicine at Mount Sinai	New York, NY, USA	Clinical Research Coordinator	RECOVER-Adult
Lorraine	Evo-Ortega		MPH	Icahn School of Medicine at Mount Sinai	New York, NY, USA	Clinical Research Coordinator	RECOVER-Adult
Daniel	Fierer		MD	Icahn School of Medicine at Mount Sinai	New York, NY, USA	Professor	RECOVER-Adult
Emily J.	Gallagher		MD, PhD	Icahn School of Medicine at Mount Sinai	New York, NY, USA	Chair metabolic disorders committee	RECOVER-Adult
Adolfo	Garcia-Sastre		PhD	Icahn School of Medicine at Mount Sinai	New York, NY, USA	Committee member	RECOVER-Adult

*First name, last name, and suffix (if applicable) are required and will appear in PubMed.

***First Name and Middle Initial(s)**	***Last Name**	***Suffix (eg, Jr, III)**	Academic Degrees	Institution	Location (city, state/province, country)	Role or Contribution, eg, chair, principal investigator	Group (if more than 1 Group listed in the byline) and/or Subgroup (eg, Steering Committee)
Sacha	Gnjatic		PhD	Icahn School of Medicine at Mount Sinai	New York, NY, USA	Committee member	RECOVER-Adult
Ian	Gray			Icahn School of Medicine at Mount Sinai	New York, NY, USA	Clinical Research Coordinator	RECOVER-Adult
Sabina	Guliyeva		MS	Icahn School of Medicine at Mount Sinai	New York, NY, USA	Program Manager	RECOVER-Adult
Lori	Harvey-Ingram		Bachelor	Icahn School of Medicine at Mount Sinai	New York, NY, USA	Clinical Research Coordinator	RECOVER-Adult
Julia	Herrera-Moreno		MD, MBA, MS	Icahn School of Medicine at Mount Sinai	New York, NY, USA	Clinical Research Coordinator	RECOVER-Adult
Matthew	Hill		Bachelor	Icahn School of Medicine at Mount Sinai	New York, NY, USA	Clinical Research Coordinator	RECOVER-Adult
Carol R.	Horowitz		MD, MPH	Icahn School of Medicine at Mount Sinai	New York, NY, USA	Co-Investigator, Steering committee chair	RECOVER-Adult
Rachel	Jackson		Bachelor	Icahn School of Medicine at Mount Sinai	New York, NY, USA	Associate Researcher I	RECOVER-Adult
Din	Kastrat		Bachelor	Icahn School of Medicine at Mount Sinai	New York, NY, USA	Clinical Research Coordinator	RECOVER-Adult
Anu	Lala-Trindade		MD	Icahn School of Medicine at Mount Sinai	New York, NY, USA	Committee member	RECOVER-Adult
Jenny	Lin		MD, MPH	Icahn School of Medicine at Mount Sinai	New York, NY, USA	Clinical cohort logistics and management	RECOVER-Adult
Nick	Macaluso		Bachelor	Icahn School of Medicine at Mount Sinai	New York, NY, USA	Clinical Research Coordinator	RECOVER-Adult
Kathryn	Marcon		MPH	Icahn School of Medicine at Mount Sinai	New York, NY, USA	Project Manager	RECOVER-Adult

Supplemental Online Content: Nonauthor Collaborators
*First name, last name, and suffix (if applicable) are required and will appear in PubMed.

***First Name and Middle Initial(s)**	***Last Name**	***Suffix (eg, Jr, III)**	Academic Degrees	Institution	Location (city, state/province, country)	Role or Contribution, eg, chair, principal investigator	Group (if more than 1 Group listed in the byline) and/or Subgroup (eg, Steering Committee)
Dara	Meyer		MS	Icahn School of Medicine at Mount Sinai	New York, NY, USA	Director of Operations and Project Management	RECOVER-Adult
Janice	Morinigo		Bachelor	Icahn School of Medicine at Mount Sinai	New York, NY, USA	Research Laboratory Manager	RECOVER-Adult
Benjamin H.	Natelson		MD	Icahn School of Medicine at Mount Sinai	New York, NY, USA	Committee member	RECOVER-Adult
Maya	Nussenzweig		MPH	Icahn School of Medicine at Mount Sinai	New York, NY, USA	Compliance Coordinator	RECOVER-Adult
Tiffani	Padua		Bachelor	Icahn School of Medicine at Mount Sinai	New York, NY, USA	Clinical Research Coordinator	RECOVER-Adult
David	Putrino		PT, PhD	Icahn School of Medicine at Mount Sinai	New York, NY, USA	Committee member	RECOVER-Adult
Lynne	Richardson		MD	Icahn School of Medicine at Mount Sinai	New York, NY, USA	Committee member	RECOVER-Adult
Scott	Russo		PhD	Icahn School of Medicine at Mount Sinai	New York, NY, USA	Committee member	RECOVER-Adult
Alan C.	Seifert		PhD	Icahn School of Medicine at Mount Sinai	New York, NY, USA	Co-Investigator, Neuroradiology Co-Lead	RECOVER-Adult
Abdullah	Serri		Bachelor	Icahn School of Medicine at Mount Sinai	New York, NY, USA	Clinical Research Coordinator	RECOVER-Adult
Jordan	Walker		Bachelor	Icahn School of Medicine at Mount Sinai	New York, NY, USA	Clinical Research Coordinator	RECOVER-Adult
Michell	Yee		Bachelor	Icahn School of Medicine at Mount Sinai	New York, NY, USA	Associate Researcher	RECOVER-Adult
Lucinda	Bateman		MD	University of Utah	Salt Lake City, UT, USA	SubSite PI	RECOVER-Adult
Rachel	Hess		MD, MS	University of Utah	Salt Lake City, UT, USA	Hub PI	RECOVER-Adult
Dongngan T.	Truong		MD, MS	University of Utah	Salt Lake City, UT, USA	Hub PI	RECOVER-Adult

Supplemental Online Content: Nonauthor Collaborators
*First name, last name, and suffix (if applicable) are required and will appear in PubMed.

***First Name and Middle Initial(s)**	***Last Name**	***Suffix (eg, Jr, III)**	Academic Degrees	Institution	Location (city, state/province, country)	Role or Contribution, eg, chair, principal investigator	Group (if more than 1 Group listed in the byline) and/or Subgroup (eg, Steering Committee)
Natalya	Alekhina		MS	University of Utah	Salt Lake City, UT, USA	Not provided	RECOVER-Adult
Jackson	Barlocker		MS	University of Utah	Salt Lake City, UT, USA	Data Engineer	RECOVER-Adult
Jeanette P.	Brown		MD, PhD	University of Utah			RECOVER-Adult
Melissa	Cortez		DO	University of Utah			RECOVER-Adult
Dagny K	Donohue		BS	University of Utah			RECOVER-Adult
Julio C.	Facelli		PhD	University of Utah	SALT LAKE CITY, UT, USA	Co-Investigator	RECOVER-Adult
Isaac	Ford		DpT	University of Utah			RECOVER-Adult
Ramkiran	Gouripeddi		PhD	University of Utah	Salt Lake City, UT, USA	Co-Investigator	RECOVER-Adult
Jessica A.	Hermansen		BS	University of Utah			RECOVER-Adult
Jace D.	Johnny		DNP	University of Utah	Salt Lake City, UT, USA	Clinician/Annotation	RECOVER-Adult
Ashton M.	Lindsay		SCMT, MTBC	University of Utah			RECOVER-Adult
Leah	Ling			University of Utah			RECOVER-Adult
Jennifer	Lloyd		MSN	University of Utah	Salt Lake City, UT, USA	Research Nurse	RECOVER-Adult
Yue	Lu		PhD	University of Utah			RECOVER-Adult
Juliemar C.	Medina			University of Utah			RECOVER-Adult
Sarah Shizuko	Morimoto		PsyD	University of Utah			RECOVER-Adult
Laura A.	Pace		MD, PhD	University of Utah			RECOVER-Adult
Jenny M.	Powell		BS	University of Utah			RECOVER-Adult
Mary Beth	Scholand		MD	University of Utah			RECOVER-Adult
Kevin S.	Shah		MD	University of Utah			RECOVER-Adult
Nasser	Sharareh		PhD	University of Utah			RECOVER-Adult
Adam M.	Spivak		MD, MS	University of Utah			RECOVER-Adult
Caitlyn	Stringham			University of Utah			RECOVER-Adult
Joel D.	Trinity		PhD	University of Utah	Salt Lake City, UT, USA	Scientific Lead, Task Force Member - Integrative Physiology	RECOVER-Adult
Matt	Velinder		PhD	University of Utah			RECOVER-Adult

Supplemental Online Content: Nonauthor Collaborators
*First name, last name, and suffix (if applicable) are required and will appear in PubMed.

*First Name and Middle Initial(s)	*Last Name	*Suffix (eg, Jr, III)	Academic Degrees	Institution	Location (city, state/province, country)	Role or Contribution, eg, chair, principal investigator	Group (if more than 1 Group listed in the byline) and/or Subgroup (eg, Steering Committee)
Lisa J.	Weaver		BS	University of Utah			RECOVER-Adult
Lucinda	Bateman		MD	Bateman Horne Center		SubSite PI	RECOVER-Adult
Suzanne D.	Vernon		PhD	Bateman Horne Center			RECOVER-Adult
Sara J.	Deakyne Davies		MPH	Children's Hospital Colorado	Aurora, CO, USA	Subsite Informatics Lead, Co-Investigator	RECOVER-Adult
Edward M.	Gardner		MD	Denver Health & Hospital Authority	Denver, CO, USA	SubSite PI	RECOVER-Adult
Tanner W.	Bryan		MS, CCRP	Denver Health & Hospital Authority	Denver, CO, USA	Research Manager	RECOVER-Adult
Kaitlin E.	Buck		MPH	Denver Health & Hospital Authority	Denver, CO, USA	Clinical Research Coordinator	RECOVER-Adult
Kellie L.	Hawkins		MD, MPH	Denver Health & Hospital Authority	Denver, CO, USA	Co-Investigator	RECOVER-Adult
Judy L.	Oakes		PhD	Denver Health & Hospital Authority	Denver, CO, USA	Clinical Research Coordinator	RECOVER-Adult
Benjamin D.	Horne		PhD, MPH, MStat	Intermountain Healthcare	Salt Lake City, UT, USA	SubSite PI	RECOVER-Adult
Kirk	Knowlton		MD	Intermountain Healthcare	Salt Lake City, UT, USA	Site PI	RECOVER-Adult
Scott C.	Woller		MD	Intermountain Healthcare	Murray, UT, USA	SubSite PI, Co-Investigator	RECOVER-Adult
Bailee	Aguirre			Intermountain Healthcare			RECOVER-Adult
Jeff	Anderson		MD	Intermountain Healthcare	Salt Lake City, UT, USA	Co-Investigator	RECOVER-Adult
Tami	Bair			Intermountain Healthcare			RECOVER-Adult
Lindsay	Bosh			Intermountain Healthcare			RECOVER-Adult
Lorlie	Evans			Intermountain Healthcare			RECOVER-Adult
Chase	Garrett		BS	Intermountain Healthcare			RECOVER-Adult
Dixie	Harris			Intermountain Healthcare			RECOVER-Adult
Katherine	Herrera			Intermountain Healthcare			RECOVER-Adult
Leslie	Iverson		PA-C	Intermountain Healthcare	Salt Lake City, UT, USA	Co-Investigator	RECOVER-Adult

Supplemental Online Content: Nonauthor Collaborators
*First name, last name, and suffix (if applicable) are required and will appear in PubMed.

*First Name and Middle Initial(s)	*Last Name	*Suffix (eg, Jr, III)	Academic Degrees	Institution	Location (city, state/province, country)	Role or Contribution, eg, chair, principal investigator	Group (if more than 1 Group listed in the byline) and/or Subgroup (eg, Steering Committee)
McKenna M.	Jensen		BS	Intermountain Healthcare	Salt Lake City, UT, USA	Clinical Research Coordinator	RECOVER-Adult
James	Juan			Intermountain Healthcare			RECOVER-Adult
Stacey	Knight		PhD	Intermountain Healthcare	Salt Lake City, UT, USA	Biostatistician	RECOVER-Adult
Lindsay	Leither		MD	Intermountain Healthcare	Salt Lake City, UT, USA	Co-investigator	RECOVER-Adult
Heather	Maestas		BS	Intermountain Healthcare			RECOVER-Adult
Heidi T.	May		PhD, MPH	Intermountain Healthcare	Salt Lake City, UT, USA	Co-Investigator	RECOVER-Adult
Gabriel	Najarian			Intermountain Healthcare			RECOVER-Adult
Tiaura	Webb			Intermountain Healthcare	Salt Lake City, UT, USA	Clinical Research Assistant	RECOVER-Adult
Shyanne	Zubal			Intermountain Healthcare			RECOVER-Adult
Kristine M.	Erlandson		MD, MS	University of Colorado, Anschutz Medical Campus	Aurora, CO, USA	SubSite PI	RECOVER-Adult
Ron J.	Sokol		MD	University of Colorado, Anschutz Medical Campus	Aurora, CO, USA	Site PI	RECOVER-Adult
Marisa	Brightman		PA	University of Colorado, Anschutz Medical Campus	Aurora, CO, USA	Co-Investigator	RECOVER-Adult
Debra	Davis		NP, PMHNP	University of Colorado, Anschutz Medical Campus	Aurora, CO, USA	Psychiatrist	RECOVER-Adult
Elen M.	Feuerriegel		PhD, ACRP-CP	University of Colorado, Anschutz Medical Campus	Aurora, CO, USA	Research Manager	RECOVER-Adult
Harrison Z.	Fudge		BS	University of Colorado, Anschutz Medical Campus	Aurora, CO, USA	Clinical Research Coordinator	RECOVER-Adult
Lohit	Garg		MD	University of Colorado, Anschutz Medical Campus	Aurora, CO, USA	Co-Investigator	RECOVER-Adult
Janine	Higgins		PhD	University of Colorado, Anschutz Medical Campus	Aurora, CO, USA	Co-Investigator	RECOVER-Adult
Sarah E.	Jolley		MD, MS	University of Colorado, Anschutz Medical Campus	Aurora, CO, USA	Co-Investigator, Adjudication Committee Chair	RECOVER-Adult

*First name, last name, and suffix (if applicable) are required and will appear in PubMed.

*First Name and Middle Initial(s)	*Last Name	*Suffix (eg, Jr, III)	Academic Degrees	Institution	Location (city, state/province, country)	Role or Contribution, eg, chair, principal investigator	Group (if more than 1 Group listed in the byline) and/or Subgroup (eg, Steering Committee)
Tim	Lockie		MS, MBA, CRA	University of Colorado, Anschutz Medical Campus	Aurora, CO, USA	Research Staff	RECOVER-Adult
Sean A.	McCandless		BS	University of Colorado, Anschutz Medical Campus	Aurora, CO, USA	Clinical Research Coordinator	RECOVER-Adult
Chloe	Pitch		BS, MA	University of Colorado, Anschutz Medical Campus	Aurora, CO, USA	Clinical Research Coordinator	RECOVER-Adult
Jane E.	Reusch		MD	University of Colorado, Anschutz Medical Campus	Aurora, CO, USA	Co-Investigator, committee chair	RECOVER-Adult
Brook	Thurman		BS	University of Colorado, Anschutz Medical Campus	Aurora, CO, USA	Research Staff	RECOVER-Adult
Huong	Tran		BA	University of Colorado, Anschutz Medical Campus	Aurora, CO, USA	Clinical Research Coordinator	RECOVER-Adult
Shelby C.	West		MPH	University of Colorado, Anschutz Medical Campus	Aurora, CO, USA	Clinical Research Coordinator	RECOVER-Adult
Naomi P.	Friedman		PhD	University of Colorado, Boulder	Boulder, CO, USA	SubSite PI	RECOVER-Adult
Katelyn R.	Ludwig		PhD	University of Colorado, Boulder	Boulder, CO, USA	Research Scientist	RECOVER-Adult
Lauren A.	Decker		MD	University of New Mexico	Albuquerque, NM, USA	Hub PI	RECOVER-Adult
Hengameh	Raissy		PharmD	University of New Mexico	Albuquerque, NM, USA	Hub PI	RECOVER-Adult
Natalie L.	Adolphi		PhD	University of New Mexico	Albuquerque, NM, USA	Co-Investigator	RECOVER-Adult
David A.	Archuleta			University of New Mexico			RECOVER-Adult
Steven B.	Bradfute		PhD	University of New Mexico	Albuquerque, NM, USA	Co-Investigator	RECOVER-Adult
Rebecca	Brito			University of New Mexico			RECOVER-Adult
Jamie	Elifritz		MD	University of New Mexico	Albuquerque, NM, USA	Co-Investigator	RECOVER-Adult
Noella D.	Garcia-Soberanez		BA	University of New Mexico			RECOVER-Adult

***First Name and Middle Initial(s)**	***Last Name**	***Suffix (eg, Jr, III)**	Academic Degrees	Institution	Location (city, state/province, country)	Role or Contribution, eg, chair, principal investigator	Group (if more than 1 Group listed in the byline) and/or Subgroup (eg, Steering Committee)
Frederick D.	Gentry		RN	University of New Mexico			RECOVER-Adult
Michelle S.	Harkins		MD	University of New Mexico	Albuquerque, NM, USA	Co-Investigator	RECOVER-Adult
Noah I.	Martinez		BS	University of New Mexico			RECOVER-Adult
Lorenzo A.	Montoya		BS	University of New Mexico			RECOVER-Adult
Alisha N.	Parada		MD	University of New Mexico	Albuquerque, NM, USA	Co-Investigator	RECOVER-Adult
Davin K.	Quinn		MD	University of New Mexico	Albuquerque, NM, USA	Co-Investigator	RECOVER-Adult
Alfredo	Ramos		RN	University of New Mexico			RECOVER-Adult
Elyce B.	Sheehan		MD	University of New Mexico	Albuquerque, NM, USA	Co-Investigator	RECOVER-Adult
Irena S.	Treacher		MA	University of New Mexico			RECOVER-Adult
Grace	McComsey		MD	Case Western Reserve University and University Hospitals of Cleveland	Cleveland, OH, USA	Hub PI	RECOVER-Adult
Cara	Adams			Case Western Reserve University and University Hospitals of Cleveland			RECOVER-Adult
John	Andrefsky		MD	Case Western Reserve University and University Hospitals of Cleveland			RECOVER-Adult
Ornina	Atieh		MD	Case Western Reserve University and University Hospitals of Cleveland			RECOVER-Adult
Jhonny	Baissary		MD	Case Western Reserve University and University Hospitals of Cleveland			RECOVER-Adult
Nicholas	Boldt			Case Western Reserve University and University Hospitals of Cleveland			RECOVER-Adult

Supplemental Online Content: Nonauthor Collaborators
*First name, last name, and suffix (if applicable) are required and will appear in PubMed.

***First Name and Middle Initial(s)**	***Last Name**	***Suffix (eg, Jr, III)**	Academic Degrees	Institution	Location (city, state/province, country)	Role or Contribution, eg, chair, principal investigator	Group (if more than 1 Group listed in the byline) and/or Subgroup (eg, Steering Committee)
Ann	Conrad		CNP	Case Western Reserve University and University Hospitals of Cleveland			RECOVER-Adult
Brian	D'Anza		MD	Case Western Reserve University and University Hospitals of Cleveland			RECOVER-Adult
Ziad	Koberssy		MD	Case Western Reserve University and University Hospitals of Cleveland			RECOVER-Adult
Joviane	Daher		MD	Case Western Reserve University and University Hospitals of Cleveland			RECOVER-Adult
Sarah	Dawson			Case Western Reserve University and University Hospitals of Cleveland			RECOVER-Adult
Kathryn	DiFrancesco			Case Western Reserve University and University Hospitals of Cleveland			RECOVER-Adult
Jared	Durieux			Case Western Reserve University and University Hospitals of Cleveland			RECOVER-Adult
Theresa	Foster (Rodgers)			Case Western Reserve University and University Hospitals of Cleveland			RECOVER-Adult
Michelle	Gallagher			Case Western Reserve University and University Hospitals of Cleveland			RECOVER-Adult
Amit	Gupta		MD	Case Western Reserve University and University Hospitals of Cleveland			RECOVER-Adult

Supplemental Online Content: Nonauthor Collaborators
*First name, last name, and suffix (if applicable) are required and will appear in PubMed.

***First Name and Middle Initial(s)**	***Last Name**	***Suffix (eg, Jr, III)**	Academic Degrees	Institution	Location (city, state/province, country)	Role or Contribution, eg, chair, principal investigator	Group (if more than 1 Group listed in the byline) and/or Subgroup (eg, Steering Committee)
Jami	Harrill			Case Western Reserve University and University Hospitals of Cleveland			RECOVER-Adult
Paul	Harris			Case Western Reserve University and University Hospitals of Cleveland			RECOVER-Adult
Carla	Hernandez			Case Western Reserve University and University Hospitals of Cleveland			RECOVER-Adult
Sarah	Ialacci			Case Western Reserve University and University Hospitals of Cleveland			RECOVER-Adult
Frank	Jacono		MD	Case Western Reserve University and University Hospitals of Cleveland			RECOVER-Adult
Jordyn	Kelly			Case Western Reserve University and University Hospitals of Cleveland			RECOVER-Adult
Rohini	Kumar			Case Western Reserve University and University Hospitals of Cleveland			RECOVER-Adult
Danielle	Labbato			Case Western Reserve University and University Hospitals of Cleveland			RECOVER-Adult
Elizabeth	Lesco		MD	Case Western Reserve University and University Hospitals of Cleveland			RECOVER-Adult
Joaquin	Lim			Case Western Reserve University and University Hospitals of Cleveland			RECOVER-Adult

*First name, last name, and suffix (if applicable) are required and will appear in PubMed.

***First Name and Middle Initial(s)**	***Last Name**	***Suffix (eg, Jr, III)**	Academic Degrees	Institution	Location (city, state/province, country)	Role or Contribution, eg, chair, principal investigator	Group (if more than 1 Group listed in the byline) and/or Subgroup (eg, Steering Committee)
Kimberly	Pettinato		MD, MPH	Case Western Reserve University and University Hospitals of Cleveland			RECOVER-Adult
Michael	Rodgers			Case Western Reserve University and University Hospitals of Cleveland			RECOVER-Adult
Breandan	Rosolia			Case Western Reserve University and University Hospitals of Cleveland			RECOVER-Adult
Arnab	Roy			Case Western Reserve University and University Hospitals of Cleveland			RECOVER-Adult
Sarah	Scott		MD	Case Western Reserve University and University Hospitals of Cleveland			RECOVER-Adult
Beth	Smith			Case Western Reserve University and University Hospitals of Cleveland			RECOVER-Adult
Viral	Tejani		phd	Case Western Reserve University and University Hospitals of Cleveland			RECOVER-Adult
Megan	Tribout			Case Western Reserve University and University Hospitals of Cleveland			RECOVER-Adult
George	Yendewa		MD	Case Western Reserve University and University Hospitals of Cleveland			RECOVER-Adult
David	Zhang		MD	Case Western Reserve University and University Hospitals of Cleveland			RECOVER-Adult

Supplemental Online Content: Nonauthor Collaborators
*First name, last name, and suffix (if applicable) are required and will appear in PubMed.

***First Name and Middle Initial(s)**	***Last Name**	***Suffix (eg, Jr, III)**	Academic Degrees	Institution	Location (city, state/province, country)	Role or Contribution, eg, chair, principal investigator	Group (if more than 1 Group listed in the byline) and/or Subgroup (eg, Steering Committee)
Nora G.	Singer		MD	The MetroHealth System, Case Western Reserve University	Cleveland, OH, USA	SubSite PI, MULTI-PI	RECOVER-Adult
Mirna	Ayache		MD, MPH	The MetroHealth System, Case Western Reserve University	Cleveland, OH, USA	Co-Investigator	RECOVER-Adult
Emma	Barnboym			The MetroHealth System, Case Western Reserve University			RECOVER-Adult
Alexis	Brown			The MetroHealth System, Case Western Reserve University			RECOVER-Adult
Hailey	Chesnick			The MetroHealth System, Case Western Reserve University			RECOVER-Adult
Marissa	Edminston			The MetroHealth System, Case Western Reserve University			RECOVER-Adult
Carla	Greenwood			The MetroHealth System, Case Western Reserve University			RECOVER-Adult
Maricela	Haghiac			The MetroHealth System, Case Western Reserve University			RECOVER-Adult
Elizabeth	Kaufman		MD	The MetroHealth System, Case Western Reserve University	Cleveland, OH, USA	Co-Investigator, Reading ECGs, Seeing POTS patients	RECOVER-Adult
Ketrin	Lengu			The MetroHealth System, Case Western Reserve University			RECOVER-Adult
Rebecca	Lowenthal			The MetroHealth System, Case Western Reserve University			RECOVER-Adult
Shahdi	Malakooti		MD, MS	The MetroHealth System, Case Western Reserve University	Cleveland, OH, USA	Co-Investigator	RECOVER-Adult
Christine	Oleson			The MetroHealth System, Case Western Reserve University			RECOVER-Adult
Ann	Pearman		PhD	The MetroHealth System, Case Western Reserve University	Cleveland, OH, USA	Co-Investigator	RECOVER-Adult

*First name, last name, and suffix (if applicable) are required and will appear in PubMed.

***First Name and Middle Initial(s)**	***Last Name**	***Suffix (eg, Jr, III)**	Academic Degrees	Institution	Location (city, state/province, country)	Role or Contribution, eg, chair, principal investigator	Group (if more than 1 Group listed in the byline) and/or Subgroup (eg, Steering Committee)
Allison	Rizea			The MetroHealth System, Case Western Reserve University			RECOVER-Adult
Cheryl	Smith			The MetroHealth System, Case Western Reserve University			RECOVER-Adult
Maggie	Washington			The MetroHealth System, Case Western Reserve University			RECOVER-Adult
Elisheva	Weinberger			The MetroHealth System, Case Western Reserve University			RECOVER-Adult
James R.	Heath		PhD	Institute for Systems Biology	Seattle, WA, USA	Hub PI	RECOVER-Adult
Conor	Brennan		BS	Institute for Systems Biology	Seattle, WA, USA	Lab Technician	RECOVER-Adult
Rick	Edmark		BS	Institute for Systems Biology	Seattle, WA, USA	Lab Manager	RECOVER-Adult
Vanessa	Gutierrez		BS	Institute for Systems Biology	Seattle, WA, USA	Lab Technician	RECOVER-Adult
Jennifer	Hadlock		MD	Institute for Systems Biology	Seattle, WA, USA	Co-Investigator	RECOVER-Adult
Sarah	Li		MS	Institute for Systems Biology	Seattle, WA, USA	Lab Technician	RECOVER-Adult
Andrew T.	Magis		PhD	Institute for Systems Biology	Seattle, WA, USA	Co-Investigator	RECOVER-Adult
Connor	McDonald		BS	Institute for Systems Biology	Seattle, WA, USA	Lab Technician	RECOVER-Adult
Kim M.	Murray		PhD	Institute for Systems Biology	Seattle, WA, USA	Program Manager	RECOVER-Adult
Lee	Rowen			Institute for Systems Biology	Seattle, WA, USA	Lab Technician	RECOVER-Adult
Dan	Yuan		PhD	Institute for Systems Biology	Seattle, WA, USA	Research Assistant	RECOVER-Adult
Peter	Chen		MD	Cedars-Sinai Medical Center	Los Angeles, CA, USA	SubSite PI	RECOVER-Adult
Antonina	Caudill		MPH	Cedars-Sinai Medical Center	Los Angeles, CA, USA	Clinical Research Coordinator	RECOVER-Adult
Tananshi	Chopra		BS	Cedars-Sinai Medical Center	Los Angeles, CA, USA	Clinical Research Coordinator	RECOVER-Adult
Fatima	Contreras		BS	Cedars-Sinai Medical Center	Los Angeles, CA, USA	Clinical Research Coordinator	RECOVER-Adult
Lea	Dahlke		BS	Cedars-Sinai Medical Center	Los Angeles, CA, USA	Clinical Research Coordinator	RECOVER-Adult
Lasya	Gudipudi		BS	Cedars-Sinai Medical Center	Los Angeles, CA, USA	Clinical Research Coordinator	RECOVER-Adult

Supplemental Online Content: Nonauthor Collaborators
*First name, last name, and suffix (if applicable) are required and will appear in PubMed.

***First Name and Middle Initial(s)**	***Last Name**	***Suffix (eg, Jr, III)**	Academic Degrees	Institution	Location (city, state/province, country)	Role or Contribution, eg, chair, principal investigator	Group (if more than 1 Group listed in the byline) and/or Subgroup (eg, Steering Committee)
Susan	Jackman		BSN, MS	Cedars-Sinai Medical Center	Los Angeles, CA, USA	Clinical Research Nurse	RECOVER-Adult
Matthew	Modes		MD	Cedars-Sinai Medical Center	Los Angeles, CA, USA	Co-Investigator	RECOVER-Adult
Nicole	Muttera		BS	Cedars-Sinai Medical Center	Los Angeles, CA, USA	Clinical Research Intern	RECOVER-Adult
Chloe	Nelson		BS	Cedars-Sinai Medical Center	Los Angeles, CA, USA	Study Coordinator	RECOVER-Adult
Tanyalak	Parimon		MD	Cedars-Sinai Medical Center	Los Angeles, CA, USA	Co-Investigator	RECOVER-Adult
Nancy	Salinas		BSN	Cedars-Sinai Medical Center	Los Angeles, CA, USA	Clinical Research Nurse	RECOVER-Adult
Josie	Tadeo		BS	Cedars-Sinai Medical Center	Los Angeles, CA, USA	Clinical Research Coordinator	RECOVER-Adult
Sam	Torbati		MD	Cedars-Sinai Medical Center	Los Angeles, CA, USA	Co-Investigator	RECOVER-Adult
Sara	Watson		BS	Cedars-Sinai Medical Center	Los Angeles, CA, USA	Study Coordinator	RECOVER-Adult
Katherine R.	Tuttle		MD	Providence Inland Northwest Health	Spokane, WA, USA	SubSite PI	RECOVER-Adult
Radica	Alicic		MD, FHM, FACP	Providence Inland Northwest Health	Spokane, WA, USA	Co-Investigator	RECOVER-Adult
Joni	Baxter		BS	Providence Inland Northwest Health	Spokane, WA, USA	Clinical Research Coordinator	RECOVER-Adult
Sarah	Emerson		BS	Providence Inland Northwest Health	Spokane, WA, USA	Clinical Research Coordinator	RECOVER-Adult
Susan	Hood		PhD	Providence Inland Northwest Health	Spokane, WA, USA	Regulatory	RECOVER-Adult
Kelli	Kuykendall		NCMA	Providence Inland Northwest Health	Spokane, WA, USA	Clinical Research Coordinator	RECOVER-Adult
Shane	White		BS	Providence Inland Northwest Health	Spokane, WA, USA	Clinical Research Coordinator	RECOVER-Adult
Lauren E.	Wilcox		CMA	Providence Inland Northwest Health	Spokane, WA, USA	Clinical Research Coordinator	RECOVER-Adult

Supplemental Online Content: Nonauthor Collaborators
*First name, last name, and suffix (if applicable) are required and will appear in PubMed.

***First Name and Middle Initial(s)**	***Last Name**	***Suffix (eg, Jr, III)**	Academic Degrees	Institution	Location (city, state/province, country)	Role or Contribution, eg, chair, principal investigator	Group (if more than 1 Group listed in the byline) and/or Subgroup (eg, Steering Committee)
Jason D.	Goldman		MD, MPH	Providence Swedish Medical Center	Seattle, WA, USA	SubSite PI	RECOVER-Adult
Heather A.	Algren		BS, BSN	Providence Swedish Medical Center	Seattle, WA, USA	Site Lead, non-PI	RECOVER-Adult
James	Del Alcazar			Providence Swedish Medical Center	Seattle, WA, USA	Clinical Research Coordinator	RECOVER-Adult
Alexandria M.	Duven		BSN	Providence Swedish Medical Center	Seattle, WA, USA	Research Nurse	RECOVER-Adult
John	Kaneko			Providence Swedish Medical Center	Seattle, WA, USA	Regulatory	RECOVER-Adult
Christina	Kim		MSN, MPH	Providence Swedish Medical Center	Seattle, WA, USA	Co-Investigator	RECOVER-Adult
Paula	Manner			Providence Swedish Medical Center	Seattle, WA, USA	Clinical Research Coordinator	RECOVER-Adult
Carly	Mason		BS	Providence Swedish Medical Center	Seattle, WA, USA	Regulatory	RECOVER-Adult
Ashley	Okada			Providence Swedish Medical Center	Seattle, WA, USA	Sub-Investigator	RECOVER-Adult
Rachel	Poussier		BS, BA	Providence Swedish Medical Center	Seattle, WA, USA	Research Assistant	RECOVER-Adult
Richard	Satira		MSCR	Providence Swedish Medical Center	Seattle, WA, USA	Research Nurse	RECOVER-Adult
Julie A.	Wallick		BS, BA	Providence Swedish Medical Center	Seattle, WA, USA	Clinical Research Coordinator	RECOVER-Adult
Helen Y.	Chu		MD, MPH	University of Washington	Seattle, WA, USA	SubSite PI	RECOVER-Adult
Anna	Elias-Warren		MPH	University of Washington	Seattle, WA, USA	Clinical Research Coordinator	RECOVER-Adult
Alex	Harteloo		BS	University of Washington	Seattle, WA, USA	Clinical Research Coordinator	RECOVER-Adult
Jennifer K.	Logue		BS	University of Washington	Seattle, WA, USA	Project Manager	RECOVER-Adult

*First name, last name, and suffix (if applicable) are required and will appear in PubMed.

***First Name and Middle Initial(s)**	***Last Name**	***Suffix (eg, Jr, III)**	Academic Degrees	Institution	Location (city, state/province, country)	Role or Contribution, eg, chair, principal investigator	Group (if more than 1 Group listed in the byline) and/or Subgroup (eg, Steering Committee)
Kathryn	McCaffrey		BS	University of Washington	Seattle, WA, USA	Clinical Research Coordinator	RECOVER-Adult
Helen	Nguyen		BS	University of Washington	Seattle, WA, USA	Clinical Research Coordinator	RECOVER-Adult
Anoop M.	Nambiar		MD, MS	University of Texas Health Science Center at San Antonio	San Antonio, TX, USA	SubSite PI, Co-Investigator	RECOVER-Adult
Thomas F.	Patterson		MD	University of Texas Health San Antonio	San Antonio, TX, USA	Hub PI	RECOVER-Adult
Jennifer S.	Potter		PhD	University of Texas Health Science Center at San Antonio	San Antonio, TX, USA	Hub PI	RECOVER-Adult
Marzieh	Salehi		MD, MS	University of Texas Health at San Antonio	San Antonio, TX, USA	Hub PI	RECOVER-Adult
Kumar	Sharma		MD	University of Texas Health at San Antonio	San Antonio, TX, USA	SubSite PI	RECOVER-Adult
Monica	Verduzco-Gutierrez		MD	University of Texas Health Science Center at San Antonio	San Antonio, TX, USA	Hub PI	RECOVER-Adult
Reed	Anderson						RECOVER-Adult
Azaneth	Arellanes						RECOVER-Adult
Rose A.	Barajas						RECOVER-Adult
Suneet P.	Chauhan		MD, Hon DSc	University of Texas Health Science Center at Houston, Children's Memorial Hermann Hospital	Houston, TX, USA	Investigator	RECOVER-Adult
Geoffrey D.	Clarke		PhD	University of Texas Health Science Center at San Antonio	San Antonio, TX, USA	Co-Investigator	RECOVER-Adult
Cheryl E.	Farner		MSN	The University of Texas Health Science Center	San Antonio, TX, USA	Co-Investigator	RECOVER-Adult
Melinda S.	Fischer		BS	University of Texas Health Science Center at San Antonio	San Antonio, TX, USA	Clinical Research Coordinator	RECOVER-Adult
Mark P.	Goldberg						RECOVER-Adult
Gabrielyd	Hastings						RECOVER-Adult

*First name, last name, and suffix (if applicable) are required and will appear in PubMed.

***First Name and Middle Initial(s)**	***Last Name**	***Suffix (eg, Jr, III)**	Academic Degrees	Institution	Location (city, state/province, country)	Role or Contribution, eg, chair, principal investigator	Group (if more than 1 Group listed in the byline) and/or Subgroup (eg, Steering Committee)
Patricia	Heard						RECOVER-Adult
Jessica	Hernandez		MS	University of Texas Health at San Antonio	San Antonio, TX, USA	Study Coordinator	RECOVER-Adult
Italia	Herrera						RECOVER-Adult
Edgar	Infante						RECOVER-Adult
Hillary	Johnson						RECOVER-Adult
Johnnie	Jones						RECOVER-Adult
Dean L.	Kellogg		MD, PhD	University of Texas Health Science Center at San Antonio	San Antonio, TX, USA	Co-Investigator	RECOVER-Adult
Ellen	Kraig		PhD	University of Texas Health San Antonio	San Antonio, TX, USA	ROA PI	RECOVER-Adult
Lisa	Longoria		BA	University of Texas Health at San Antonio	San Antonio, TX, USA	Study Coordinator	RECOVER-Adult
Emeka	Okafor			University of Texas Health at San Antonio	San Antonio, TX, USA	Research Associate	RECOVER-Adult
Jan E.	Patterson						RECOVER-Adult
Alexis	Pinones						RECOVER-Adult
W. B.	Reeves		MD	University of Texas Health Science Center at San Antonio	San Antonio, TX, USA	Not provided	RECOVER-Adult
Irma	Scholler						RECOVER-Adult
Sudha	Seshadri		MD	University of Texas Health Science Center at San Antonio	San Antonio, TX, USA	Co-Investigator, Lead CSC Neuropsychiatry Pathobiology Working Group	RECOVER-Adult
Pankil	Shah						RECOVER-Adult
Dimpy P.	Shah		MD, PhD	University of Texas Health San Antonio	San Antonio, TX, USA	Co-Investigator	RECOVER-Adult
Marlaysha	Smith			University of Texas Health Science Center at San Antonio	San Antonio, TX, USA	Research Assistant	RECOVER-Adult
Bridgette	Soileau						RECOVER-Adult

***First Name and Middle Initial(s)**	***Last Name**	***Suffix (eg, Jr, III)**	Academic Degrees	Institution	Location (city, state/province, country)	Role or Contribution, eg, chair, principal investigator	Group (if more than 1 Group listed in the byline) and/or Subgroup (eg, Steering Committee)
Pamela	Solis						RECOVER-Adult
Carmen	Stoebner						RECOVER-Adult
Michael	Sullivan						RECOVER-Adult
Barbara S.	Taylor						RECOVER-Adult
Robin	Tragus						RECOVER-Adult
Joel	Tsevat		MD, MPH	Long School of Medicine, University of Texas Health Science Center at San Antonio	San Antonio, TX, USA	Co-Investigator	RECOVER-Adult
Keren	Hasbani		MD	Dell Children's Medical Center, The University of Texas at Austin	Austin, TX, USA	SubSite PI	RECOVER-Adult
George R.	Saade		MD	The University of Texas Medical Branch	Galveston, TX, USA	SubSite PI	RECOVER-Adult
Kavita	Sharma		MD	University of Texas Southwestern	Dallas, TX, USA	SubSite PI	RECOVER-Adult
Claudia C.	Paredes					PI	RECOVER-Adult
Stephanie	Alvarado						RECOVER-Adult
Andre	Kumar					Hub PI	RECOVER-Adult
Yvonne	Maldonado		MD	Stanford University	Palo Alto, CA, USA	Hub Co-PI	RECOVER-Adult
Paul J.	Utz		MD	Stanford University School of Medicine	Stanford, CA, USA	Hub PI	RECOVER-Adult
Catherine A.	Blish		MD, PhD	Stanford University School of Medicine	Stanford, CA, USA	SubSite PI	RECOVER-Adult
Minjoung	Go		MD, MPH	Stanford School of Medicine	Palo Alto, CA, USA	SubSite PI	RECOVER-Adult
Prasanna	Jagannathan		MD	Stanford University School of Medicine	Stanford, CA, USA	SubSite PI, Co-Investigator, Mechanistic pathways member	RECOVER-Adult
Upinder	Singh		MD	Stanford University	Stanford, CA, USA	Co-PI	RECOVER-Adult

*First name, last name, and suffix (if applicable) are required and will appear in PubMed.

***First Name and Middle Initial(s)**	***Last Name**	***Suffix (eg, Jr, III)**	Academic Degrees	Institution	Location (city, state/province, country)	Role or Contribution, eg, chair, principal investigator	Group (if more than 1 Group listed in the byline) and/or Subgroup (eg, Steering Committee)
Neera	Ahuja		MD	Stanford University School of Medicine	Palo Alto, CA, USA	Long COVID/RECOVER patient caregiver Representative, Co-Investigator, fund CRC/faculty time beyond grant allowance	RECOVER-Adult
Andra L.	Blomkalns		MD, MBA	Stanford University	Stanford, CA, USA	Co-Investigator, Chair-PPOC	RECOVER-Adult
Hector	Bonilla		MD	Stanford University	Palo Alto, CA, USA	Co-Investigator	RECOVER-Adult
Richard	Brotherton						RECOVER-Adult
Kimberly	Clinton						RECOVER-Adult
Vaidehi	Dingankar						RECOVER-Adult
Linda N.	Geng		MD, PhD	Stanford University	Stanford, CA, USA	Co-Investigator	RECOVER-Adult
Francois	Haddad		MD	Stanford University	Palo Alto, CA, USA	Co-Investigator	RECOVER-Adult
Christopher	Jamero		MA	Stanford University	Stanford, CA, USA	CRC2	RECOVER-Adult
Kathryn	Jee						RECOVER-Adult
Xiaolin K.	Jia		MD	Stanford University	Palo Alto, CA, USA	Co-Investigator	RECOVER-Adult
Naresh	Khurana						RECOVER-Adult
Mitchell G.	Miglis		MD	Stanford University	Palo Alto, CA, USA	Co-Investigator	RECOVER-Adult
Ellen	O'Conor						RECOVER-Adult
Kelly	Olszewski						RECOVER-Adult
Divya	Pathak		BA	Stanford University	Palo Alto, CA, USA	Committee Member; CRM	RECOVER-Adult
Orlando	Quintero		MD	Stanford University	Palo Alto, CA, USA	Co-Investigator	RECOVER-Adult
Corey	Saperia		MD	Stanford University School of Medicine			RECOVER-Adult
Jake	Scott		MD	Stanford University	Palo Alto, CA, USA	Co-Investigator	RECOVER-Adult
Alfredo E.	Urdaneta		MD	Stanford University	Palo Alto, CA, USA	Co-Investigator	RECOVER-Adult
Mary R.	Varkey						RECOVER-Adult

Supplemental Online Content: Nonauthor Collaborators
*First name, last name, and suffix (if applicable) are required and will appear in PubMed.

***First Name and Middle Initial(s)**	***Last Name**	***Suffix (eg, Jr, III)**	Academic Degrees	Institution	Location (city, state/province, country)	Role or Contribution, eg, chair, principal investigator	Group (if more than 1 Group listed in the byline) and/or Subgroup (eg, Steering Committee)
Janko Z.	Nikolich		MD, PhD	University of Arizona College of Medicine	Tucson, AZ, USA	MPI	RECOVER-Adult
Sairam	Parthasarathy		MD	The University of Arizona College of Medicine Tucson	Tucson, AZ, USA	MPI	RECOVER-Adult
Kacey C.	Ernst		PhD, MPH	The University of Arizona		Co-Investigator	RECOVER-Adult
Denise R.	Esquivel						RECOVER-Adult
David T.	Harris						RECOVER-Adult
Stefanie	Harris						RECOVER-Adult
Michael	Hernandez						RECOVER-Adult
Harvey	Hsu		MD	University of Arizona	Phoenix, AZ, USA	Co-Investigator	RECOVER-Adult
Michelle	James						RECOVER-Adult
Maria	Karnafel						RECOVER-Adult
Kenneth S.	Knox		MD	University of Arizona	Phoenix, AZ, USA	Co-Investigator	RECOVER-Adult
Alison	Koleski						RECOVER-Adult
Bonnie	LaFleur						RECOVER-Adult
Brenda	Lambert						RECOVER-Adult
Sicily	LaRue						RECOVER-Adult
Karen	Lutrick						RECOVER-Adult
Nirav	Merchant						RECOVER-Adult
Christopher	Morton						RECOVER-Adult
Jarrod M.	Mosier		MD	University of Arizona College of Medicine-Tucson	Tucson, AZ, USA	Co-Investigator	RECOVER-Adult
Toluwanimi	Olorunnisola						RECOVER-Adult
Jeanette	Peralta						RECOVER-Adult
William (.	Pilling						RECOVER-Adult
Kristen	Pogreba-Brown		PhD	University of Arizona	Tucson, AZ, USA	Co-Investigator	RECOVER-Adult
Franz P.	Rischard		DO, MSc	University of Arizona	Tucson, AZ, USA	Co-Investigator	RECOVER-Adult
Lee T.	Ryan						RECOVER-Adult
Terry	Smith						RECOVER-Adult

*First name, last name, and suffix (if applicable) are required and will appear in PubMed.

***First Name and Middle Initial(s)**	***Last Name**	***Suffix (eg, Jr, III)**	Academic Degrees	Institution	Location (city, state/province, country)	Role or Contribution, eg, chair, principal investigator	Group (if more than 1 Group listed in the byline) and/or Subgroup (eg, Steering Committee)
Manuel	Snyder						RECOVER-Adult
Vignesh	Subbian		PhD	University of Arizona	Tucson, AZ, USA	Co-Investigator, Chair of the QA/QC/DI governance committee, Informatics lead for University of Arizona hub	RECOVER-Adult
Kyle	Suhr						RECOVER-Adult
Deanna	Velarde						RECOVER-Adult
Eric M.	Reiman		MD	Banner Alzheimer's Institute	Phoenix, AZ, USA	Sub-site PI	RECOVER-Adult
Joyce K.	Lee-Iannotti		MD	Banner University Medical Center-Phoenix, University of Arizona College of Medicine Phoenix	Phoenix, AZ, USA	Long COVID/RECOVER patient Representative, Long COVID/RECOVER community Representative, Hub PI, SubSite PI, Co-Investigator	RECOVER-Adult
Lynn	Autry						RECOVER-Adult
Hassan	Beydoun			Banner University Medical Center-Phoenix, University of Arizona College of Medicine Phoenix	Phoenix, AZ, USA	Cardiologist Lead	RECOVER-Adult
Sabine	Borwege						RECOVER-Adult
Jacquelynn	Copeland						RECOVER-Adult
Marjorie	DiLise-Russo						RECOVER-Adult
Susan	Fadden						RECOVER-Adult

Supplemental Online Content: Nonauthor Collaborators
*First name, last name, and suffix (if applicable) are required and will appear in PubMed.

***First Name and Middle Initial(s)**	***Last Name**	***Suffix (eg, Jr, III)**	Academic Degrees	Institution	Location (city, state/province, country)	Role or Contribution, eg, chair, principal investigator	Group (if more than 1 Group listed in the byline) and/or Subgroup (eg, Steering Committee)
Isaias	Gomez						RECOVER-Adult
Garrett	Grischo						RECOVER-Adult
William	Hartley						RECOVER-Adult
Leah	Hillier						RECOVER-Adult
Hira	Ismail						RECOVER-Adult
Stephanie	Iusim						RECOVER-Adult
Michelle	James						RECOVER-Adult
Mrinalini	Kala						RECOVER-Adult
Daniel	Kim						RECOVER-Adult
Ganesh	Murthy						RECOVER-Adult
Samuel	Unzek						RECOVER-Adult
Sheila	Vadovicky						RECOVER-Adult
Sharry	Veres						RECOVER-Adult
Christian	Bime						RECOVER-Adult
Lillian	Hansen						RECOVER-Adult
Trina	Hughes						RECOVER-Adult
David	Lieberman						RECOVER-Adult
Francisco	Soto						RECOVER-Adult
Cathleen	Wilson						RECOVER-Adult
Alyssa	Zapien		BS			Clinical Research Coordinator	RECOVER-Adult
Steven G.	Deeks		MD	University of California San Francisco	San Francisco, CA, USA	Hub PI	RECOVER-Adult
John D.	Kelly		MD, PhD	University of California San Francisco	San Francisco, CA, USA	Hub PI, SubSite PI, Co-Investigator	RECOVER-Adult
Jeffrey N.	Martin		MD, MPH	University of California San Francisco	San Francisco, CA, USA	Hub PI, SubSite PI, Co-Investigator	RECOVER-Adult
Michael J.	Peluso		MD MPhil MHS DTM&H	University of California San Francisco	San Francisco, CA, USA	Hub PI, SubSite PI, Co-Investigator	RECOVER-Adult

*First name, last name, and suffix (if applicable) are required and will appear in PubMed.

***First Name and Middle Initial(s)**	***Last Name**	***Suffix (eg, Jr, III)**	Academic Degrees	Institution	Location (city, state/province, country)	Role or Contribution, eg, chair, principal investigator	Group (if more than 1 Group listed in the byline) and/or Subgroup (eg, Steering Committee)
Grace	Anderson		BA	University of California San Francisco	San Francisco, CA, USA	Clinical Research Coordinator	RECOVER-Adult
Khamal	Anglin		MD, MPH	University of California San Francisco	San Francisco, CA, USA	Study Manager	RECOVER-Adult
Urania	Argueta		BS	University of California San Francisco	San Francisco, CA, USA	Clinical Research Coordinator	RECOVER-Adult
Kofi	Asare		BA	University of California San Francisco	San Francisco, CA, USA	Clinical Research Coordinator	RECOVER-Adult
Melissa	Buitrago			University of California San Francisco	San Francisco, CA, USA	Clinical Research Coordinator	RECOVER-Adult
Celina	Chang Song		AA	University of California San Francisco	San Francisco, CA, USA	Clinical Research Coordinator	RECOVER-Adult
Alexus	Clark		BA	University of California San Francisco	San Francisco, CA, USA	Clinical Research Coordinator	RECOVER-Adult
Emily	Conway		BA	University of California San Francisco	San Francisco, CA, USA	Clinical Research Coordinator	RECOVER-Adult
Nicole	Del Castillo			University of California San Francisco	San Francisco, CA, USA	Clinical Research Coordinator	RECOVER-Adult
Monika	Deswal			University of California San Francisco	San Francisco, CA, USA	Clinical Research Coordinator	RECOVER-Adult
Matthew S.	Durstenfeld		MD, MAS	University of California San Francisco	San Francisco, CA, USA	Co-Investigator	RECOVER-Adult
Elnaz	Eilkhani			University of California San Francisco	San Francisco, CA, USA	Regulatory	RECOVER-Adult
Avery	Eun			University of California San Francisco	San Francisco, CA, USA	Clinical Research Coordinator	RECOVER-Adult
Emily	Fehrman		BA	University of California San Francisco	San Francisco, CA, USA	Clinical Research Coordinator	RECOVER-Adult
Tony	Figueroa		BA	University of California San Francisco	San Francisco, CA, USA	Clinical Research Coordinator	RECOVER-Adult

*First name, last name, and suffix (if applicable) are required and will appear in PubMed.

***First Name and Middle Initial(s)**	***Last Name**	***Suffix (eg, Jr, III)**	Academic Degrees	Institution	Location (city, state/province, country)	Role or Contribution, eg, chair, principal investigator	Group (if more than 1 Group listed in the byline) and/or Subgroup (eg, Steering Committee)
Diana	Flores		MPH	University of California San Francisco	San Francisco, CA, USA	Clinical Research Coordinator	RECOVER-Adult
Halle	Grebe		BS	University of California San Francisco	San Francisco, CA, USA	Clinical Research Coordinator	RECOVER-Adult
Timothy J.	Henrich		MD, MMSc	University of California San Francisco	San Francisco, CA, USA	Co-Investigator	RECOVER-Adult
Rebecca	Hoh		MS, RD	University of California San Francisco	San Francisco, CA, USA	Clinical Research Coordinator	RECOVER-Adult
Priscilla	Hsue		MD	University of California San Francisco	San Francisco, CA, USA	Co-Investigator	RECOVER-Adult
Beatrice	Huang		BA	University of California San Francisco	San Francisco, CA, USA	Study Manager	RECOVER-Adult
Rania	Ibrahim			University of California San Francisco	San Francisco, CA, USA	Clinical Research Coordinator	RECOVER-Adult
Marian	Kerbleski		RN	University of California San Francisco	San Francisco, CA, USA	Clinical Research Coordinator	RECOVER-Adult
Raushun	Kirtikar			University of California San Francisco	San Francisco, CA, USA	Clinical Research Coordinator	RECOVER-Adult
Megan T.	Lew		BA	University of California San Francisco	San Francisco, CA, USA	Clinical Research Coordinator	RECOVER-Adult
James	Lombardo			University of California San Francisco	San Francisco, CA, USA	Clinical Research Coordinator	RECOVER-Adult
Monica	Lopez		BA	University of California San Francisco	San Francisco, CA, USA	Clinical Research Coordinator	RECOVER-Adult
Michael	Luna			University of California San Francisco	San Francisco, CA, USA	Clinical Research Coordinator	RECOVER-Adult
Carina	Marquez		MD, MPH	University of California San Francisco	San Francisco, CA, USA	Co-Investigator	RECOVER-Adult
Sadie	Munter		BA	University of California San Francisco	San Francisco, CA, USA	Clinical Research Coordinator	RECOVER-Adult

Supplemental Online Content: Nonauthor Collaborators
*First name, last name, and suffix (if applicable) are required and will appear in PubMed.

*First Name and Middle Initial(s)	*Last Name	*Suffix (eg, Jr, III)	Academic Degrees	Institution	Location (city, state/province, country)	Role or Contribution, eg, chair, principal investigator	Group (if more than 1 Group listed in the byline) and/or Subgroup (eg, Steering Committee)
Lynn	Ngo		BA	University of California San Francisco	San Francisco, CA, USA	Clinical Research Coordinator	RECOVER-Adult
Jesus	Pineda-Ramirez		BA	University of California San Francisco	San Francisco, CA, USA	Clinical Research Coordinator	RECOVER-Adult
Aric	Prather		PhD, BA	University of California San Francisco	San Francisco, CA, USA	Co-Investigator	RECOVER-Adult
Kim	Rhoads		MD, MS, MPH, FACS	University of California San Francisco	San Francisco, CA, USA	Co-Investigator	RECOVER-Adult
Antonio	Rodriguez		BA	University of California San Francisco	San Francisco, CA, USA	Clinical Research Coordinator	RECOVER-Adult
Justin	Romero		BA	University of California San Francisco	San Francisco, CA, USA	Clinical Research Coordinator	RECOVER-Adult
Dylan	Ryder		BA	University of California San Francisco	San Francisco, CA, USA	Clinical Research Coordinator	RECOVER-Adult
Matthew	So		MD	University of California San Francisco	San Francisco, CA, USA	Clinical Research Coordinator	RECOVER-Adult
Ma	Somsouk		MD, MAS	University of California San Francisco	San Francisco, CA, USA	Co-Investigator	RECOVER-Adult
Viva	Tai		RD, MPH	University of California San Francisco	San Francisco, CA, USA	Clinical Research Coordinator	RECOVER-Adult
Brandon	Tran		BA	University of California San Francisco	San Francisco, CA, USA	Clinical Research Coordinator	RECOVER-Adult
Julian	Uy		BA	University of California San Francisco	San Francisco, CA, USA	Clinical Research Coordinator	RECOVER-Adult
Daisy	Valdivieso		BS	University of California San Francisco	San Francisco, CA, USA	Clinical Research Coordinator	RECOVER-Adult
Deepshika	Verma		BA	University of California San Francisco	San Francisco, CA, USA	Clinical Research Coordinator	RECOVER-Adult
Meghann	Williams		BSN, RN	University of California San Francisco	San Francisco, CA, USA	Research Nurse	RECOVER-Adult

Supplemental Online Content: Nonauthor Collaborators
*First name, last name, and suffix (if applicable) are required and will appear in PubMed.

***First Name and Middle Initial(s)**	***Last Name**	***Suffix (eg, Jr, III)**	Academic Degrees	Institution	Location (city, state/province, country)	Role or Contribution, eg, chair, principal investigator	Group (if more than 1 Group listed in the byline) and/or Subgroup (eg, Steering Committee)
Andhy	Zamora		MS	University of California San Francisco	San Francisco, CA, USA	Clinical Research Coordinator	RECOVER-Adult
Lisa T.	Newman			RTI International	MD, USA	Principal Investigator/Project Director	Administrative Coordinating Center
Julie	Abella		MA, PMP	RTI International	NC, USA	Oversight & Monitoring Lead, Project Manager	Administrative Coordinating Center
Quinn	Barnette			RTI International	NC, USA	COG and BAC Committee Coordinator	Administrative Coordinating Center
Christine	Bevc		PhD	RTI International	FL, USA	Application Review Lead, R3 Seminar Moderator	Administrative Coordinating Center
Jennifer	Beverly		BS, BA	RTI International	NC, USA	Autospy CC Facilitator	Administrative Coordinating Center
Patricia	Ceger			RTI International	NC, USA	Interventions Task Force Facilitator	Administrative Coordinating Center
Julie	Croxford		MPH	RTI International	MD, USA	ASOC Facilitator; Systems Biology WG Facilitator	Administrative Coordinating Center
Emily	Cunningham			RTI International	NC, USA	Project Administration Specialist, Investigator Review Payments	Administrative Coordinating Center
Mike	Enger			RTI International	NC, USA	Omics Task Force Coordinator	Administrative Coordinating Center
Katie	Fain			RTI International	NC, USA	Integrative Physiology Task Force Coordinator	Administrative Coordinating Center

Supplemental Online Content: Nonauthor Collaborators
*First name, last name, and suffix (if applicable) are required and will appear in PubMed.

***First Name and Middle Initial(s)**	***Last Name**	***Suffix (eg, Jr, III)**	Academic Degrees	Institution	Location (city, state/province, country)	Role or Contribution, eg, chair, principal investigator	Group (if more than 1 Group listed in the byline) and/or Subgroup (eg, Steering Committee)
Tonya	Farris			RTI International	DC, USA	Governance Committee Support Lead	Administrative Coordinating Center
Sean	Hanlon			RTI International	NC, USA	Informatics Co-Lead and Web Portal Architect	Administrative Coordinating Center
David	Hines			RTI International	NC, USA	Mechanistic Pathways Task Force Facilitator	Administrative Coordinating Center
Vicki	Johnson-Lawrence		PhD	RTI International	NC, USA	Representative Engagement Co-Lead	Administrative Coordinating Center
Kevin	Jordan			RTI International	OR, USA	Mechanistic Pathways Task Force Coordinator	Administrative Coordinating Center
Craig	Lefebvre		PhD	RTI International	AZ, USA	Communications Lead	Administrative Coordinating Center
Beth	Linas			RTI International	DC, USA	Lead Science Communication Expert	Administrative Coordinating Center
Bryan	Luukinen		MSPH	RTI International	NC, USA	Communications, Content Lead	Administrative Coordinating Center
Meisha	Mandal			RTI International	NC, USA	Omics and Integrative Physiology Task Force Facilitator	Administrative Coordinating Center
Nikki J.	McKoy		MBA, MPH	RTI International	GA, USA	Representative Engagement Co-Lead	Administrative Coordinating Center

Supplemental Online Content: Nonauthor Collaborators
*First name, last name, and suffix (if applicable) are required and will appear in PubMed.

***First Name and Middle Initial(s)**	***Last Name**	***Suffix (eg, Jr, III)**	Academic Degrees	Institution	Location (city, state/province, country)	Role or Contribution, eg, chair, principal investigator	Group (if more than 1 Group listed in the byline) and/or Subgroup (eg, Steering Committee)
Susan	Nance			RTI International	NC, USA	Population Science Task Force Coordinator, PIPP Behavioral and Rehabilitation Subcommittee Coordinator	Administrative Coordinating Center
Ashleigh	Oakland			RTI International	NC, USA	Project Administration Specialist, Representative Review Payments	Administrative Coordinating Center
Demian	Pasquarelli		BA	RTI International	NC, USA	Informatics Co-Lead /REDCap Data Collection	Administrative Coordinating Center
Claire	Quiner			RTI International	NC, USA	Commonalities with Other Post-Viral Syndromes Task Force Facilitator & PIPP Biologics Subcommittee Facilitator	Administrative Coordinating Center
Rita	Sembajwe			RTI International	GA, USA	Committee Support Sub-task Lead and PIPP Drug and Rehabilitation Subcommittee Facilitator	Administrative Coordinating Center
Gwendolyn	Shaw			RTI International	NC, USA	Interventions Task Force Coordinator	Administrative Coordinating Center

Supplemental Online Content: Nonauthor Collaborators
*First name, last name, and suffix (if applicable) are required and will appear in PubMed.

***First Name and Middle Initial(s)**	***Last Name**	***Suffix (eg, Jr, III)**	Academic Degrees	Institution	Location (city, state/province, country)	Role or Contribution, eg, chair, principal investigator	Group (if more than 1 Group listed in the byline) and/or Subgroup (eg, Steering Committee)
Vanessa	Thornburg			RTI International	NC, USA	Commonalities with Other Post-Viral Syndromes Task Force Coordinator and PIPP Complementary and Alternative Medicine Subcomittee Facilitator	Administrative Coordinating Center
Kendall	Tosco			RTI International	NC, USA	OSMB Coordinator	Administrative Coordinating Center
Hannah	Wright		MSPH	RTI International	CA, USA	Application Review Co-Lead	Administrative Coordinating Center
Rachel S.	Gross		MD	NYU Grossman School of Medicine	New York, NY, USA	mPI	Clinical Science Core
Judith S.	Hochman		MD	NYU Grossman School of Medicine	New York, NY, USA	mPI	Clinical Science Core
Leora I.	Horwitz		MD	NYU Grossman School of Medicine	New York, NY, USA	mPI	Clinical Science Core
Stuart D.	Katz		MD	NYU Grossman School of Medicine	New York, NY, USA	mPI	Clinical Science Core
Andrea B.	Troxel		MD	NYU Grossman School of Medicine	New York, NY, USA	mPI	Clinical Science Core
Lenard	Adler			NYU Langone Health	New York, NY, USA	Co-Investigator	Clinical Science Core
Precious	Akinbo			NYU Langone Health	New York, NY, USA	Clinical Research Associate, Research Progam Manager	Clinical Science Core

Supplemental Online Content: Nonauthor Collaborators
*First name, last name, and suffix (if applicable) are required and will appear in PubMed.

***First Name and Middle Initial(s)**	***Last Name**	***Suffix (eg, Jr, III)**	Academic Degrees	Institution	Location (city, state/province, country)	Role or Contribution, eg, chair, principal investigator	Group (if more than 1 Group listed in the byline) and/or Subgroup (eg, Steering Committee)
Ramona	Almenana			NYU Langone Health	New York, NY, USA	Assistant Program Director of Comms and OEC Liaison	Clinical Science Core
Ola	Bello			NYU Langone Health	New York, NY, USA	Data Analyst/SAS Programmer	Clinical Science Core
Sultana	Bhuiyan			NYU Langone Health	New York, NY, USA	Clinical Trial Assistant	Clinical Science Core
Nina	Blachman			NYU Langone Health	New York, NY, USA	Co-Investigator	Clinical Science Core
Ryan	Branski			NYU Langone Health	New York, NY, USA	Co-Investigator	Clinical Science Core
Jasmine	Briscoe			NYU Langone Health	New York, NY, USA	Research Coordinator	Clinical Science Core
Shari	Brosnahan			NYU Langone Health	New York, NY, USA	Co-Investigator	Clinical Science Core
Elliott	Bueler			NYU Langone Health	New York, NY, USA	Senior Research Project Manager	Clinical Science Core
Yvette	Burgos			NYU Langone Health	New York, NY, USA	Senior Program Coordinator	Clinical Science Core
Nina	Caplin			NYU Langone Health	New York, NY, USA	Co-Investigator	Clinical Science Core
Domonique N.	Chaplin		MS	NYU Langone Health	New York, NY, USA	Senior Research Project Manager, Publications & External Affairs	Clinical Science Core
Yu	Chen			NYU Langone Health	New York, NY, USA	Co-Investigator	Clinical Science Core
Shen	Cheng			NYU Langone Health	New York, NY, USA	Data Analyst	Clinical Science Core
Peter	Choe			NYU Langone Health	New York, NY, USA	Financial Analyst	Clinical Science Core
Jess	Choi			NYU Langone Health	New York, NY, USA	Project Manager	Clinical Science Core
Alicia	Chung			NYU Langone Health	New York, NY, USA	Co-Investigator	Clinical Science Core
Richard	Church			NYU Langone Health	New York, NY, USA	MCIT Technical Project Manager	Clinical Science Core

*First name, last name, and suffix (if applicable) are required and will appear in PubMed.

*First Name and Middle Initial(s)	*Last Name	*Suffix (eg, Jr, III)	Academic Degrees	Institution	Location (city, state/province, country)	Role or Contribution, eg, chair, principal investigator	Group (if more than 1 Group listed in the byline) and/or Subgroup (eg, Steering Committee)
Stanley	Cobos			NYU Langone Health	New York, NY, USA	Clinical Research Associate, Research Progam Manager	Clinical Science Core
Nakia	Croft			NYU Langone Health	New York, NY, USA	Clinical Research Associate	Clinical Science Core
Angelique	Cruz Irving			NYU Langone Health	New York, NY, USA	Clinical Research Associate	Clinical Science Core
Phoebe	Del Boccio			NYU Langone Health	New York, NY, USA	Assistan Program Director Autopsy/Peds/Compliance Peds	Clinical Science Core
Iván	Díaz			NYU Langone Health	New York, NY, USA	Co-Investigator	Clinical Science Core
Vishal	Doshi			NYU Langone Health	New York, NY, USA	Co-Investigator	Clinical Science Core
Benard	Dreyer			NYU Langone Health	New York, NY, USA		Clinical Science Core
Samantha	Ebel			NYU Langone Health	New York, NY, USA	Director Contracts	Clinical Science Core
Arline	Faustin			New York University Grossman School of Medicine, NYU Tisch Hospital	New York, NY, USA	Co-Investigator	Clinical Science Core
Elias	Febres			NYU Langone Health	New York, NY, USA	Senior Program Coordinator	Clinical Science Core
Jeffrey	Fine			NYU Langone Health	New York, NY, USA	Co-Investigator	Clinical Science Core
Sandra	Fink			NYU Langone Health	New York, NY, USA	Contracts Manager	Clinical Science Core
Jennifer	Frontera			NYU Langone Health	New York, NY, USA	Co-Investigator	Clinical Science Core
Richard	Gallagher			NYU Langone Health	New York, NY, USA	Co-Investigator	Clinical Science Core
Alejandra	Gonzalez-Duarte			NYU Langone Health	New York, NY, USA	Co-Investigator	Clinical Science Core
Denise	Hasson			NYU Langone Health	New York, NY, USA	Co-Investigator	Clinical Science Core
Sophia	Hill			NYU Langone Health	New York, NY, USA	Project Manager	Clinical Science Core
Shahidul	Islam			NYU Langone Health	New York, NY, USA	Co-Investigator	Clinical Science Core
Stephen	Johnson			NYU Langone Health	New York, NY, USA	Co-Investigator	Clinical Science Core

*First name, last name, and suffix (if applicable) are required and will appear in PubMed.

***First Name and Middle Initial(s)**	***Last Name**	***Suffix (eg, Jr, III)**	Academic Degrees	Institution	Location (city, state/province, country)	Role or Contribution, eg, chair, principal investigator	Group (if more than 1 Group listed in the byline) and/or Subgroup (eg, Steering Committee)
Neha	Kansal			NYU Langone Health	New York, NY, USA		Clinical Science Core
Rachel	Kenney			NYU Langone Health	New York, NY, USA	Co-Investigator	Clinical Science Core
Deepshikha	Kewlani			New York University Grossman School of Medicine	New York, NY, USA	Research Coordinator	Clinical Science Core
Michelle F.	Lamendola-Essel			New York University Grossman School of Medicine	New York, NY, USA	Program Director, Observational Studies Operations	Clinical Science Core
Gregory	Laynor			NYU Langone Health	New York, NY, USA	Co-Investigator	Clinical Science Core
Terry	Leon			NYU Langone Health	New York, NY, USA	Neuropsych Adult neurocognitive eval (for nih toolbox and cognitive testing)	Clinical Science Core
Zoe A.	Lewczak		BS	New York University Grossman School of Medicine	New York, NY, USA	Program Coordinator	Clinical Science Core
Janelle	Linton			NYU Langone Health	New York, NY, USA	Assistant Program Director of CE and CE Liaison	Clinical Science Core
Max	Logan			NYU Langone Health	New York, NY, USA	Senior Program Coordinator	Clinical Science Core
Nadia	Malik			NYU Langone Health	New York, NY, USA	Clinical Research Associate	Clinical Science Core
Lia	Mamistvalova			NYU Langone Health	New York, NY, USA	Research Nurse	Clinical Science Core
Hannah	Mandel			NYU Langone Health	New York, NY, USA	Senior Research Scientist	Clinical Science Core
Gabrielle	Maranga			NYU Langone Health	New York, NY, USA	Assistant Program Director of Adult/Compliance, Adult	Clinical Science Core
Patenne D.	Mathews		MPH	New York University Grossman School of Medicine	New York, NY, USA	Program Coordinator	Clinical Science Core

Supplemental Online Content: Nonauthor Collaborators
*First name, last name, and suffix (if applicable) are required and will appear in PubMed.

*First Name and Middle Initial(s)	*Last Name	*Suffix (eg, Jr, III)	Academic Degrees	Institution	Location (city, state/province, country)	Role or Contribution, eg, chair, principal investigator	Group (if more than 1 Group listed in the byline) and/or Subgroup (eg, Steering Committee)
Aprajita	Mattoo			NYU Langone Health	New York, NY, USA	Co-Investigator	Clinical Science Core
Tony	Mei			NYU Langone Health	New York, NY, USA	Core Data personnel	Clinical Science Core
Alan	Mendelsohn			NYU Langone Health	New York, NY, USA	Co-Investigator	Clinical Science Core
Emmanuelle	Mercier			NYU Langone Health	New York, NY, USA	Contracts Manager	Clinical Science Core
Patricio	Millar Vernetti			NYU Langone Health	New York, NY, USA	Co-Investigator	Clinical Science Core
Marc	Miller			NYU Langone Health	New York, NY, USA	Financial Analyst	Clinical Science Core
Maika	Mitchell			NYU Langone Health	New York, NY, USA	Senior Director	Clinical Science Core
Andre	Moreira			NYU Langone Health	New York, NY, USA	Co-Investigator	Clinical Science Core
Praveen C.	Mudumbi		MD	New York University Grossman School of Medicine	New York, NY, USA	Project Manager	Clinical Science Core
Erica	Nahin			NYU Langone Health	New York, NY, USA	Neuropsych PhD	Clinical Science Core
Nandini	Nair			NYU Langone Health	New York, NY, USA	Co-Investigator	Clinical Science Core
Joseph	Nekulak			NYU Langone Health	New York, NY, USA	MCIT Senior Programmer	Clinical Science Core
Kellie	Owens			NYU Langone Health	New York, NY, USA	Co-Investigator	Clinical Science Core
Brendan	Parent			NYU Langone Health	New York, NY, USA	Co-Investigator	Clinical Science Core
Nandan	Patibandla			NYU Langone Health	New York, NY, USA	MCIT System Administrator	Clinical Science Core
Peter	Petrov			NYU Langone Health	New York, NY, USA	Senior Financial Analyst	Clinical Science Core
Radu	Postelnicu			NYU Langone Health	New York, NY, USA	Co-Investigator	Clinical Science Core
Isabelle	Randall			NYU Langone Health	New York, NY, USA	Clinical Trial Assistant	Clinical Science Core
Priyatha	Rao			NYU Langone Health	New York, NY, USA	Senior Contracts Specialist	Clinical Science Core
Amy	Rapkiewicz			NYU Langone Health	New York, NY, USA	Co-Investigator	Clinical Science Core
JohnRoss	Rizzo			NYU Langone Health	New York, NY, USA	Co-Investigator	Clinical Science Core
Johana	Rosas			NYU Langone Health	New York, NY, USA	Neuropsych PhD	Clinical Science Core

Supplemental Online Content: Nonauthor Collaborators
*First name, last name, and suffix (if applicable) are required and will appear in PubMed.

***First Name and Middle Initial(s)**	***Last Name**	***Suffix (eg, Jr, III)**	Academic Degrees	Institution	Location (city, state/province, country)	Role or Contribution, eg, chair, principal investigator	Group (if more than 1 Group listed in the byline) and/or Subgroup (eg, Steering Committee)
Chelsea	Rose			NYU Langone Health	New York, NY, USA	Program Coordinator	Clinical Science Core
Christina	Saint-Jean			NYU Langone Health	New York, NY, USA	Project Manager	Clinical Science Core
Michelle	Santacatterina			NYU Langone Health	New York, NY, USA	Co-Investigator	Clinical Science Core
Binita	Shah			NYU Langone Health	New York, NY, USA	Co-Investigator	Clinical Science Core
Aasma	Shaukat			NYU Langone Health	New York, NY, USA	Co-Investigator	Clinical Science Core
Naomi	Simon			NYU Langone Health	New York, NY, USA	Co-Investigator	Clinical Science Core
Aylin	Simsir			NYU Langone Health	New York, NY, USA	Co-Investigator	Clinical Science Core
Miranda	Stinson			NYU Langone Health	New York, NY, USA	Program Coordinator	Clinical Science Core
Wenfei	Tang			NYU Langone Health	New York, NY, USA	Senior Financial Analyst	Clinical Science Core
Vasishta	Tatapudi			NYU Langone Health	New York, NY, USA	Co-Investigator	Clinical Science Core
Sujata	Thawani			NYU Langone Health	New York, NY, USA	Co-Investigator	Clinical Science Core
Mary	Thomas			NYU Langone Health	New York, NY, USA	Administrative Manager	Clinical Science Core
Lorna	Thorpe			New York University Grossman School of Medicine	New York, NY, USA	Co-Investigator	Clinical Science Core
MeeLee	Tom			NYU Langone Health	New York, NY, USA		Clinical Science Core
Ethan	Treiha			NYU Langone Health	New York, NY, USA	Research Coordinator	Clinical Science Core
Jennifer	Truong			NYU Langone Health	New York, NY, USA	Senior Project Manager	Clinical Science Core
Mmekom	Udosen			NYU Langone Health	New York, NY, USA		Clinical Science Core
Jessica	Velazquez-Perez			NYU Langone Health	New York, NY, USA	Program Coordinator	Clinical Science Core
Patricio M.	Vernetti			NYU Langone Health	New York, NY, USA		Clinical Science Core
Crystal	Vidal			NYU Langone Health	New York, NY, USA	Senior Research Project Manager	Clinical Science Core
Anand	Viswanathan			NYU Langone Health	New York, NY, USA	Co-Investigator	Clinical Science Core

*First name, last name, and suffix (if applicable) are required and will appear in PubMed.

***First Name and Middle Initial(s)**	***Last Name**	***Suffix (eg, Jr, III)**	Academic Degrees	Institution	Location (city, state/province, country)	Role or Contribution, eg, chair, principal investigator	Group (if more than 1 Group listed in the byline) and/or Subgroup (eg, Steering Committee)
Crystal	Wong			NYU Langone Health	New York, NY, USA		Clinical Science Core
Marion J.	Wood		MPH, BS	New York University Grossman School of Medicine	New York, NY, USA	Research Coordinator	Clinical Science Core
Shannon W.	Wuller			NYU Langone Health	New York, NY, USA	Project Manager	Clinical Science Core
Shonna H.	Yin			NYU Langone Health	New York, NY, USA	Co-Investigator	Clinical Science Core
Chloe	Young			New York University Grossman School of Medicine	New York, NY, USA	Program Coordinator	Clinical Science Core
Jonah	Zaretsky			NYU Langone Health	New York, NY, USA	Co-Investigator	Clinical Science Core
Susanna	Zavlunova			NYU Langone Health	New York, NY, USA	Senior Project Manager Safety Monitoring	Clinical Science Core
Andrea	Foulkes		ScD	Massachusetts General Hospital	Boston, MA, USA	Principal Investigator	Data Resource Core
Elizabeth W.	Karlson		MD	Brigham and Women's Hospital	Boston, MA, USA	Principal Investigator	Data Resource Core
Shawn	Murphy		MD, PhD	Massachusetts General Hospital	Boston, MA, USA	Principal Investigator	Data Resource Core
Shreya	Ahirwar			Massachusetts General Hospital	Boston, MA, USA	Biostats	Data Resource Core
Shifa	Ahmed			Massachusetts General Hospital	Boston, MA, USA	Biostats	Data Resource Core
Layne L.	Ainsworth			Brigham and Women's Hospital	Boston, MA, USA	Project Manager	Data Resource Core
Rachel	Atchley-Challenner		PhD	Massachusetts General Hospital	Boston, MA, USA	Biostats	Data Resource Core
Paul	Avilach			Harvard Medical School	Boston, MA, USA		Data Resource Core
Trisha T.	Balan			Massachusetts General Hospital	Boston, MA, USA	Biostats	Data Resource Core
Nicholas	Benik			Massachusetts General Hospital	Boston, MA, USA	Data Portals	Data Resource Core
Barbara	Benoit			Massachusetts General Hospital	Boston, MA, USA	Data Portals	Data Resource Core
Marie-Abèle C.	Bind			Massachusetts General Hospital	Boston, MA, USA	Biostats	Data Resource Core
William J.	Bonaventura			Massachusetts General Hospital	Boston, MA, USA	Biostats	Data Resource Core
Natalie	Boutin			Massachusetts General Hospital	Boston, MA, USA	Leadership	Data Resource Core
Beverly	Brion			Massachusetts General Hospital	Boston, MA, USA	Biostats	Data Resource Core
Andrew	Cagan			Massachusetts General Hospital	Boston, MA, USA	Data Portals	Data Resource Core

Supplemental Online Content: Nonauthor Collaborators
*First name, last name, and suffix (if applicable) are required and will appear in PubMed.

***First Name and Middle Initial(s)**	***Last Name**	***Suffix (eg, Jr, III)**	Academic Degrees	Institution	Location (city, state/province, country)	Role or Contribution, eg, chair, principal investigator	Group (if more than 1 Group listed in the byline) and/or Subgroup (eg, Steering Committee)
Tianrun	Cai			Brigham and Women's Hospital	Boston, MA, USA	Biostats	Data Resource Core
Tingyi	Cao			Massachusetts General Hospital	Boston, MA, USA	Biostats	Data Resource Core
Victor M.	Castro			Massachusetts General Hospital	Boston, MA, USA	Data Portals	Data Resource Core
Xander R.	Cerretani			Brigham and Women's Hospital	Boston, MA, USA	Project Management	Data Resource Core
James G.	Chan			Massachusetts General Hospital	Boston, MA, USA	Project Management	Data Resource Core
David	Cheng			Massachusetts General Hospital	Boston, MA, USA	Biostats	Data Resource Core
Lori B.	Chibnik			Massachusetts General Hospital	Boston, MA, USA	Biostats	Data Resource Core
Mark	Ciriello			Harvard Medical School	Boston, MA, USA	Data Portals	Data Resource Core
Karen	Costenbader		MD, MPH	Brigham and Women's Hospital	Boston, MA, USA	Biostats	Data Resource Core
Dimitar S.	Dimitrov			Massachusetts General Hospital	Boston, MA, USA	Cloud & FISMA	Data Resource Core
Hossein	Estiri		PhD	Massachusetts General Hospital	Boston, MA, USA	Data Portals	Data Resource Core
Maria	Fayad			Massachusetts General Hospital	Boston, MA, USA	Biostats	Data Resource Core
Candace H.	Feldman		MD, ScD	Brigham and Women's Hospital	Boston, MA, USA	Biostats	Data Resource Core
Vivian	Gainer			Massachusetts General Hospital	Boston, MA, USA	Project Manager	Data Resource Core
Bhaswati	Ghosh			Massachusetts General Hospital	Boston, MA, USA	Data Portals	Data Resource Core
Randy	Gollub			Massachusetts General Hospital	Boston, MA, USA	Data Portals	Data Resource Core
Zoe	Guan			Massachusetts General Hospital	Boston, MA, USA	Biostats	Data Resource Core
Alan	Harris			Harvard Medical School	Boston, MA, USA	Data Portals	Data Resource Core
Karl	Helmer			Massachusetts General Hospital	Boston, MA, USA	Data Portals	Data Resource Core
Andrew	Hendrix	III		Harvard Medical School	Boston, MA, USA	Data Portals	Data Resource Core
Ana	Holzbach			Brigham and Women's Hospital	Boston, MA, USA	Data Portals	Data Resource Core
Weixing	Huang			Massachusetts General Hospital	Boston, MA, USA	Biostats	Data Resource Core
Daniel	Kaufman			Massachusetts General Hospital	Boston, MA, USA	Biostats	Data Resource Core
Diane	Keogh			Harvard Medical School	Boston, MA, USA	Data Portals	Data Resource Core
James D.	Kerr			Brigham and Women's Hospital	Boston, MA, USA	Project Management	Data Resource Core
Jeffrey G.	Klann			Massachusetts General Hospital	Boston, MA, USA	Data Portals	Data Resource Core
Aparna	Krishnamoorthy			Massachusetts General Hospital	Boston, MA, USA	Biostats	Data Resource Core

Supplemental Online Content: Nonauthor Collaborators
*First name, last name, and suffix (if applicable) are required and will appear in PubMed.

*First Name and Middle Initial(s)	*Last Name	*Suffix (eg, Jr, III)	Academic Degrees	Institution	Location (city, state/province, country)	Role or Contribution, eg, chair, principal investigator	Group (if more than 1 Group listed in the byline) and/or Subgroup (eg, Steering Committee)
Jessica A.	Lasky-Su		ScD	Brigham and Women's Hospital	Boston, MA, USA	Biostats	Data Resource Core
Katherine P.	Liao		MD, MPH	Brigham and Women's Hospital	Boston, MA, USA	Biostats	Data Resource Core
Doug	MacFadden			Harvard Medical School	Boston, MA, USA	Data Portals	Data Resource Core
Anupama	Maram			Harvard Medical School	Boston, MA, USA	Data Portals	Data Resource Core
Megan W.	Martel			Massachusetts General Hospital	Boston, MA, USA	Biostats	Data Resource Core
Michael	Mendis			Massachusetts General Hospital	Boston, MA, USA	Data Portals	Data Resource Core
Reeta	Metta			Massachusetts General Hospital	Boston, MA, USA	Data Portals	Data Resource Core
Jonathan	Monteiro			Massachusetts General Hospital	Boston, MA, USA	Biostats	Data Resource Core
Eduardo	Morales			Massachusetts General Hospital	Boston, MA, USA	Data Portals	Data Resource Core
Richard E.	Morse			Massachusetts General Hospital	Boston, MA, USA	Biostats	Data Resource Core
Marc-Danie	Nazaire			Harvard Medical School	Boston, MA, USA	Data Portals	Data Resource Core
Gregory	Neils			Massachusetts General Hospital	Boston, MA, USA	Cloud & FISMA	Data Resource Core
Amber N.	Nguyen			Massachusetts General Hospital	Boston, MA, USA	Biostats	Data Resource Core
James	Norman			Harvard Medical School	Boston, MA, USA	Cloud & FISMA	Data Resource Core
Henry H.	Paik			Massachusetts General Hospital	Boston, MA, USA	Biostats	Data Resource Core
Deepti	Pant			Massachusetts General Hospital	Boston, MA, USA	Biostats	Data Resource Core
Heekyong	Park			Massachusetts General Hospital	Boston, MA, USA	Cloud & FISMA	Data Resource Core
Dustin J.	Rabideau			Massachusetts General Hospital	Boston, MA, USA	Biostats	Data Resource Core
Harrison T.	Reeder			Massachusetts General Hospital	Boston, MA, USA	Biostats	Data Resource Core
Kathleen	Rossi-Roh			Massachusetts General Hospital	Boston, MA, USA	Project Management	Data Resource Core
Leah M.	Santacroce		MA	Brigham and Women's Hospital	Boston, MA, USA	Biostats	Data Resource Core
Katherine	Schlepphorst			Massachusetts General Hospital	Boston, MA, USA	Biostats	Data Resource Core
Carolin	Schulte			Massachusetts General Hospital	Boston, MA, USA	Biostats	Data Resource Core
Caitlin A.	Selvaggi			Massachusetts General Hospital	Boston, MA, USA	Biostats	Data Resource Core
Daniel J.	Shinnick			Massachusetts General Hospital	Boston, MA, USA	Biostats	Data Resource Core
William	Simons			Massachusetts General Hospital	Boston, MA, USA	Biostats	Data Resource Core
Lynn A.	Simpson			Massachusetts General Hospital	Boston, MA, USA	Cloud & FISMA	Data Resource Core
Mary L.	St. Jean Flanders			Massachusetts General Hospital	Boston, MA, USA	Biostats	Data Resource Core
Zachary	Strasser			Massachusetts General Hospital	Boston, MA, USA	Data Portals	Data Resource Core

Supplemental Online Content: Nonauthor Collaborators
*First name, last name, and suffix (if applicable) are required and will appear in PubMed.

***First Name and Middle Initial(s)**	***Last Name**	***Suffix (eg, Jr, III)**	Academic Degrees	Institution	Location (city, state/province, country)	Role or Contribution, eg, chair, principal investigator	Group (if more than 1 Group listed in the byline) and/or Subgroup (eg, Steering Committee)
Mansi R.	Thakrar			Massachusetts General Hospital	Boston, MA, USA	Biostats	Data Resource Core
Tanayott	Thaweethai			Massachusetts General Hospital	Boston, MA, USA	Biostats	Data Resource Core
Madeleine	Thorn			Massachusetts General Hospital	Boston, MA, USA	Biostats	Data Resource Core
Philip	Trewett			Harvard Medical School	Boston, MA, USA	Data Portals	Data Resource Core
Dustin	Van Fleet			Brigham and Women's Hospital	Boston, MA, USA	Project Management	Data Resource Core
Kavishwar B.	Wagholikar			Massachusetts General Hospital	Boston, MA, USA	Data Portals	Data Resource Core
Taowei D.	Wang			Massachusetts General Hospital	Boston, MA, USA	Data Portals	Data Resource Core
Nich	Wattanasin			Massachusetts General Hospital	Boston, MA, USA	Cloud & FISMA	Data Resource Core
Griffin	Weber			Massachusetts General Hospital	Boston, MA, USA	Data Portals	Data Resource Core
Michael A.	Williams			Massachusetts General Hospital	Boston, MA, USA	Data Portals	Data Resource Core
Ren Zhe	Zhang			Boston Children's Hospital	Boston, MA, USA	Biostats	Data Resource Core
Marta	Cerda				USA	Co-Chair	National Community Engagement Group
Victor H.	Clash				USA	Co-Chair	National Community Engagement Group
Felicia	Davis Blakley				USA	Co-Chair	National Community Engagement Group
Brittany	Taylor				USA	Co-Chair	National Community Engagement Group
Mike	Zissis				USA	Co-Chair	National Community Engagement Group
Teresa	Akintonwa				USA		National Community Engagement Group
Frank	Blancero				USA		National Community Engagement Group
Heather-Elizabeth	Brown				USA		National Community Engagement Group
Megan	Carmilani				USA		National Community Engagement Group

Supplemental Online Content: Nonauthor Collaborators
*First name, last name, and suffix (if applicable) are required and will appear in PubMed.

***First Name and Middle Initial(s)**	***Last Name**	***Suffix (eg, Jr, III)**	Academic Degrees	Institution	Location (city, state/province, country)	Role or Contribution, eg, chair, principal investigator	Group (if more than 1 Group listed in the byline) and/or Subgroup (eg, Steering Committee)
Debra	Copeland				USA		National Community Engagement Group
Yvonka	Hall				USA		National Community Engagement Group
kevin	kondo				USA		National Community Engagement Group
Lydia	Lerma				USA		National Community Engagement Group
Jacqui	Lindsay				USA		National Community Engagement Group
Heather	Marti				USA		National Community Engagement Group
Christine	Maughan				USA		National Community Engagement Group
Tony	Minor				USA		National Community Engagement Group
Hyatt	Vincent				USA		National Community Engagement Group
Jeffrey P.	Burns		MD, MPH	Boston Children's Hospital	Boston, MA, USA	Co-Chair	Observational Consortium Steering Committee (OCSC)
Serena	Spudich		MD, MA	Yale School of Medicine	New Haven, CT, USA	Co-Chair	Observational Consortium Steering Committee (OCSC)
Charles	Bailey		MD, PhD	Children's Hospital of Philadelphia	Philadelphia, PA, USA	Convening Chair of the EHR Studies Coordinating Committee	Observational Consortium Steering Committee (OCSC)
Mine	Cicek		PhD	Mayo Clinic	Rochester, MN, USA	Principal Investigator, Biorepository Core	Observational Consortium Steering Committee (OCSC)

Supplemental Online Content: Nonauthor Collaborators
*First name, last name, and suffix (if applicable) are required and will appear in PubMed.

***First Name and Middle Initial(s)**	***Last Name**	***Suffix (eg, Jr, III)**	Academic Degrees	Institution	Location (city, state/province, country)	Role or Contribution, eg, chair, principal investigator	Group (if more than 1 Group listed in the byline) and/or Subgroup (eg, Steering Committee)
Melissa M.	Cortez		DO	University of Utah	Salt Lake City, UT, USA	Subject Matter Expert	Observational Consortium Steering Committee (OCSC)
Felicia	Davis Blakley				USA	Patient, Caregiver and Community Representative	Observational Consortium Steering Committee (OCSC)
Andrea S.	Foulkes		ScD		Boston, MA, USA	Principal Investigator, Data Resource Core	Observational Consortium Steering Committee (OCSC)
David	Goff		MD, PhD		USA	Senior Scientific Program Director From the National Institutes of Health (NIH)	Observational Consortium Steering Committee (OCSC)
Stuart D.	Katz		MD	NYU Grossman School of Medicine	New York, NY, USA	Principal Investigator, Clinical Science Core	Observational Consortium Steering Committee (OCSC)
Jessica	Lasky-Su		DSc, MS		USA	Subject Matter Expert	Observational Consortium Steering Committee (OCSC)
Torri D.	Metz		MD, MS	University of Utah Health Sciences	Salt Lake City, UT, USA	Convening Chair of the Pregnancy Cohort Coordinating Committee	Observational Consortium Steering Committee (OCSC)
Lisa T.	Newman		MSPH	RTI International	, MD, USA	Principal Investigator, Administrative Coordinating Center	Observational Consortium Steering Committee (OCSC)

Supplemental Online Content: Nonauthor Collaborators
*First name, last name, and suffix (if applicable) are required and will appear in PubMed.

***First Name and Middle Initial(s)**	***Last Name**	***Suffix (eg, Jr, III)**	Academic Degrees	Institution	Location (city, state/province, country)	Role or Contribution, eg, chair, principal investigator	Group (if more than 1 Group listed in the byline) and/or Subgroup (eg, Steering Committee)
Igho	Ofotokun		MD	Emory University	Atlanta, GA, USA	Convening Chair of the Adult Cohort Coordinating Committee	Observational Consortium Steering Committee (OCSC)
Sudha	Seshadri		MD, DM		USA	Subject Matter Expert	Observational Consortium Steering Committee (OCSC)
Melissa	Stockwell		MD, MPH	Columbia University Irving Medical Center	New York, NY, USA	Convening Chair of the Pediatric Cohort Coordinating Committee	Observational Consortium Steering Committee (OCSC)
James	Stone		MD, PhD	Massachusetts General Hospital, Harvard Medical School	Boston, MA, USA	Convening Chair of the Autopsy Cohort Coordinating Committee	Observational Consortium Steering Committee (OCSC)
Brittany D.	Taylor		MPH		USA	Patient, Caregiver and Community Representative	Observational Consortium Steering Committee (OCSC)
PJ	Utz		MD	Stanford University School of Medicine	Stanford, CA, USA	Subject Matter Expert	Observational Consortium Steering Committee (OCSC)
Neely A.	Williams		MDiv, EdD		USA	Patient, Caregiver and Community Representative	Observational Consortium Steering Committee (OCSC)
Brett	Anderson			Columbia University Irving Medical Center			Health Equity/ PRO / Community Engagement
Sujata	Bardhan						Health Equity/ PRO / Community Engagement

*First name, last name, and suffix (if applicable) are required and will appear in PubMed.

***First Name and Middle Initial(s)**	***Last Name**	***Suffix (eg, Jr, III)**	Academic Degrees	Institution	Location (city, state/province, country)	Role or Contribution, eg, chair, principal investigator	Group (if more than 1 Group listed in the byline) and/or Subgroup (eg, Steering Committee)
Leah	Castro-Baucom						Health Equity/ PRO / Community Engagement
Deena	Chisolm						Health Equity/ PRO / Community Engagement
Alicia	Chung			NYU Langone Health			Health Equity/ PRO / Community Engagement
Claudia	Corchado						Health Equity/ PRO / Community Engagement
Casey L.	Daniel			University of South Alabama			Health Equity/ PRO / Community Engagement
Walter	Dehority			University of New Mexico Health Sciences Center			Health Equity/ PRO / Community Engagement
Gniesha	Dinwiddie						Health Equity/ PRO / Community Engagement
Candace H.	Feldman			Brigham and Women's Hospital			Health Equity/ PRO / Community Engagement
Lisa	Goldman Rosas						Health Equity/ PRO / Community Engagement
Carol R.	Horowitz			Icahn School of Medicine at Mount Sinai			Health Equity/ PRO / Community Engagement

Supplemental Online Content: Nonauthor Collaborators
*First name, last name, and suffix (if applicable) are required and will appear in PubMed.

***First Name and Middle Initial(s)**	***Last Name**	***Suffix (eg, Jr, III)**	Academic Degrees	Institution	Location (city, state/province, country)	Role or Contribution, eg, chair, principal investigator	Group (if more than 1 Group listed in the byline) and/or Subgroup (eg, Steering Committee)
Janice	John			Cambridge Health Alliance			Health Equity/ PRO / Community Engagement
Gelise L.	Thomas						Health Equity/ PRO / Community Engagement
Keila	Lopez						Health Equity/ PRO / Community Engagement
Karen	Lutrick						Health Equity/ PRO / Community Engagement
Carina	Marquez			University of California San Francisco			Health Equity/ PRO / Community Engagement
Shelly	MacDonald Pinkett						Health Equity/ PRO / Community Engagement
Lidia	Regino						Health Equity/ PRO / Community Engagement
Kim	Rhoads			University of California San Francisco			Health Equity/ PRO / Community Engagement
Sarah A.	Stewart de Ramirez			UI College of Medicine Peoria			Health Equity/ PRO / Community Engagement
Joel	Tsevat			University of Texas Health Science Center at San Antonio			Health Equity/ PRO / Community Engagement

Supplemental Online Content: Nonauthor Collaborators
*First name, last name, and suffix (if applicable) are required and will appear in PubMed.

***First Name and Middle Initial(s)**	***Last Name**	***Suffix (eg, Jr, III)**	Academic Degrees	Institution	Location (city, state/province, country)	Role or Contribution, eg, chair, principal investigator	Group (if more than 1 Group listed in the byline) and/or Subgroup (eg, Steering Committee)
Carlos	Valencia			NYU Langone Health			Health Equity/ PRO / Community Engagement
Nita	Vangeepuram						Health Equity/ PRO / Community Engagement
Anita	Walden			University of Colorado Anschutz Medical Campus			Health Equity/ PRO / Community Engagement
Zanthia	Wiley			Emory Healthcare (Hope Clinic)			Health Equity/ PRO / Community Engagement
Neely A.	Williams						Health Equity/ PRO / Community Engagement
Shonna H.	Yin			NYU Langone Health			Health Equity/ PRO / Community Engagement
Nina	Blachman			NYU Langone Health			Participant Experience
Natalie	Boutin			Massachusetts General Hospital			Participant Experience
Phoebe	Burton			Rhode Island Hospital			Participant Experience
Marina	Catallozzi			Columbia University Irving Medical Center			Participant Experience
Cheryl R.	Clark			Brigham and Women's Hospital			Participant Experience
Beth	Dworetzky						Participant Experience
Belinda	Edwards						Participant Experience
Robert L.	Ferrer						Participant Experience
Catherine	Freeland			NYU Langone Health			Participant Experience
Beatrice	Huang			University of California San Francisco			Participant Experience

Supplemental Online Content: Nonauthor Collaborators
*First name, last name, and suffix (if applicable) are required and will appear in PubMed.

***First Name and Middle Initial(s)**	***Last Name**	***Suffix (eg, Jr, III)**	Academic Degrees	Institution	Location (city, state/province, country)	Role or Contribution, eg, chair, principal investigator	Group (if more than 1 Group listed in the byline) and/or Subgroup (eg, Steering Committee)
Suzanne E.	Judd			University of Alabama at Birmingham			Participant Experience
Sarah	Laury			NYU Langone Health			Participant Experience
Hugh	Musick			University of Illinois Hospital & Clinics			Participant Experience
Divya	Pathak			Stanford University			Participant Experience
Gail	Pearson						Participant Experience
Kristen	Pogreba-Brown			University of Arizona			Participant Experience
Hengameh	Raissy			University of New Mexico Health Sciences Center			Participant Experience
Lynne	Richardson			Icahn School of Medicine at Mount Sinai			Participant Experience
Russell	Rothman			Vanderbilt University Medical Center			Participant Experience
Laura	Wagner						Participant Experience
Ann	Wallace						Participant Experience
Teresa	Akintonwa				USA		RECOVER Patient, Caregiver, and/or Community Representative
Leyna	Aragon				USA		RECOVER Patient, Caregiver, and/or Community Representative
Bryan	Bander				USA		RECOVER Patient, Caregiver, and/or Community Representative

Supplemental Online Content: Nonauthor Collaborators
*First name, last name, and suffix (if applicable) are required and will appear in PubMed.

***First Name and Middle Initial(s)**	***Last Name**	***Suffix (eg, Jr, III)**	Academic Degrees	Institution	Location (city, state/province, country)	Role or Contribution, eg, chair, principal investigator	Group (if more than 1 Group listed in the byline) and/or Subgroup (eg, Steering Committee)
Karyn	Bishof				USA		RECOVER Patient, Caregiver, and/or Community Representative
Frank	Blancero				USA		RECOVER Patient, Caregiver, and/or Community Representative
Heather-Elizabeth	Brown				USA		RECOVER Patient, Caregiver, and/or Community Representative
Etienne	Carignan				USA		RECOVER Patient, Caregiver, and/or Community Representative
Megan	Carmilani				USA		RECOVER Patient, Caregiver, and/or Community Representative
Leah	Castro Baucom				USA		RECOVER Patient, Caregiver, and/or Community Representative
Marta	Cerda				USA		RECOVER Patient, Caregiver, and/or Community Representative

Supplemental Online Content: Nonauthor Collaborators
*First name, last name, and suffix (if applicable) are required and will appear in PubMed.

***First Name and Middle Initial(s)**	***Last Name**	***Suffix (eg, Jr, III)**	Academic Degrees	Institution	Location (city, state/province, country)	Role or Contribution, eg, chair, principal investigator	Group (if more than 1 Group listed in the byline) and/or Subgroup (eg, Steering Committee)
Victor	Clash				USA		RECOVER Patient, Caregiver, and/or Community Representative
Krista	Coombs				USA		RECOVER Patient, Caregiver, and/or Community Representative
Claudia G.	Corchado				USA		RECOVER Patient, Caregiver, and/or Community Representative
Hannah	Davis				USA		RECOVER Patient, Caregiver, and/or Community Representative
Felicia	Davis Blakley				USA		RECOVER Patient, Caregiver, and/or Community Representative
Marissa	Diggs				USA		RECOVER Patient, Caregiver, and/or Community Representative
Matthew	Dunn				USA		RECOVER Patient, Caregiver, and/or Community Representative

Supplemental Online Content: Nonauthor Collaborators
*First name, last name, and suffix (if applicable) are required and will appear in PubMed.

***First Name and Middle Initial(s)**	***Last Name**	***Suffix (eg, Jr, III)**	Academic Degrees	Institution	Location (city, state/province, country)	Role or Contribution, eg, chair, principal investigator	Group (if more than 1 Group listed in the byline) and/or Subgroup (eg, Steering Committee)
Belinda	Edwards				USA		RECOVER Patient, Caregiver, and/or Community Representative
Liza	Fisher				USA		RECOVER Patient, Caregiver, and/or Community Representative
Megan	Fitzgerald				USA		RECOVER Patient, Caregiver, and/or Community Representative
Margot	Gage Witvliet				USA		RECOVER Patient, Caregiver, and/or Community Representative
Tyler	Gustafson				USA		RECOVER Patient, Caregiver, and/or Community Representative
Yvonka	Hall				USA		RECOVER Patient, Caregiver, and/or Community Representative
Verna	Holmes				USA		RECOVER Patient, Caregiver, and/or Community Representative

Supplemental Online Content: Nonauthor Collaborators
*First name, last name, and suffix (if applicable) are required and will appear in PubMed.

***First Name and Middle Initial(s)**	***Last Name**	***Suffix (eg, Jr, III)**	Academic Degrees	Institution	Location (city, state/province, country)	Role or Contribution, eg, chair, principal investigator	Group (if more than 1 Group listed in the byline) and/or Subgroup (eg, Steering Committee)
Mady	Hornig				USA		RECOVER Patient, Caregiver, and/or Community Representative
Maxwell	Hornig				USA		RECOVER Patient, Caregiver, and/or Community Representative
Nita	Jain				USA		RECOVER Patient, Caregiver, and/or Community Representative
Christina	Kim				USA		RECOVER Patient, Caregiver, and/or Community Representative
Julie	Lam				USA		RECOVER Patient, Caregiver, and/or Community Representative
Lydia	Lerma				USA		RECOVER Patient, Caregiver, and/or Community Representative
Rebecca	Letts				USA		RECOVER Patient, Caregiver, and/or Community Representative

Supplemental Online Content: Nonauthor Collaborators
*First name, last name, and suffix (if applicable) are required and will appear in PubMed.

*First Name and Middle Initial(s)	*Last Name	*Suffix (eg, Jr, III)	Academic Degrees	Institution	Location (city, state/province, country)	Role or Contribution, eg, chair, principal investigator	Group (if more than 1 Group listed in the byline) and/or Subgroup (eg, Steering Committee)
Juan	Lewis				USA		RECOVER Patient, Caregiver, and/or Community Representative
Jacqui	Lindsay				USA		RECOVER Patient, Caregiver, and/or Community Representative
Heather	Marti				USA		RECOVER Patient, Caregiver, and/or Community Representative
Thomas	Martinez				USA		RECOVER Patient, Caregiver, and/or Community Representative
Christine	Maughan				USA		RECOVER Patient, Caregiver, and/or Community Representative
Lisa	McCorkell				USA		RECOVER Patient, Caregiver, and/or Community Representative
Rebecca	McGrath				USA		RECOVER Patient, Caregiver, and/or Community Representative

*First name, last name, and suffix (if applicable) are required and will appear in PubMed.

***First Name and Middle Initial(s)**	***Last Name**	***Suffix (eg, Jr, III)**	Academic Degrees	Institution	Location (city, state/province, country)	Role or Contribution, eg, chair, principal investigator	Group (if more than 1 Group listed in the byline) and/or Subgroup (eg, Steering Committee)
Tony	Minor				USA		RECOVER Patient, Caregiver, and/or Community Representative
Kian	Nguyen				USA		RECOVER Patient, Caregiver, and/or Community Representative
Lauren	Nichols				USA		RECOVER Patient, Caregiver, and/or Community Representative
Lisa	O'Brien				USA		RECOVER Patient, Caregiver, and/or Community Representative
Aimee	Peddie				USA		RECOVER Patient, Caregiver, and/or Community Representative
Alice	Perlowski				USA		RECOVER Patient, Caregiver, and/or Community Representative
Elizabeth	Phillips-Lorenzo				USA		RECOVER Patient, Caregiver, and/or Community Representative

*First name, last name, and suffix (if applicable) are required and will appear in PubMed.

***First Name and Middle Initial(s)**	***Last Name**	***Suffix (eg, Jr, III)**	Academic Degrees	Institution	Location (city, state/province, country)	Role or Contribution, eg, chair, principal investigator	Group (if more than 1 Group listed in the byline) and/or Subgroup (eg, Steering Committee)
Lisa	Prentiss				USA		RECOVER Patient, Caregiver, and/or Community Representative
Nadia	Raytselis				USA		RECOVER Patient, Caregiver, and/or Community Representative
Lidia	Regino				USA		RECOVER Patient, Caregiver, and/or Community Representative
Megan	Rockwell				USA		RECOVER Patient, Caregiver, and/or Community Representative
Jacqueline	Rutter				USA		RECOVER Patient, Caregiver, and/or Community Representative
Elle	Seibert				USA		RECOVER Patient, Caregiver, and/or Community Representative
Anisha	Sekar				USA		RECOVER Patient, Caregiver, and/or Community Representative

Supplemental Online Content: Nonauthor Collaborators
*First name, last name, and suffix (if applicable) are required and will appear in PubMed.

***First Name and Middle Initial(s)**	***Last Name**	***Suffix (eg, Jr, III)**	Academic Degrees	Institution	Location (city, state/province, country)	Role or Contribution, eg, chair, principal investigator	Group (if more than 1 Group listed in the byline) and/or Subgroup (eg, Steering Committee)
Brittany	Taylor				USA		RECOVER Patient, Caregiver, and/or Community Representative
Emily	Taylor				USA		RECOVER Patient, Caregiver, and/or Community Representative
Hyatt	Vincent				USA		RECOVER Patient, Caregiver, and/or Community Representative
Ann	Wallace				USA		RECOVER Patient, Caregiver, and/or Community Representative
Rochelle	Wilensky				USA		RECOVER Patient, Caregiver, and/or Community Representative
Melissa	Williams				USA		RECOVER Patient, Caregiver, and/or Community Representative
Neely	Williams				USA		RECOVER Patient, Caregiver, and/or Community Representative

Supplemental Online Content: Nonauthor Collaborators
*First name, last name, and suffix (if applicable) are required and will appear in PubMed.

***First Name and Middle Initial(s)**	***Last Name**	***Suffix (eg, Jr, III)**	Academic Degrees	Institution	Location (city, state/province, country)	Role or Contribution, eg, chair, principal investigator	Group (if more than 1 Group listed in the byline) and/or Subgroup (eg, Steering Committee)
Kay	Williams-Dawson				USA		RECOVER Patient, Caregiver, and/or Community Representative
Andrew	Wylam				USA		RECOVER Patient, Caregiver, and/or Community Representative
Mike	Zissis				USA		RECOVER Patient, Caregiver, and/or Community Representative
