# Supplementary material for: Sex Differences in Long COVID
Source: JAMA Netw Open. 2025 Jan 22;8(1):e2455430. doi: 10.1001/jamanetworkopen.2024.55430 (PMC11755195; doi:10.1001/jamanetworkopen.2024.55430)
Supplement: Supplement 3. — Data Sharing Statement [file jamanetwopen-e2455430-s003.pdf]

Data Sharing Statement

Shah. Sex Differences in Long COVID. *JAMA Netw Open*. Published January 22, 2025.
doi:10.1001/jamanetworkopen.2024.55430

Data
Data available: Yes
Data types: Deidentified participant data, Data dictionary
How to access data: Because the RECOVER Adult Cohort Observational Study is in progress and the datasets are updated dynamically, individual patient/participant data will not be provided at the time of publication. However, the data dictionary is published on the RECOVER website and a link will be made available. Furthermore, NHLBI has undertaken a significant effort to release harmonized data from all RECOVER observational cohort studies to the public via their BioData Catalyst platform within the next six months. When these data are released, the datasets will include participant-level data collected for the present study, as well as the technical infrastructure and analytic tools to evaluate the results and conclusions from this study. https://recovercovid.org/ https://biodatacatalyst.nhlbi.nih.gov/
When available: beginning date: 04-25-2024

Supporting Documents
Document types: None

Additional Information
Who can access the data: Researchers whose proposed use of the data has been approved
Types of analyses: For a specified scientific purpose
Mechanisms of data availability: After approval of a proposal and with a signed data access agreement
